# Ascorbic acid mitigates the impact of oxidative stress in a human model of febrile seizure and mesial temporal lobe epilepsy

**DOI:** 10.1038/s41598-024-56680-4

**Published:** 2024-03-11

**Authors:** Stefania Scalise, Clara Zannino, Valeria Lucchino, Michela Lo Conte, Vittorio Abbonante, Giorgia Lucia Benedetto, Mariangela Scalise, Antonio Gambardella, Elvira Immacolata Parrotta, Giovanni Cuda

**Affiliations:** 1 Department of Experimental and Clinical Medicine, University Magna Graecia of Catanzaro, Viale Europa, 88100 Catanzaro, Italy; 2 Department of Health Sciences, University Magna Graecia of Catanzaro, Viale Europa, 88100 Catanzaro, Italy; 3 Department of Medical and Surgical Sciences, University Magna Graecia of Catanzaro, Viale Europa, 88100 Catanzaro, Italy

**Keywords:** Neural stem cells, Mechanisms of disease

## Abstract

Prolonged febrile seizures (FS) in children are linked to the development of temporal lobe epilepsy (MTLE). The association between these two pathologies may be ascribed to the long-term effects that FS exert on neural stem cells, negatively affecting the generation of new neurons. Among the insults associated with FS, oxidative stress is noteworthy. Here, we investigated the consequences of exposure to hydrogen peroxide (H_2_O_2_) in an induced pluripotent stem cell-derived neural stem cells (iNSCs) model of a patient affected by FS and MTLE. In our study, we compare the findings from the MTLE patient with those derived from iNSCs of a sibling exhibiting a milder phenotype defined only by FS, as well as a healthy individual. In response to H_2_O_2_ treatment, iNSCs derived from MTLE patients demonstrated an elevated production of reactive oxygen species and increased apoptosis, despite the higher expression levels of antioxidant genes and proteins compared to other cell lines analysed. Among the potential causative mechanisms of enhanced vulnerability of MTLE patient iNSCs to oxidative stress, we found that these cells express low levels of the heat shock protein HSPB1 and of the autophagy adaptor SQSTM1/p62. Pre-treatment of diseased iNSCs with the antioxidant molecule ascorbic acid restored HSBP1 and p62 expression and simultaneously reduced the levels of ROS and apoptosis. Our findings suggest the potential for rescuing the impaired oxidative stress response in diseased iNSCs through antioxidant treatment, offering a promising mechanism to prevent FS degeneration in MTLE.

## Introduction

The generation of new neurons in the mammalian brain through the process of neurogenesis is driven by the neural stem cells (NSCs) population, which possesses the abilities to self-renew and differentiate by asymmetric division into neurons and glial cells^1^. Neurogenesis is not a static process, but rather influenced by various physiological and pathological stimuli^2,3^. Among diseased conditions, early life recurrent febrile seizure (FS) can negatively affect the capability of NSCs residing within the subgranular zone of the hippocampus to yield functional neurons^4–7^. Aberrant neurogenesis following complex FS during infancy represents a significant risk factor for the development of intractable mesial temporal lobe epilepsy (MTLE) with hippocampal sclerosis later in life^8–11^. Several reports have indicated an increase in oxidant molecules in the serum and cerebrospinal fluid of children who experienced FS^12–14^. Specifically, seizures are associated with an up-regulation of reactive oxygen species (ROS)^15^, such as hydrogen peroxide (H_2_O_2_)^16,17^, which is considered a major ROS molecule involved in redox homeostasis^18^. Physiological ROS play a significant role in modulating the functional state of NSCs by maintaining the balance between self-renewal and differentiation^19,20^; however, when the production of ROS exceeds the scavenging capacity of the cell, oxidative stress occurs^21^. Oxidative stress, in turn, can induce cellular damage, impacting various components within the cell and ultimately culminating in cell death^22^. The capability of adult NSCs to counteract oxidative stress requires the presence of efficient stress-response pathways^23^ and a functioning chaperone network that contributes to the maintenance of a healthy proteasome in the presence of exogenous toxic stress, such as H_2_O_2_ treatment^24^. In this work, we aimed to investigate the potential correlation between oxidative stress and NSCs damage by treating induced pluripotent stem cell derived NSCs (iNSCs) with H_2_O_2_. Experiments were performed using cells derived from a healthy individual and from a patient affected by FS and MTLE, carrying an inherited mutation in the *SCN1A* gene (hereafter identified as the SCN1A^severe^ line). The presence of the mutation per se is not sufficient to develop MTLE after experiencing FS, as demonstrated by the absence of MTLE in other members belonging to the same family and sharing the identical mutation^25,26^. These aspects prompted us to hypothesise that additional mechanisms beyond the genetic mutation, such as the capability to respond to stress stimuli, may contribute to the diverse phenotypes observed. To validate this hypothesis, we included into the study iNSCs obtained from the siblings of the aforementioned patient (designated as SCN1A^mild^). This individual carries the same SCN1A mutation but only manifested FS until the age of six and did not develop MTLE. Upon exposure to H_2_O_2_ treatment, iNSCs derived from the patient SCN1A^severe^ exhibited an augmented production of ROS and increased susceptibility to apoptosis, in contrast to healthy iNSCs and SCN1A^mild^ iNSCs. Among the potential mechanisms contributing to the heightened susceptibility of SCN1A^severe^ patient iNSCs to oxidative stress, we identified that these cells express significantly low levels of the heat shock protein HSPB1 and the selective autophagy adaptor SQSTM1/p62. These two proteins form a complex that plays a significant role during stress, promoting the clearance of damaged organelles and proteins through autophagic flux^27–30^. Interestingly, treating iNSCs with ascorbic acid (AA), a well-known antioxidant and neuroprotective molecule, led to the restoration of HSBP1 and p62 expression in SCN1A^severe^ patient-derived cells while simultaneously reducing the production of ROS and preventing apoptosis. These results showed the potential to counterbalance the impaired oxidative stress response in diseased iNSCs by treating cells with antioxidant compounds, offering a plausible mechanism to prevent the degeneration of FS in MTLE. Moreover, our study establishes a foundation to demonstrate that the progression of FS to MTLE hinges on an individual’s capacity to respond to stressors, particularly oxidative stress.

## Results

### SCN1A^severe^ iNSCs show increased ROS generation and altered antioxidant responses

**Figure 1:**
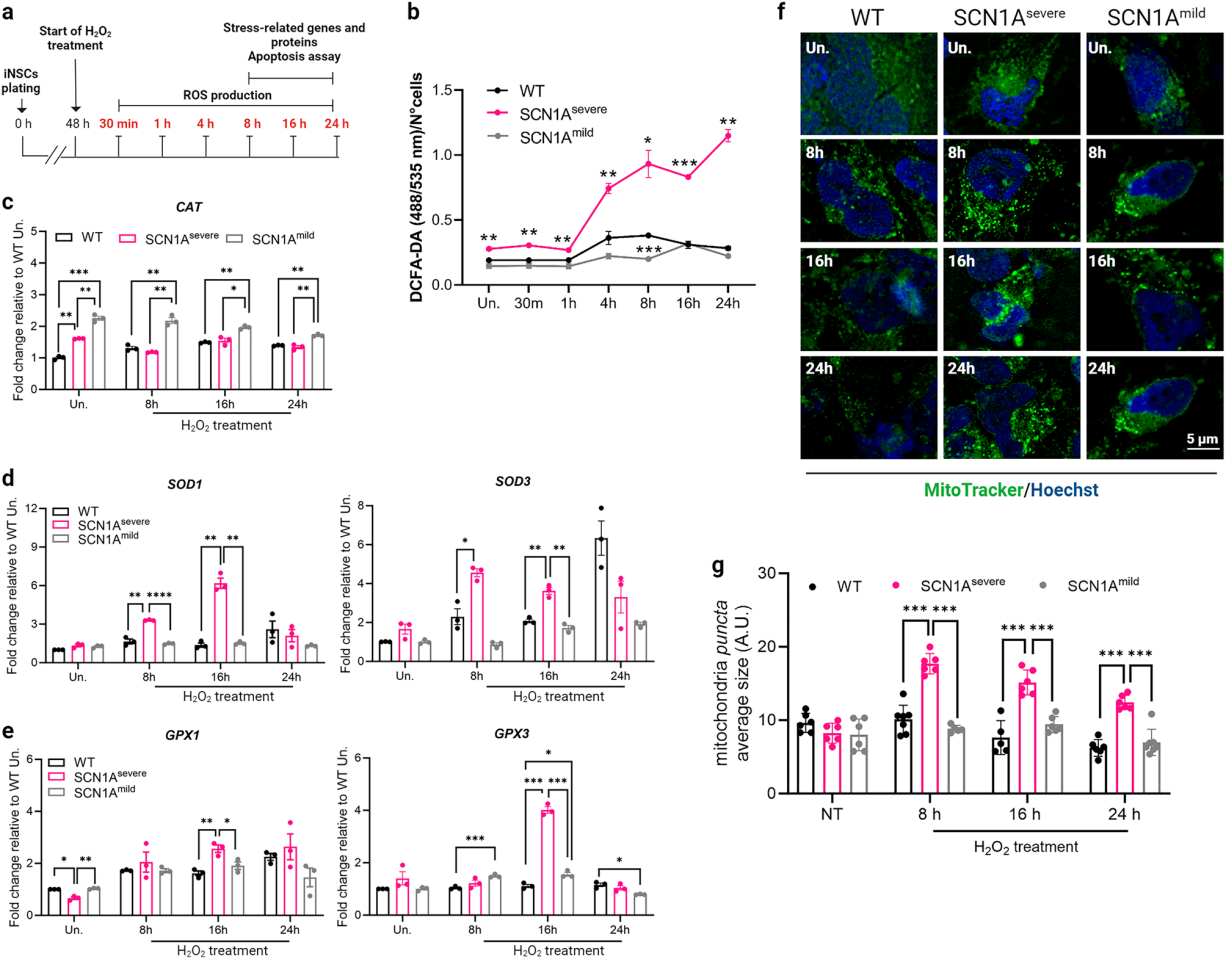
Effects of H_2_O_2_ treatment on iNSCs. (**a**) Schematic diagram showing experimental design for H_2_O_2_ treatment on iNSCs. (**b**) Healthy subject and epileptic patients iNSCs were treated with 2 mM H_2_O_2_ in a range of time from 30 min to 24 h. The production of ROS was assessed at each time point using DCFA-DA fluorescence, and a significantly higher level of ROS production in SCN1A^severe^ patient compared to healthy and SCN1A^mild^ subjects was observed across all tested time intervals. DCFA-DA signal of each well was normalised to the number of cells in the same well. Data are presented as mean ± SEM of three biological replicates, *p < 0.05, **p < 0.01, ***p < 0.001, t-test has been calculated vs. WT in the same time point. (**c**) Analysis of expression of *CAT* gene in diseased and WT iNSCs under H_2_O_2_ treatment showed a significant increase only in SCN1A^mild^ cells. (**d**) The expression of superoxide dismutase genes *SOD1* and *SOD3* was significantly up-regulated in SCN1A^severe^ patient iNSCs in respect to healthy and SCN1A^mild^ cells at 8 and 16 h of H_2_O_2_ treatment, while (**e**) genes codifying for glutathione peroxidases *GPX1* and *GPX3* became significantly up-regulated in SCN1A^severe^ patient iNSCs in respect to healthy and SCN1A^mild^ cells at 16 h of H_2_O_2_ treatments. For (**c**–**e**), *GAPDH* was used as a housekeeping gene and data are represented as mean ± SEM of three biological replicates. *p < 0.05, **p < 0.01, ***p < 0.001, ****p < 0.0001, t-test. (**f**) Representative image of iNSCs stained with MitoTracker Green under H_2_O_2_ treatment of 8, 16 and 24 h. (**g**) The quantification of the size of mitochondria *puncta* showed a significant accumulation of these organelles under H_2_O_2_ treatment in SCN1A^severe^ iNSCs in respect to WT and SCN1A^mild^ iNSCs. At least six images per condition were analysed. ***p < 0.001, t-test.

To investigate the effect of oxidative stress on iNSCs, we initially generated NSCs from iPSCs derived from both two patients and a healthy control. The generated iNSCs exhibited the expression of the typical NSC markers NESTIN and PAX6 (Supplementary fig. S1), confirming their NSCs identity. ROS have been shown to activate multiple stress pathways in NSCs, therefore we first examined ROS production in both patients-derived iNSCs and healthy iNSCs after treatment with 2 mM H_2_O_2_ for a duration ranging from 30 min to 24 h (Fig. 1a). Analysis using 2′-7′-Dichlorodihydrofluorescein diacetate (DCFA-DA) fluorescence revealed that ROS generation was significantly higher in SCN1A^severe^-iNSCs compared to healthy and SCN1A^mild^ iNSCs, both at the basal level and upon H_2_O_2_ treatment at all tested time points (Fig. 1b). These data suggest that in severely diseased cells, an imbalanced oxidant-antioxidant system may occur. We carried out subsequent experiments at 8-, 16- and 24 h after exposure to H_2_O_2_, during which we observed the most significant accumulation of ROS. The levels of H_2_O_2_ in cells are predominantly regulated by the activity of catalase, which catalyzes the conversion of H_2_O_2_ molecules into oxygen and water^31^. Upon H_2_O2_2_ treatment, we examined the expression of the catalase gene *CAT* in patients and WT iNSCs. Interestingly, we observed a noteworthy increase in the expression levels of *CAT* exclusively in SCN1A^mild^ cells (Fig. 1c). The superoxide dismutase (SODs) family plays a crucial role in regulating the levels of various ROS species, thus limiting their potential toxicity^32,33^. Here, we evaluated the expression of SODs upon exposure to 2 mM H_2_O_2_. Interestingly, we observed a significant up-regulation of *SOD1* and *SOD3* in SCN1A^severe^-iNSCs compared to healthy control after 8 and 16 h of treatment, while SCN1A^mild^-iNSCs exhibited a trend similar to that of the WT (Fig. 1d) In addition to SODs, another important family involved in the reduction of free hydrogen peroxide to water is glutathione peroxidase (GPX)^34^. *GPX1* and *GPX3* expression was increased in mutated iNSCs after H_2_O_2_ treatment compared to healthy iNSCs. The up-regulation of these genes in SCN1A^severe^ iNSCs with respect to the other cell lines was particularly significant after 16 h of H_2_O_2_ exposure (Fig. 1e). Mitochondria function as the primary site for ROS production under physiological condition^35^. However, during oxidative stress, mitochondria may undergo damage, triggering the activation of mitophagy to eliminate dysfunctional mitochondria and mitigate cellular ROS level^36^. To assess the impact of oxidative stress on iNSCs mitochondria, we examined their pattern under H2O2 treatment (Fig. 1f). Remarkably, in iNSCs derived from the patient with the severe phenotype, the average size of mitochondrial puncta exhibited a significant increased compared to the other cell lines under H_2_O_2_ treatment (Fig. 1g). The peak increase occurred at 8 h of treatment, followed by a subsequent decrease (Fig. 1g). These findings suggest a potential impairment in cellular clearance pathways in SCN1A^severe ^iNSCs, leading to the accumulation of damaged mitochondria^37^. The heightened production of ROS, coupled with the substantial up-regulation of antioxidant genes in response to the stress stimulus, and the accumulation of mitochondria under H_2_O_2_ treatments, collectively indicate that iNSCs derived from SCN1A^severe^ patients exhibit a heightened susceptibility to oxidative stress.

### NRF2 is hyperactive in SCN1A^severe^ iNSCs under oxidative stress conditions

**Figure 2:**
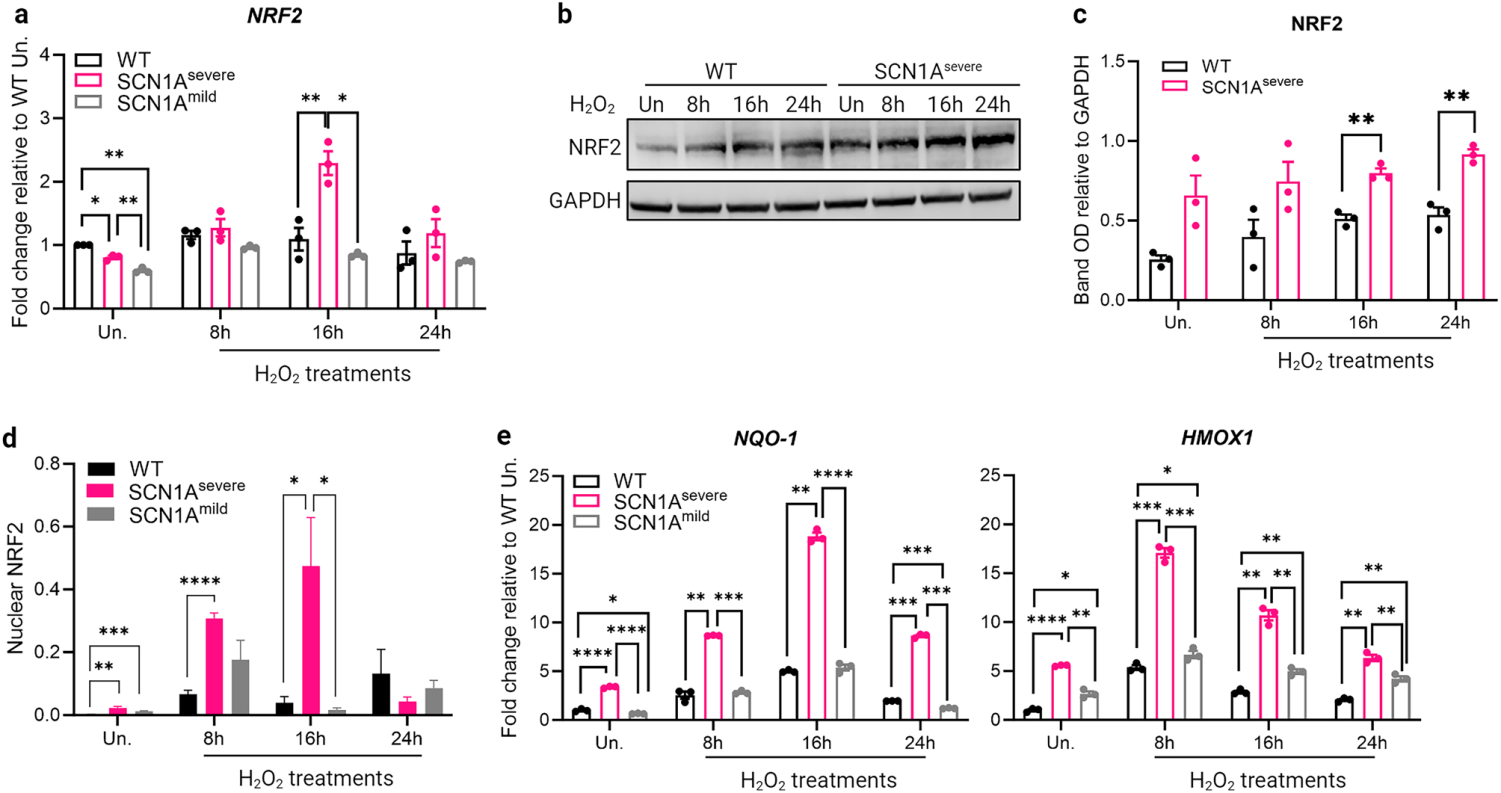
NRF2 expression is upregulated in SCN1A^severe^ patient iNSCs after H_2_O_2_ treatment. (**a**) The expression of *NRF2* gene resulted significantly up-regulated in SCN1A^severe^ patient iNSCs in respect to WT and SCN1A^mild^ cells at 16 h of 2 mM H_2_O_2_ treatments. (**b**) Immunoblot analysis showed that the NRF2 protein increased after 8, 16 and 24 h of H_2_O_2_ treatment in both WT and SCN1A^severe^ patient iNSCs, but for each time point tested we observed a higher expression of NRF2 in patient cells compared to WT cells. (**c**) Bands optical density (OD) of western blot showed in (**b**), calculated as NRF2 bands OD normalized on GAPDH band OD on the same time point. Data are presented as mean ± SEM of three biological replicates. (**d**) 8 and 16 h of H_2_O_2_ treatment induced a prominent NRF2 nuclear translocation in patient iNSCs in respect to SCN1A^mild^ and healthy cells, as shown by quantification of NRF2 immunofluorescence signal overlapping with the nucleus (representative images are shown in Supplementary Fig. S2, at least five image per condition were analysed); *p < 0.05, **p < 0.01, ***p < 0.001, ****p < 0.0001, t-test. (**e**) Expression of NRF2 target genes *NQO1* and *HMOX1* after 8, 16 and 24 h of H_2_O_2_ treatment in WT and patients iNSCs. For qPCR, *GAPDH* was detected as loading control and data are presented as mean ± SEM of three biological replicates. *p < 0.05, **p < 0.01, ***p < 0.001, ****p < 0.0001, t-test.

Nuclear factor erythroid 2-related factor 2 (NRF2) is a key regulator of the oxidative stress response and serves as an oxidative stress sensor and its synthesis is regulated by multiple factors^38,39^. In NSCs, NRF2 has been shown to play a critical role in ensuring cell survival, promoting neurogenesis, and differentiation by regulating intracellular ROS levels^40,41^. The expression of *NRF2* mRNA after H_2_O_2_ exposure was up-regulated in SCN1A^severe^-iNSCs compared to the control and SCN1A^mild^ iNSCs, with statistically significant values obtained after 16 h of treatment (Fig. 2a). Furthermore, immunoblot analysis confirmed a higher expression of NRF2 as well as at the protein level in severely mutated iNSCs compared to the WT cells at all tested time points (Fig. 2b), with significant up-regulation after 16 and 24 h of treatment (Fig. 2c). ROS accumulation promotes the dissociation of NRF2 from its suppressor KEAP-1 and subsequent translocation to the nucleus, where NRF2 binds to the antioxidant/electrophile responsive element (ARE/EpRE) to activate target gene transcription^42^. To investigate the cellular localization of NRF2 following H_2_O_2_ treatment, we performed immunofluorescence analysis (Supplementary fig. S2). Quantification of nuclear NRF2 revealed a significantly higher accumulation of the protein in SCN1A^severe^-iNSCs nuclei than in SCN1A^mild^ and healthy iNSCs, both after 8 and 16 h of H_2_O_2_ exposure (Fig. 2d). The increased activity of NRF2 was further demonstrated by the up-regulation of the NRF2 target genes *NQO1* and *HMOX1* in SCN1A^severe^ iNSCs after H_2_O_2_ treatment (Fig. 2e). Collectively, these data suggest increased activation of NRF2 in response to oxidative stress in SCN1A^severe^ mutated iNSCs.

### Apoptosis is increased in SCN1A^severe^ iNSCs under oxidative stress conditions

**Figure 3:**
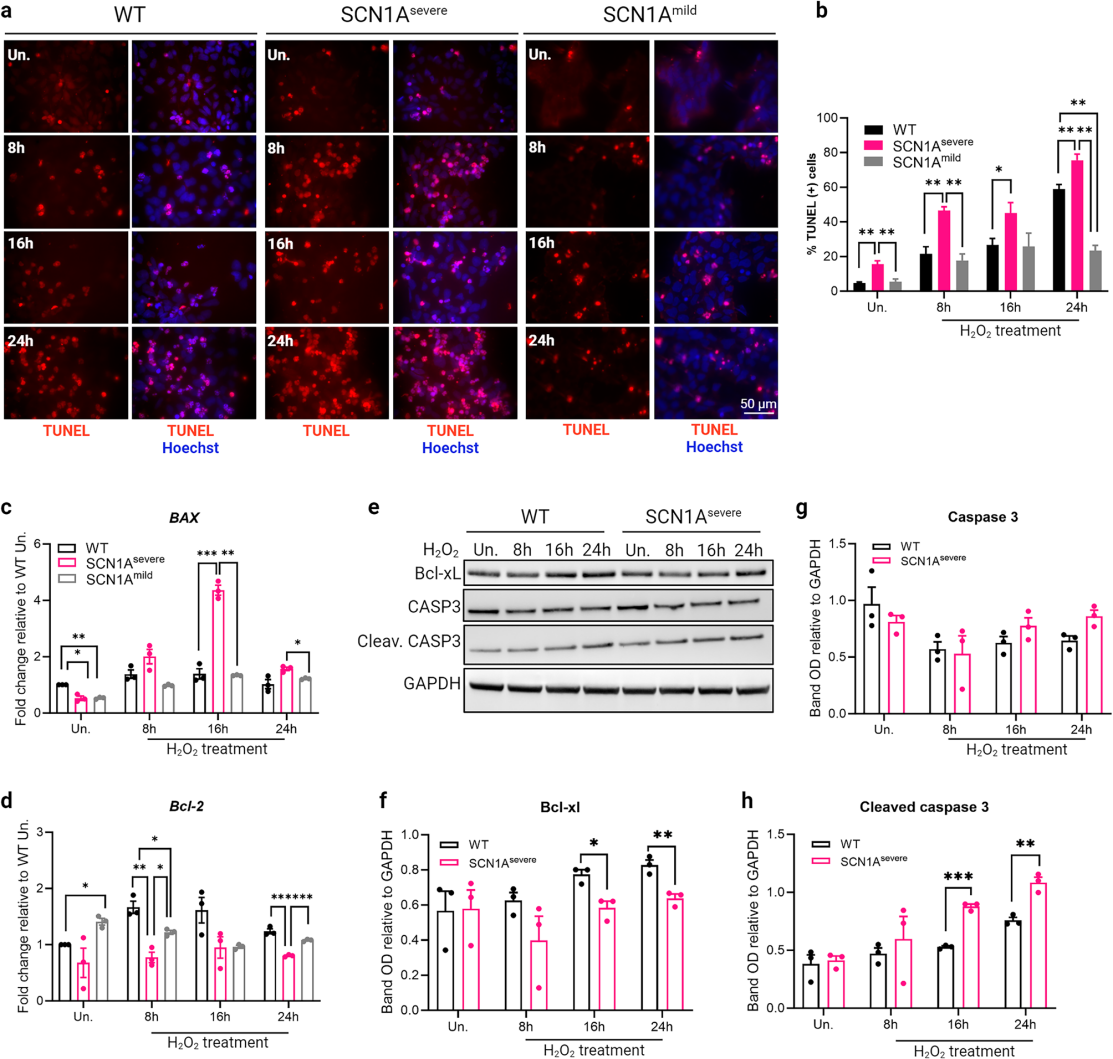
SCN1A^severe^ iNSCs show increased apoptosis compared to SCN1A^mild^ and healthy cells when treated with H_2_O_2_. (**a**) The apoptosis rate of iNSCs was measured using TUNEL assay after treatment with 2 mM H_2_O_2_ for 8, 16 and 24 h. The captured images showed apoptotic cells as bright red fluorescent cells. (**b**) Percentage of TUNEL positive cells calculated for each time point tested. TUNEL analysis showed that SCN1A^severe^ iNSCs treated with H_2_O_2_ displayed a significantly higher number of apoptotic cells compared to SCN1A^mild^ and WT. At least 250 nuclei were analyzed for each condition.*p < 0.05, **p < 0.01, t-test. (**c**) After H_2_O_2_ treatment, the expression of pro-apoptotic gene *BAX* resulted significantly up-regulated in SCN1A^severe^ patient iNSCs compared to SCN1A^mild^ and WT, while (**d**) the anti-apoptotic gene *Bcl-2* exhibited an opposite trend, showing a down-regulation in SCN1A^severe^ iNSCs. For (**c**) and (**d**) panels, *GAPDH* was used as a housekeeping gene and *p < 0.05, **p < 0.01, ***p < 0.001, t-test. (**e**) Patient iNSCs exhibited an increased activation of caspase 3 by cleavage and reduced expression of the anti-apoptotic protein Bcl-xL after H_2_O_2_ treatments in all tested time points compared to WT cells. GAPDH was used as loading control. (**f**–**h**) Quantification of protein band OD of western blot showed in (**e**), *p < 0.05, **p < 0.01, ***p < 0.001, t-test.

Activation of the intrinsic apoptotic pathway is a well-known consequence of oxidative stress, mediated by ROS accumulation^43^. First, we evaluated oxidative stress-induced apoptosis using a TUNEL assay after exposing iNSCs to H_2_O_2_ for 8, 16 and 24 h (Fig. 3a). Remarkably, we detected a significantly higher percentage of TUNEL-positive cells in SCN1A^severe^-derived iNSCs than in SCN1A^mild^ and control iNSCs, both under basal conditions and upon H_2_O_2_ treatment, at all time-points tested (Fig. 3b). The intrinsic apoptotic pathway is regulated by the Bcl-2 family members Bcl-2 and Bcl-xL, which are inhibitors of the pro-apoptotic proteins Bax and Bak^44^. During oxidative stress, activation of Bax leads to permeabilization of the outer mitochondrial membrane and translocation of cytochrome C to the cytoplasm, where it recruits Apaf-1 to initiate apoptosome formation^45^. To investigate the expression of these antagonists, we assessed the expression of the *BAX* gene and found a significant increase after 16 h of treatment with H_2_O_2_ in SCN1A^severe^ iNSCs (Fig. 3c) compared to WT and SCN1A^mild^ cells. Conversely, the expression of the antiapoptotic Bcl-2 gene was notably down-regulated in severely diseased cells under H_2_O_2_ treatments compared to the other cell lines. This down-regulation was particularly significant after 8 and 24 h of treatment with H_2_O_2_ (Fig. 3dD). Moreover, immunoblot analysis unveiled significantly lower levels of the antiapoptotic protein Bcl-xL in SCN1A^severe^ iNSCs compared to WT cells (Fig. 3e,f). Concurrently, at the same time points, the expression of caspase 3 was increased (Fig. 3e,g), with notably higher values for the cleaved caspase 3 fragment at 16 and 24 h of treatment (Fig. 3e,h). Altogheter, these findings provide evidence suggesting that SCN1A^severe^ iNSCs are more sensitive to apoptotic processes under oxidative stress stimuli than SCN1A^mild^ and healthy cells.

### Oxidative stress-induced autophagy is impaired in SCN1A^severe^-iNSCs

**Figure 4:**
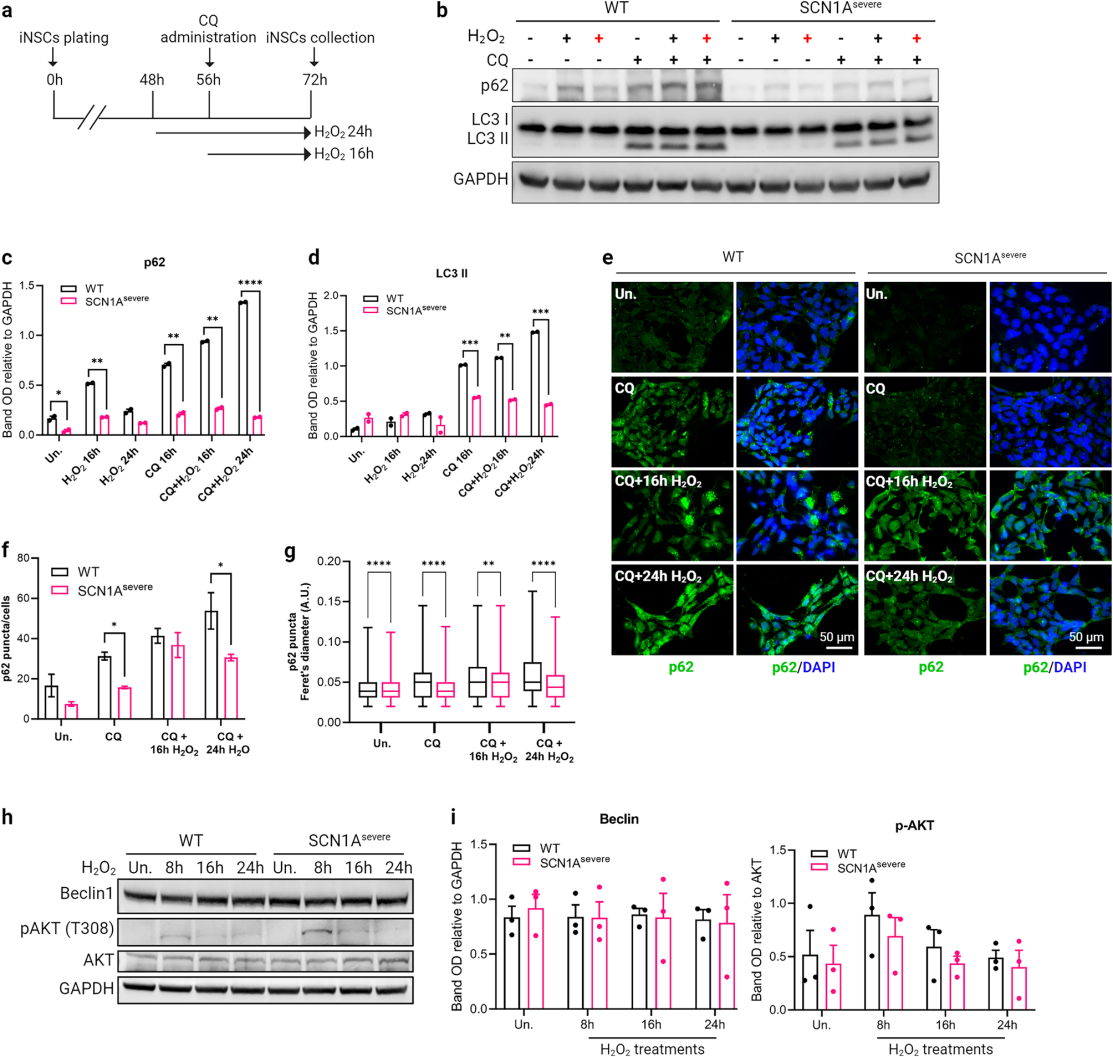
Oxidative stress-induced autophagy is impaired in SCN1A^severe ^patient iNSCs. (**a**) Schematic representation of H_2_O_2_ and CQ treatments on iNSCs. (**b**) Western blot analysis of protein involved in the autophagy process in cells treated with 2 mM H_2_O_2_ alone for 16 (black cross) and 24 h (red cross) or in combination with the autophagy inhibitor chloroquine (CQ). (**c**, **d**) Quantification of P62 and LC3II bands OD demonstrated that autophagy proteins accumulated in iNSCs of both SCN1A^severe^ patient and healthy subject when cells were simultaneously treated with H_2_O_2_ and CQ, but the expression of both proteins remained significantly lower in patient’s cells compared to WT in all time-point tested. *p < 0.05, **p < 0.01, ***p < 0.001, ****p < 0.0001, t-test. (**e**) The lowered expression of P62 protein in SCN1A^severe^ patient cells in respect to WT under H_2_O_2_ and CQ treatment was further confirmed by immunofluorescence analysis. (**f**) Quantification of p62 *puncta* and (**g**) of p62 *puncta* diameter in cells treated with H_2_O_2_ and CQ. (**h**) Western blot analysis of Beclin1 and of its inhibitor AKT under H_2_O_2_ treatment. (**i**) Quantification of western blot bands presented in (**h**) showed that there are no significant differences among the two cell lines in the activation of autophagy pathway under H_2_O_2_ treatment.

Autophagy is a recycling process that plays a crucial role in maintaining cellular homeostasis through the degradation of pre-existing intracellular components to build new ones^46^. Autophagy not only governs the maintenance of adult NSCs and activation of quiescent NSCs, but also influences neurogenesis by promoting the maturation and survival of newborn neurons^47,48^. Oxidative stress triggers autophagy together with the antioxidant response to reduce the levels of ROS and restore cellular homeostasis^49,50^. Considering this, our objective was to evaluate the activation of autophagy in response to oxidative stress in both healthy and diseased iNSCs. Before delving into the analysis of autophagy in iNSCs, we ensured their synchronicity, as the degree of activation of autophagic flux can vary depending on cell cycle phases in mitotic cells^51^. Examination of the cell cycle (Supplementary Fig. S3a and b) and the determination of the cell population doubling time (Supplementary Fig. S3c) for both healthy and diseased iNSCs, revealed no significant differences among the three cell line. Subsequently, iNSCs were exposed to H_2_O_2_ alone or in combination with the autophagy inhibitor chloroquine (CQ) to evaluate the autophagic flux under oxidative stress condition (Fig. 4a). To determine the optimal conditions for proper autophagy inhibition, WT iNSCs were treated with CQ at 50 μM and 100 μM at different times. The accumulation of LC3-II was used as a means of autophagy inhibition, and its maximum accumulation was observed after 16 h of 100 μM CQ treatment (Supplementary Fig. S3d). Next, we investigated the impact of H_2_O_2_ on autophagy flux by analysing autophagy-related proteins using immunoblotting. After 16 h of H_2_O_2_ treatment, we detected an increase in the p62 expression level in both SCN1A^severe^ and control iNSCs with respect to the untreated condition; when the exposure to H_2_O_2_ was prolonged to 24 h, the p62 levels decreased, suggesting that H_2_O_2_ activates autophagy flux in iNSCs (Fig. 4b,c). These data were confirmed by the accumulation of p62 and LC3-II when cells were simultaneously treated with H_2_O_2_ and CQ (Fig. 4b–d). Although diseased and control iNSCs shared a similar trend in the expression of autophagic proteins under treatments, SCN1A^severe^ iNSCs showed a lower expression of LC3-II and particularly of p62 at all the time points tested (Fig. 4b–d). We performed a similar experiment comparing WT and SCN1A^mild^ iNSCs, and significant differences were not observed between these two lines, except for the H_2_O_2_ 16-h treatment (Supplementary Fig. S3e and f). The data relative to p62 in WT and SCN1A^severe^ cells were further confirmed by immunostaining in the same treatment conditions (Fig. 4e). Specifically, we observed a reduced number of p62 *puncta* per cell (Fig. 4f) and with a smaller diameter (Fig. 4g) in diseased iNSCs. Next, we asked whether the impairment of autophagy in SCN1A^severe^ iNSCs was associated with a dysregulation of the upstream signalling pathways. Thus, we examined the protein expression levels of canonical autophagy regulators, such as Beclin-1, an autophagy activator, and AKT, an autophagy inhibitor. Under 16 and 24 h of H_2_O_2_ treatment, no differences were detected in the expression of these two proteins between SCN1A^severe^ and WT cell lines (Fig. 4h,i), suggesting that the lower autophagy flux observed in mutated iNSCs is not due to variations in the activation of the pathway, but could be likely attributed to a less efficient formation of autophagosomes. Supporting this hypothesis, we observed reduced levels of WIPI2 *puncta* in both untreated and H_2_O_2_ treatment conditions in mutated iNSCs compared to healthy control (Supplementary Fig. S3g and h). To assess whether the differences observed between the three cell lines under H_2_O_2_ treatment were attributable to inter-individual variation, we treated both WT and diseased iNSCs with 100 μM CQ for 4, 8, 16 and 24 h (Supplementary Fig. S3i) and measured basal autophagic flux for each cell line by the calculation of the ΔLC3-II as indicated in the formula (2) ^52^, demonstrating the absence of significant differences in the basal autophagic flux among the three cell lines (Supplementary Fig. S3j). Collectively, these data demonstrated a deceleration of the autophagic process in SCN1A^severe^ iNSCs under H_2_O_2_ treatment.

### HSBP1, a p38 downstream target, is down-regulated in SCN1A^severe^ iNSCs

**Figure 5:**
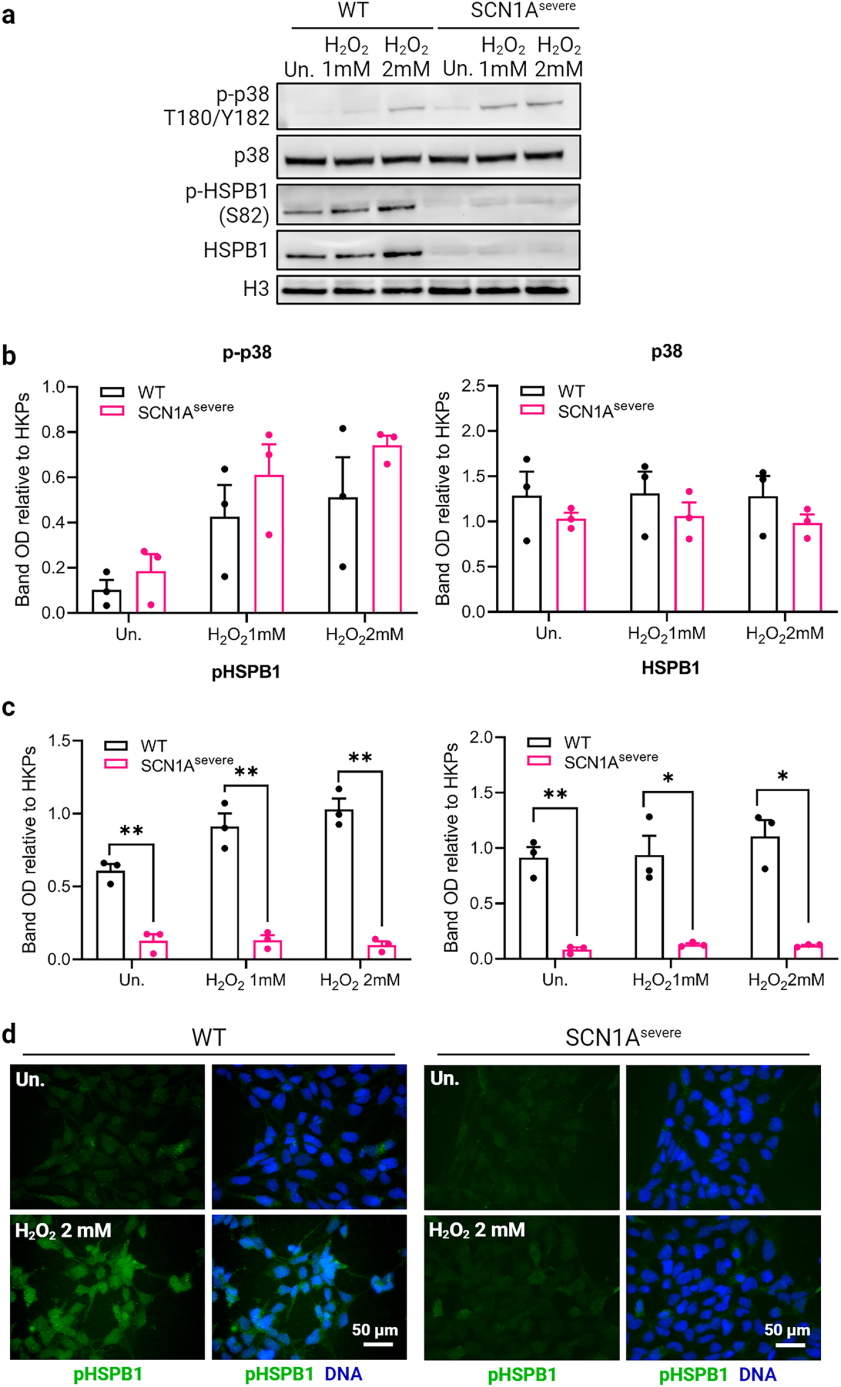
Down-regulation of HSBP1, a p38 downstream target, in SCN1A^severe^ iNSCs. (**a**) Western blot analysis of WT and SCN1A^severe^ iNSCs treated for 30 min with 1 or 2 mM H_2_O_2_ showed an activation of the stress-related kinase p38 by its phosphorylation at residues 180 and 182 in both cell lines tested. A higher activation of p38 in patient cells compared to WT was observed after H_2_O_2_ treatment. In the same blot, we detected the expression of the p38 target HSPB1. Interestingly, we found that this protein was expressed at very low levels in SCN1A^severe^ patient iNSCs compared to WT, in both total and phosphorylated form. GAPDH was used as loading control. (**b**, **c**) Quantification of biological replicates of western blot shown in (**a**). (**d**) pHSPB1 downregulation in SCN1A^severe^ cells under H2O2 treatments was further confirmed by immunofluorescence analysis.

Among the mitogen-activated protein kinases (MAPKs), p38 has a pivotal role in the cellular response to stress stimuli^53^. Notably, p38 is also involved in the regulation of apoptosis and autophagy induced by stress through its downstream target, heat shock protein beta 1 (HSPB1)^54–56^. HSPB1, also known as heat shock protein 27 (HSP27), is a member of the small molecular weight heat shock protein (HSP) family, which is involved in various cellular protective mechanisms, including the reduction of ROS levels^57,58^. Various triggering factors (heat shock, oxidative stress, inflammation) can induce the activation of HSPB1 by its phosphorylation^59^. Given the substantial accumulation of ROS, predisposition to apoptosis and defective autophagy in SCN1A^severe^ iNSCs under oxidative stress, we aimed to investigate the functionality and activation of HSPB1 under the same experimental conditions. We observed a higher phosphorylation of HSPB1 in iNSCs after 30 min of exposure to H_2_O_2_ and this phosphorylation level positively correlated with the concentration of H_2_O_2_ used (Supplementary Fig. S4a and b). Under the same conditions, we observed the phosphorylation of p38 at residues T180 and Y182 (Supplementary Fig. S4a and b). To confirm the correlation between p38 and HSPB1 activation, we treated iNSCs with 1 mM H_2_O_2_ or 2 mM H_2_O_2_ alone, or in combination with the p38 inhibitor SB203580 for 30 min. Remarkably, we found that the phosphorylation levels of both p38 and HSPB1 decreased when cells were co-treated with H_2_O_2_ and SB203580 compared to H_2_O_2_ treatment alone (Supplementary Fig. S4c and d). These data confirmed that in iNSCs, the phosphorylation of HSPB1 under oxidative stress conditions is dependent on p38 activation. The phosphorylation of the two proteins is maintained also under H_2_O_2_ treatment of 8, 16 and 24 h (Supplementary Fig. S4e and f). To validate the functionality of the p38-HSPB1 axis in diseased cells, we treated both healthy and SCN1A^severe^ iNSCs with H_2_O_2_ and assessed the presence of both proteins by immunoblotting. In mutated iNSCs, the phosphorylation of p38 was higher than that observed in control iNSCs after a 30 min treatment with 1 mM H_2_O_2_ or 2 mM H_2_O_2_ (Fig. 5a), although this up-regulation was not significant (Fig. 5b). However, we found reduced activation of HSPB1 in patient iNSCs at the same time point, as shown by the significantly decreased expression of the phosphorylated form of HSPB1 (Fig. 5a,c). Western blot analysis also revealed a drastic down-regulation of the total form of HSPB1 in patient cells compared to WT cells, even under untreated conditions (Fig. 5a,c). These findings were further confirmed by immunofluorescence analysis (Fig. 5d). The expression of HSPB1 protein in SCN1A^mild^ iNSCs is slightly higher than in WT cells (Supplementary Fig. S5a), even though this up-regulation resulted not significant (Supplementary Fig. S5b). These data indicate that the down regulation of HSPB1 protein is a specific trait of the patient with the severe phenotype. For a comprehensive analysis, we examined the mRNA expression of *HSPB1* gene and other HSP-related genes, such as *HSPA1A*, *HSPA1B*, *DNAJA1*, *HSP90AA1* in WT, SCN1A^severe^ and SCN1A^mild^ iNSCs. Our data indicated up-regulation of HSPs genes in patients compared to the healthy control, except for *HSPB1*. Consistent with the protein trend, *HSPB1* is significantly down regulated in SCN1A^severe^ iNSCs compared to the other two cell lines. Additionally, *DNAJA1* exhibited similar expression levels between SCN1A^mild^ and WT iNSCs (Supplementary Fig. S5c). Furthermore, we analyzed the protein expression of another member of the HSPs, HSPA8, in WT, SCN1A^severe^ and SCN1A^mild^ iNSCs. Our results revealed a lack of significant differences between the three cell lines (Supplementary Fig. S5d and e). These results suggested that the down-regulation specifically affects HSPB1 in SCN1A^severe^ iNSCs, implying a potential pivotal role for this specific protein in the pathogenesis of the disease under oxidative stress condition.

### Treatment with ascorbic acid leads to the restoration of HSPB1 and p62 levels and counteracts the effect of H_2_O_2_ treatment in SCN1A^severe^ iNSCs

**Figure 6:**
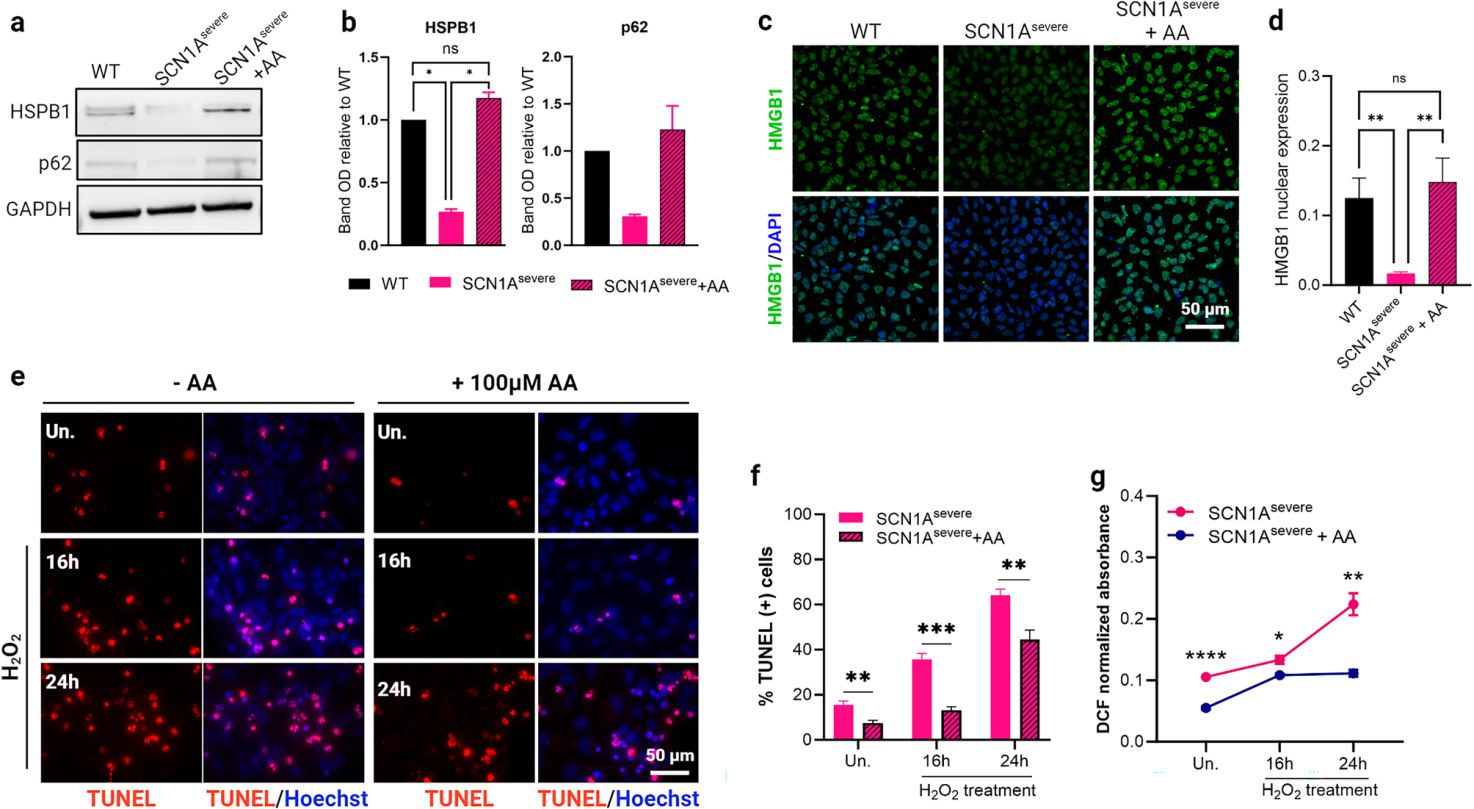
Administration of ascorbic acid (AA) resulted in the restoration of HSPB1 and p62 levels and nuclear translocation of HMGB1, countering the effects of H_2_O_2_ in SCN1A^severe^ iNSCs. (**a**) The treatment of SCN1A^severe^ iNSCs with 100 μM AA for 12-h duration increase HSPB1 and p62 expression levels comparable to those observed in the WT cells. (**b**) Quantification of western blot bands shown in (**a**) presented as mean ± SEM of two biological replicates. *p < 0.05, t-test. (**c**, **d**) Treating SCN1A^severe^ cells with the antioxidant molecules AA led to nuclear translocation of HMGB1, a transcription factors interacting protein that regulates HSPB1 and p62 expression. **p < 0.01, t-test. (**e**) TUNEL assay in SCN1A^severe^ iNSCs treated with 2 mM H_2_O_2_ for 16 or 24 h showed a reduction of apoptotic cells (red fluorescence) in presence of AA pre-treatment (100 μM for 12 h) with respect to untreated condition. (**f**) Quantification of the number of TUNEL positive cells in experiment presented in (**e**); at least 300 nuclei were analysed for each condition. **p < 0.01, ***p < 0.001, t-test has been calculated vs. untreated condition in the same time point. (**g**) Production of ROS due to H_2_O_2_ treatment in diseased iNSCs in presence or absence of AA pre-treatment. AA significantly lowered levels of ROS across all tested time intervals. The DCFA-DA signal of each well was normalised to the number of cells in the same well. Data are presented as mean ± SEM of three biological replicates, *p < 0.05, **p < 0.01, ****p < 0.0001, t-test has been calculated vs. untreated in the same time point.

To find a potential strategy to restore HSPB1 expression and mitigate oxidative stress in iNSCs of the patient, we subjected the cells to various concentrations of the extensively investigated antioxidant compound ascorbic acid (AA). Our observation revealed that the impact of AA treatment on HSPB1 expression followed a dose- and time-dependent pattern. Specifically, a significant accumulation of HSPB1 was observed when the cells were treated with 100 and 200 μM AA for 12 h, while higher concentrations (400 μM) or longer treatments (24 h) did not elicit any discernible effect on HSPB1 expression (Supplementary Fig. S6). Considering the role of HSPB1 in the formation of p62 bodies^30^, we assessed the expression of this protein under the same AA treatment, and we found a similar trend to that observed for HSPB1 (Supplementary Fig. S6). Interestingly, the treatment of SCN1A^severe^ iNSCs with 100 μM AA for 12 h resulted in the expression of HSPB1 and p62 protein at the levels comparable to those observed in the WT iNSCs (Fig. 6a,b). To investigate a potential mechanism responsible for the restoration of HSPB1 and p62 expression in the presence of AA, we found in the literature that high mobility group-box 1 (HMGB1) plays a specific role in promoting the expression of HSBP1 in cardiomyocytes^60^ and of p62 in cancer cells^61^. Considering that HMGB1 is known to be a target of antioxidant compounds^62,63^, we conducted immunocytochemistry to investigate the expression and subcellular localization of HMGB1 in SCN1A^severe^ iNSCs treated with 100 μM AA for 12 h in comparison to untreated SCN1A^severe^ and WT cells. Our experiment demonstrated that AA administration yielded nuclear translocation of HMGB1 in SCN1A^severe^ iNSCs, as shown by the significant augmentation of HMGB1 signal overlap with nuclei under AA treatment (Fig. 6c). Furthermore, the cellular localization of HMGB1 in SCN1A^severe^ iNSCs treated with AA exhibited levels comparable to those observed in WT cells (Fig. 6d). Collectively, these data demonstrated the influence of AA on the expression and cellular localization of critical stress proteins in SCN1A^severe^ iNSCs.

Next, we evaluated the impact of AA treatment in SCN1A^severe^ iNSCs subjected to 16 and 24 h of H_2_O_2_ exposure. To do so, we pre-treated the cells with 100 μM AA for a duration of 12 h, followed by the introduction of H_2_O_2_ for 16 and 24 h. We observed a minor activation of the H_2_O_2_-related apoptotic process in the presence of AA treatment, as evidenced by a decreased number of cells exhibiting positivity in the TUNEL assay (Fig. 6e,f). Furthermore, AA administration resulted in a significant reduction in ROS production with respect to the untreated condition (Fig. 6g). These findings suggest that the use of antioxidant compounds, such as AA, may represent a potential approach to protect iNSCs from oxidative stress-derived damage.

## Discussion

FS are common convulsive disorders, primarily affecting children^64^. Neuronal hyperactivity during FS can have a negative impact on NSCs residing in the hippocampus, representing a possible link between early-life seizures and the subsequent development of MTLE^6,7^. However, the precise underlying molecular mechanisms connecting FS and MTLE remain elusive. Considering that FS are associated with an increase in the production of ROS^15^, we hypothesised that oxidative stress can damage human NSCs, representing a potential mechanism for the subsequent pathogenesis of MTLE. Our experimental model consisted of iNSCs obtained from a patient with FS, drug-resistant MTLE and hippocampal sclerosis. For a comprehensive analysis, we compared the results obtained from the iNSCs of this patient with those of a sibling affected by a milder phenotype and a healthy control. After treatment with H_2_O_2_, ROS massively accumulate in diseased iNSCs, with a severe increase after prolonged exposure. Surprisingly, we found that key antioxidant genes and proteins were up-regulated in severely diseased iNSCs compared to healthy cells, but these cells appear to be unable to effectively counteract and reduce ROS production, resulting in enhanced apoptosis. These findings prompted us to investigate concomitant pathways that contribute to the antioxidant response. Autophagy is one of the defensive mechanisms that cells activate in the presence of ROS^65,66^. In our experiments, we observed less efficient autophagic flux under oxidative stress conditions in SCN1A^severe^ iNSCs than in control cells, as shown by the reduced accumulation of the autophagy markers p62 and LC3 I-II when autophagic flux was blocked. Impaired autophagic flux can alter protein homeostasis and affect NSCs functionality and survival, accelerating NSCs ageing^67^. Protective mechanisms fighting oxidative stress are finely regulated by mitogen-activated protein kinase (MAPK) p38, also known as stress-activated protein kinases (SAPKs)^53,68^. In particular, in the presence of excessive ROS, p38 is involved in the remodelling of actin filaments through phosphorylation of its downstream target HSPB1^69^. Although the activation of p38 by phosphorylation after H_2_O_2_ treatment was similar in SCN1A^severe^ and WT cell lines, we detected very low levels of HSBP1 protein, both in the total and in the phosphorylated form in the diseased iNSCs. HSPB1, also known as HSP27, was identified as a chaperone that acts in the refolding of damaged proteins following heat shock; further studies revealed that the protein is critically involved in the oxidative stress response^59,70^. Among other antioxidant functions, HSPB1 leads to an increase in the intracellular levels of glutathione (GSH)^71^, which is used by GPXs to reduce H_2_O_2_ or organic hydrogen peroxide into water and the corresponding alcohols^34^. We hypothesised that the down-regulation of HSPB1 observed in mutated iNSCs may decrease GSH levels available for GPXs to catalyse the reduction of H_2_O_2_, leading to an accumulation of ROS despite the up-regulation of *GPX* gene in presence of oxidative stress. Moreover, HSPB1 is known to play a role in regulating autophagy and apoptosis in various cell types, including NSCs^54,72,73^. Specifically, HSPB1 promotes autophagy and prevents apoptosis^74–76^. Mutations in HSPB1 have been shown to impair autophagic flux by disrupting p62 bodies^30^. A recent study demonstrated that HSPB1 regulates the formation of p62 condensates on damaged lysosomes, thereby facilitating autophagosome formation^27^. Hence, the decreased autophagy flux and heightened apoptosis susceptibility observed in mutated iNSCs could be attributed to the down-regulation of HSPB1.

To find a potential mechanism for mitigating oxidative stress in severely diseased-iNSCs, we treated the cells with the antioxidant compound AA (vitamin C) prior to the administration of H_2_O_2_. Previous studies have already demonstrated the protective effects of AA in reducing neuronal damage when administered before the induction of seizures in rats^77,78^. In our model, pre-treatment of diseased iNSCs with AA in the presence of oxidative stress led to an up-regulation of proteins associated with cellular protection, such as HSPB1 and p62. These proteins collaborate to eliminate damaged organelles and proteins from the cell, thereby maintaining cellular homeostasis^30^ even under stress conditions^27^. More interestingly, in SCN1A^severe^ iNSCs, treatment with AA resulted in a reduction in ROS production and a decrease in apoptotic events caused by H_2_O_2_. In summary, our findings suggest that dysfunction in pathways related to the response to oxidative stress in patients who have experienced FS can lead to damage in iNSCs. These impaired NSCs may subsequently contribute to aberrant neurogenesis, potentially contributing to the development of MTLE. The administration of antioxidant compounds to patients experiencing FS could potentially serve as a preventive measure against the subsequent development of MTLE.

## Methods

### Patient, cell culture and generation of iPSCs-derived neural stem cells (iNSCs)

The study was conducted following the guidelines of the Declaration of Helsinki, and approved by the Ethics Committee of the “Magna Graecia” University of Catanzaro and the Azienda Ospedaliero—Universitaria “Mater Domini” (Approval number: AOM92_2020). Informed consent was obtained from all subjects involved in the study.

The diseased iPSCs lines used in this study (named SCN1A^severe^ and SCN1A^mild^)belong to the patients referred as subjects IV-3 and IV-4, respectively, in the pedigree reported in previous works^25,26^. The generation and characterization of these lines was reported in^79^ and the clinical features of the SCN1A^severe^ patient are described in^80^. Briefly, both patients carry an inherited missense mutation in the *SCN1A *gene and experienced FS during childhood. However, only the patient identified as SCN1A^severe^ also developed MTLE, indicating that the mutation alone is not sufficient to produce a more severe pathological phenotype. As a control, we used the line hiPSCs-3, described in^81^, herein referred to as healthy or wild type (WT). iPSCs were cultured in 60-mm dishes coated with Matrigel (Corning, Corning, NY, USA) in mTeSR1 medium (StemCell Technologies, Vancouver, BC, Canada), in a humidified incubator at 37 °C with 5% CO_2_. At 80% confluence, cells were induced to differentiate into NSCs using Gibco® PSC Neural Induction Medium (Thermo Fisher Scientific, Waltham, MA, USA), according to the manufacturer’s instructions. When iNSCs reached confluency, they were dislodged using Gibco™ StemPro™ Accutase™ Cell Dissociation Reagent (Thermo Fisher Scientific, Waltham, MA, USA) and plated at a density of 1 × 10^5^ cells per cm^2^ on Geltrex™ LDEV-Free hESC-qualified Reduced Growth Factor Basement Membrane Matrix (Thermo Fisher Scientific, Waltham, MA, USA) coated dishes for expansion.

### Reagents, drugs and in vitro treatments

Cells were consistently treated 48 h after passage. H_2_O_2_ was directly added to the culture medium at a final concentration of 1 mM or 2 mM for 4, 8, 16 and 24 h or 30 and 60 min in the shorter treatments. For treatment with SB203580 (StemCell Technologies, Vancouver, BC, Canada), a p38 MAPK inhibitor, a final concentration of 10 µM was prepared in the medium, and the cells were exposed to SB203580 for 30 min either alone or in combination with H_2_O_2_. Chloroquine (Sigma Aldrich, St. Louis, MO, USA), utilised to inhibit autophagy, was freshly prepared in water and added to the medium at a final concentration of 100 µM for 16 h, either alone or in combination with H_2_O_2_. For treatment with ascorbic acid (Sigma Aldrich, St. Louis, MO, USA), the molecule was dissolved in water and added directly to the culture medium for 12 or 24 h at final concentrations of 100, 200 and 400 µM.

### ROS detection assay

For ROS detection, 3 × 10^4^ cells were plated in each well of a 96 black/clear bottom plate and treated 48 h after passage. Intracellular ROS levels were assessed using the ROS Detection Assay Kit (OZ Biosciences, Marseille, France) following the manufacturer’s instructions with slight modifications. After treatment, the cells were washed once with 1X PBS and then stained with 10 µM DCF-DA solution for 15 min at 37 °C in the dark. The fluorescence intensity was measured using a GloMax® Multi + Detection System (Promega, Madison, WI, USA) and normalised to the number of cells in the corresponding well.

### RNA extraction and quantitative real-time PCR

Total RNA was extracted with TRIzol Reagent (Thermo Fisher Scientific, Waltham, MA, USA), and 1 µg RNA was reverse transcribed with the High-Capacity cDNA Reverse Transcription Kit (Applied Biosystems, Waltham, MA, USA). The cDNA was amplified through qRT‒PCR, performed by QuantStudio™ 7 Pro Real-Time PCR System (Thermo Fisher Scientific, Waltham, MA, USA) using SensiFAST SYBR Hi-ROX Kit (Meridian Bioscience, Cincinnati, OH, USA). The Ct values for each target gene were normalised to the housekeeping gene glyceraldehyde 3’-phosphate dehydrogenase (*GAPDH*). A list of sequences of primers used in this study is presented in Supplementary Table S1.

### Mitotracker assay

MitoTracker assay was performed using the MitoTracker™ Dyes for Mitochondria Labelling (Thermo Fisher Scientific, Waltham, MA, USA) according to the manufacturer’s instructions. Briefly, the dye was diluted in the cell culture medium at a final concentration of 20 nM and added to the plate after H2O2 treatments for 45 min at 37 °C. Nuclei staining was performed using the Hoechst dye at a final concentration of 1 µg/mL for 30 min at 37 °C. Coverslips were mounted with Dako Fluorescent Mounting Medium (Agilent, Santa Clara, CA, USA) and fluorescence was acquired with Leica Thunder DMi8 using LAS X (v.3.7.4.23463) software. Analysis of immunofluorescence images was performed using ImageJ Fiji software^82^.

## Western blot analysis

Cells were collected in cold 1X PBS without calcium and magnesium (Corning, Corning, NY, USA) and lysed with RIPA Buffer, containing 50 mM Tris–HCl pH 7.5 (Gibco, Waltham, MA, USA), 150 mM sodium chloride (Sigma Aldrich, St. Louis, MO, USA), 1% Triton X-100 (Sigma Aldrich, St. Louis, MO, USA), 0.5% sodium deoxycholate (Sigma-Aldrich, St. Louis, MO, USA), 0.1% SDS (Sigma-Aldrich, St. Louis, MO, USA), completed with Halt™ protease inhibitors and Halt™ phosphatase inhibitors (both from Thermo Fisher Scientific, Waltham, MA, USA) for total protein extraction. A Bradford assay (Bio-Rad, Hercules, CA, USA) was performed to assess the protein concentration. Equal amounts of protein (15 µg) were diluted in 1X Sample Buffer made of Bolt™ LDS Sample Buffer (4X) and Bolt™ Reducing Agent (10X) (both from Thermo Fisher Scientific, Waltham, MA, USA). Following denaturation for 10 min at 70 °C, samples were resolved in Bolt™ 4–12% Bis–Tris Plus gels (Thermo Fisher Scientific, Waltham, MA, USA), using 1X Bolt™ MES SDS Running Buffer 20X (Thermo Fisher Scientific, Waltham, MA, USA), and transferred to nitrocellulose membranes (Bio-Rad, Hercules, CA, USA) by a Trans-Blot Turbo transfer system (Bio-Rad, Hercules, CA, USA). After blocking for 1 h at room temperature with 5% milk (PanReac AppliChem, Darmstadt, Germany) in 1X TBS-0,1% Tween, the membranes were incubated at 4 °C overnight with primary antibodies. See Supplementary Table S2 for the complete list of primary antibodies used in this study. Horseradish peroxidase (HRP)-conjugated secondary antibodies (1:10.000, Jackson ImmunoResearch, West Grove, PA, USA) were incubated for 1 h at room temperature. Protein bands were detected using Clarity™ Western ECL Blotting Substrates (Bio-Rad, Hercules, CA, USA), and images were acquired by Alliance™ Q9-Atom (Uvitec, Cambridge, UK). Analyze Gels tool of Fiji Software was used to quantify western blot bands. Anti-GAPDH, anti-H3 and anti-Υ tubulin antibodies were used as loading controls. All uncropped western blots displayed in this work along with the respective replicates can be found as Supplementary file.

### Immunofluorescence

Cells were fixed with 3.7% (vol/vol) formaldehyde (Sigma Aldrich, St. Louis, MO, USA), blocked for 2 h at room temperature with 10% goat serum (Thermo Fisher Scientific, Waltham, MA, USA) and 0.1% Triton X-100 (Sigma Aldrich, St. Louis, MO, USA) in 1X DPBS with calcium and magnesium (Gibco, Waltham, MA, USA) followed by incubation with primary antibodies at 4 °C overnight (see Supplementary Table S2). AlexaFluor-488 conjugated secondary antibody (1:500, Thermo Fisher Scientific, Waltham, MA, USA) was incubated for 1 h at room temperature. DAPI (4′,6-diamidino-2-phenylindole, Thermo Fisher Scientific) was used to stain nuclei. Coverglasses were mounted with Dako Fluorescent Mounting Medium (Agilent, Santa Clara, CA, USA). Images were acquired with Leica DMi8 inverted microscope or Leica Thunder DMi8 using LAS X (v.3.7.4.23463) software. Analysis of immunofluorescence images was performed using ImageJ Fiji software.

### TUNEL assay

For in situ apoptosis detection, 4 × 10^4^ cells were seeded in 8-well chamber slides and treated with 2 mM H_2_O_2_ alone or after pre-treatment with ascorbic acid, as described in more detail in the corresponding paragraph of the results section. Cells were fixed with 3.7% (vol/vol) formaldehyde (Sigma Aldrich, St. Louis, MO, USA) and processed with the Click-iT™ Plus TUNEL Assay Kit AlexaFluor 594 (Thermo Fisher Scientific, Waltham, MA, USA), following the manufacturer’s protocol. Cover glasses were mounted with Dako Fluorescent Mounting Medium (Agilent, Santa Clara, CA, USA). Images were acquired with a Leica DMi8 inverted microscope, using LAS X (v.3.7.4.23463) software. TUNEL positive cells were manually counted using the multipoint tool of ImageJ Fiji software.

### Cell cycle

For cell cycle assay, cells were dislodged with Accutase, collected in 1X PBS and fixed in 70% ethanol for one hour at 4° C. After fixation, cells were washed two times in 1X PBS and incubated one hour at room temperature in propidium iodide PI solution (0.01% Triton-X 100, 50 µg/mL PI and 100 µg/mL RNAse A in 1X PBS). PI-stained cells were detected by flow cytometry (BD LSRFortessa x-20) and data analysis and graphs *were generated* using *FlowJo* software (Tree Star Inc, Ashland, OR, USA).

### Doubling time calculation

For the analysis of cell growth rate, 1 × 10^5^ cells were plated in a 48-well plate and counted 8 and 72 h after plating to determine initial and final concentration. Doubling time of iNSCs was calculated using the formula:

1 $$Doubling \;time = \frac{Duration \cdot ln\left( 2 \right)}{{ln \left( {\frac{Final \;concentration}{{Initial \;concentration}}} \right)}}$$

### Quantification of basal autophagic flux

Autophagy flux was evaluated through western blot analysis measuring change in LC3-II levels following treatment with the autophagy inhibitor CQ. Quantification was performed using the method reported in Bensalem et al.^52^ applying the formula:

2 $$\Delta \left( {{\text{LC3}} - {\text{II}}:{\text{GAPDH}}} \right) = \left( {{\text{LC3}} - {\text{II}}:{\text{GAPDH with CQ}}} \right){-}\left( {{\text{LC3}} - {\text{II}}:{\text{GAPDH without CQ}}} \right)$$

where LC3-II and GAPDH are the optical density (OD) of western blot bands measured using the tool Analyse Gels of ImageJ Fiji Software.

### Statistical analysis

Statistical analysis was performed using multiple unpaired *t* tests with Welch correction in GraphPad Prism software, version 9.3.1. Data are represented as the means of two or three biological replicates ± SEM. *p* values representation: *p < 0.05, **p < 0.01, ***p < 0.001, ****p < 0.0001.

### Supplementary Information

Supplementary Information.

## Data Availability

The datasets used and/or analyzed during the current study are available from the corresponding author upon reasonable request.
