# Supplementary material for: Ascorbic acid mitigates the impact of oxidative stress in a human model of febrile seizure and mesial temporal lobe epilepsy
Source: Sci Rep. 2024 Mar 11;14:5941. doi: 10.1038/s41598-024-56680-4 (PMC10928078; doi:10.1038/s41598-024-56680-4)

Ascorbic acid mitigates the impact of oxidative stress in a human model of febrile seizure and mesial temporal lobe epilepsy

Authors

Stefania Scalise[1†], Clara Zannino[1†], Valeria Lucchino[1], Michela Lo Conte[1], Vittorio Abbonante[2], Giorgia Lucia Benedetto[3], Mariangela Scalise[1], Antonio Gambardella[3], Elvira Immacolata Parrotta[3*], Giovanni Cuda[1]

SUPPLEMENTARY FIGURES

Supplementary Figure S1

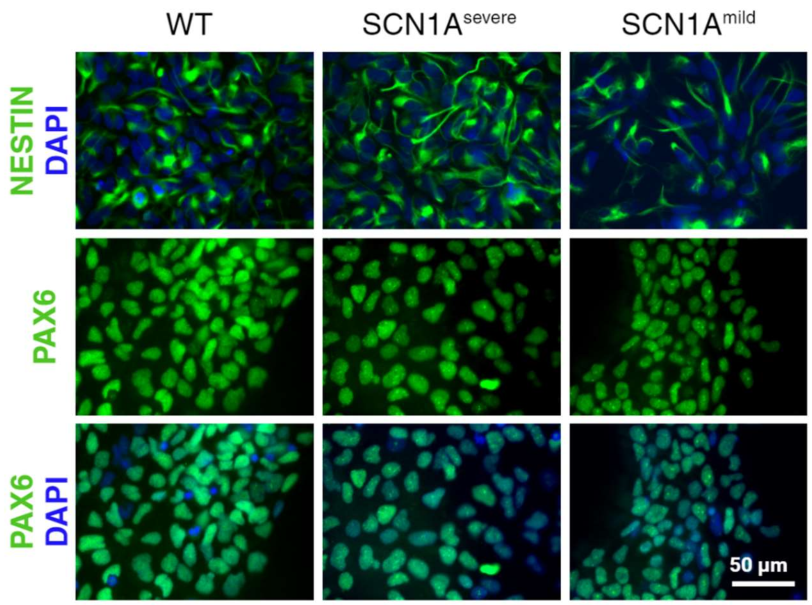

Supplementary Figure S2

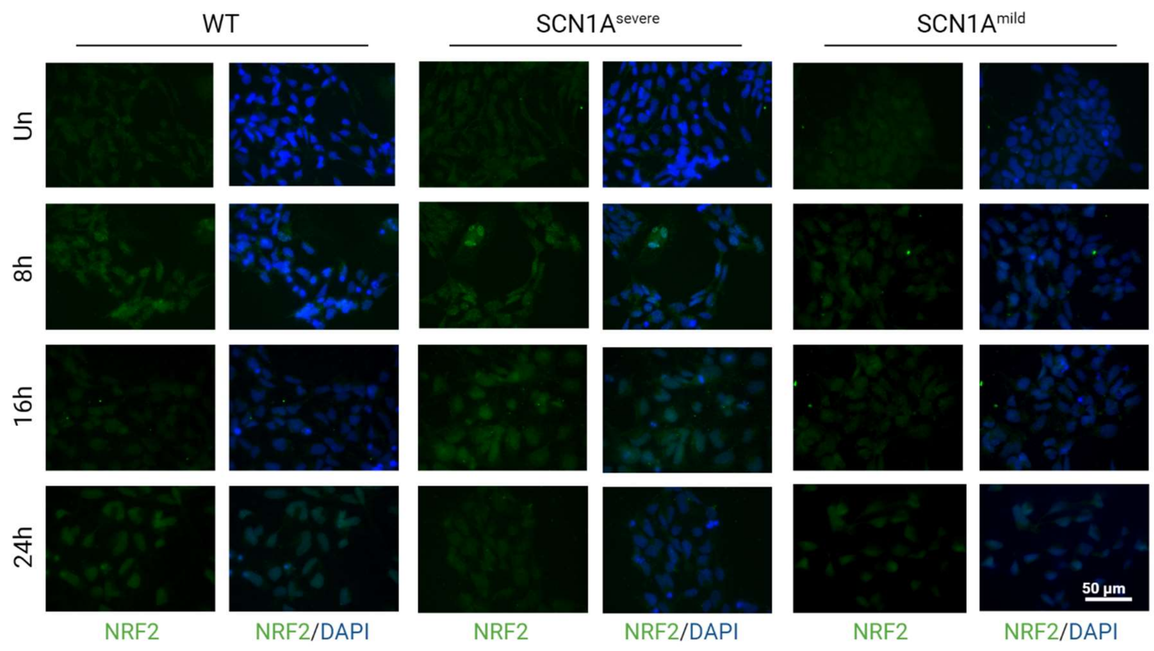

Supplementary Figure S3

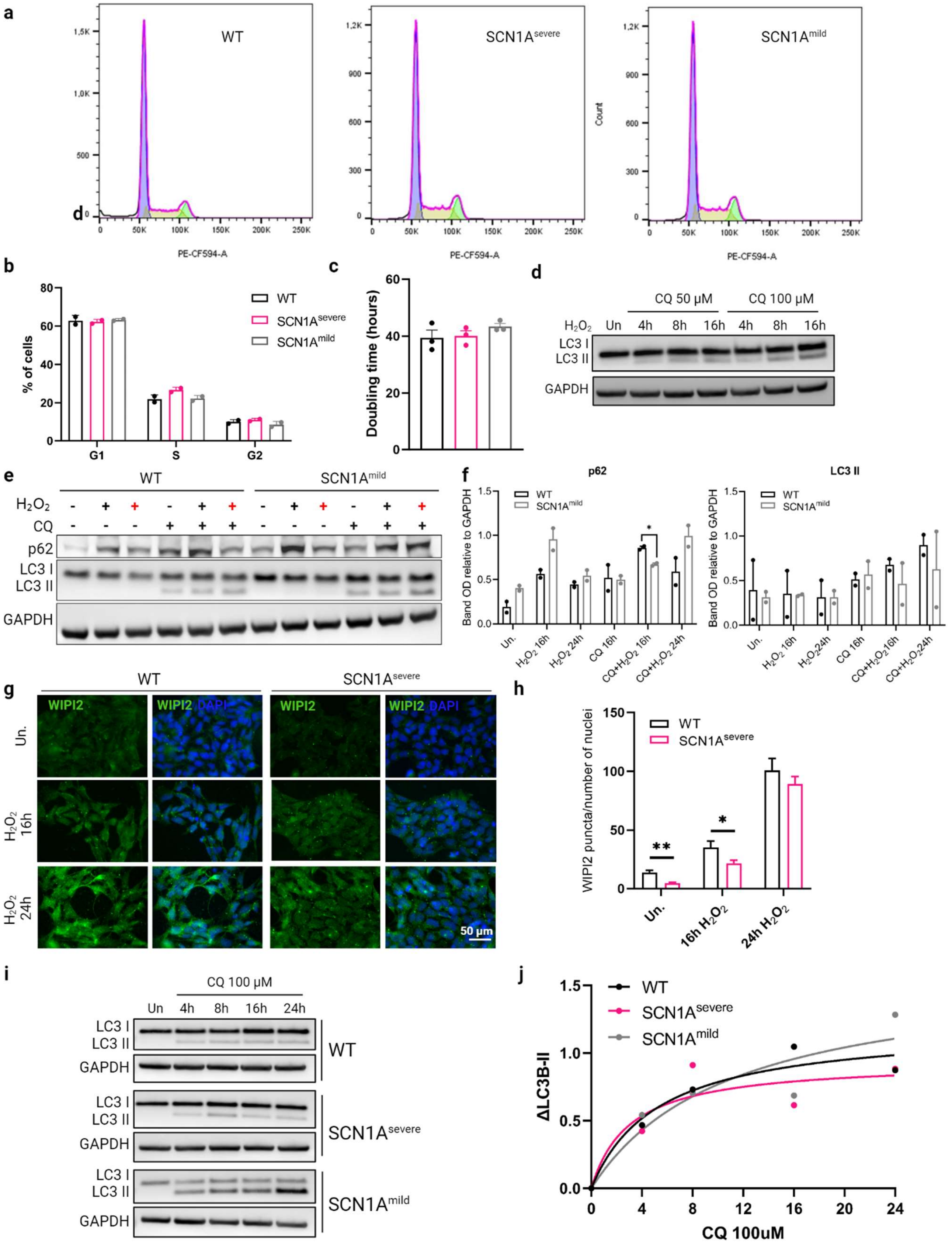

Supplementary Figures S4

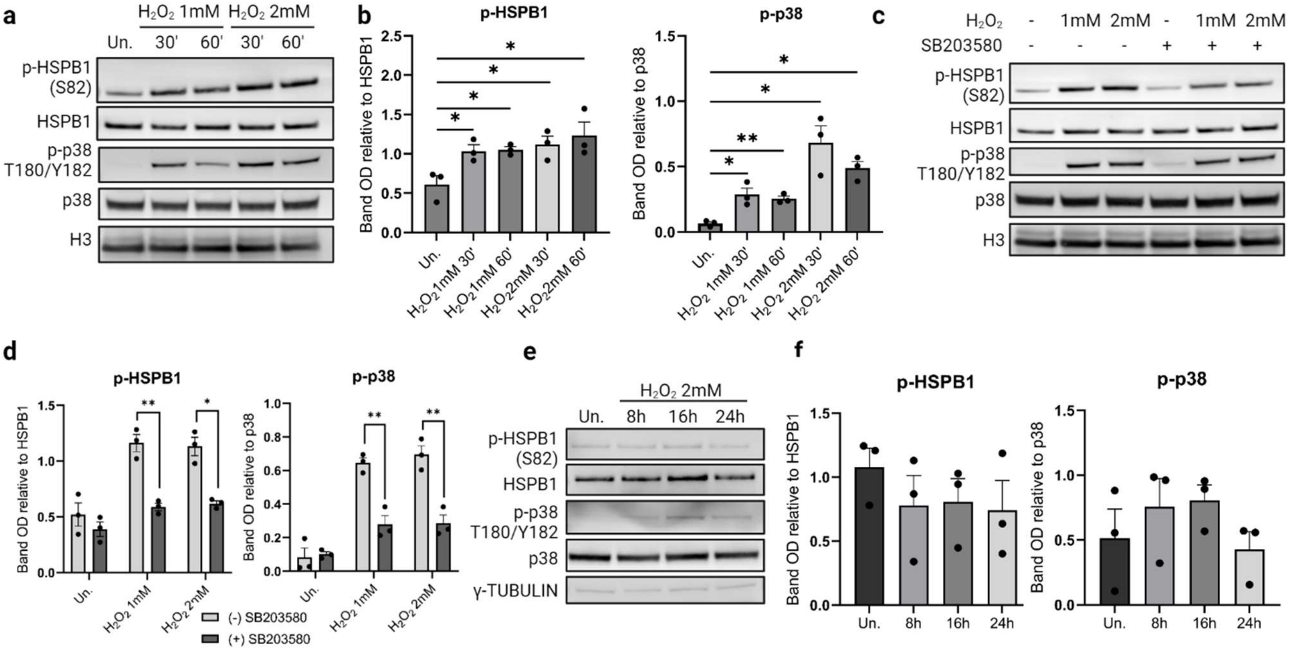

Supplementary Figures S5

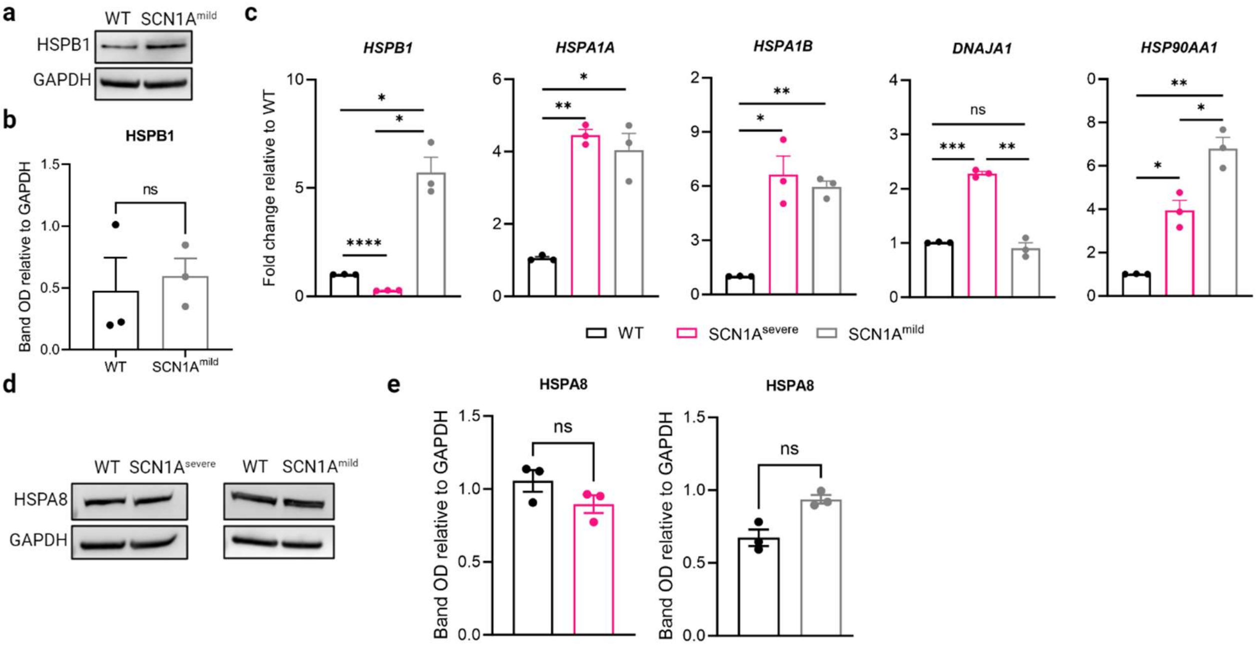

Supplementary Figures S6

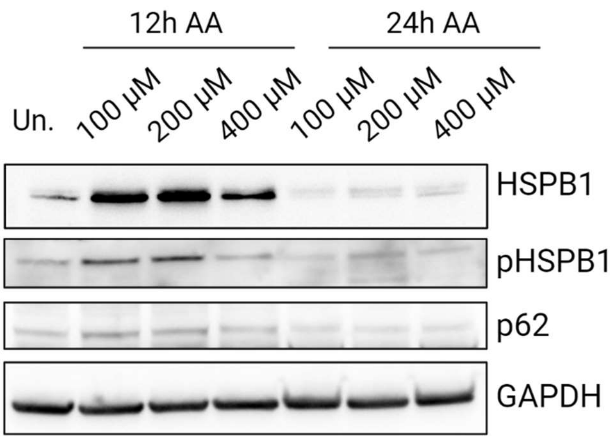

Supplementary Figure Legends

Supplementary Fig. S1 iNSCs characterization. Expression of the neural markers NESTIN and PAX6 in iNSCs.

Supplementary Fig. S2 Representative images of NRF2 in iNSCs under H_2O_2 treatment.

Supplementary Fig. S3 (**a**) Cell cycle analysis of WT, SCN1A^{severe} and SCN1A^{mild} iNSCs and (**b**) relative percentage of cells in G1, S and G2 phases. (**c**) Calculation of iNSCs doubling time using the equation (1). (**d**) Western blot analysis of LC3 I and LC3 II accumulation in iNSCs after treatment with the autophagy inhibitor cloroquine (CQ) 50µM or 100µM for 4h, 8h, 16h. (**e**) Western blot analysis of protein involved in the autophagy process in WT and SCN1A^{mild} cells treated with 2mM H_2O_2 only for 16 (black cross) and 24 hours (red cross) or in combination with the autophagy inhibitor chloroquine (CQ). (**f**) Quantification of immunoblot bands of panel (**e**). (**g**) Immunofluorescence analysis of WIPI2 under basal condition and after 16h treatment with 2mM H_2O_2. (**h**) Quantification of WIPI2 signal showed a significant reduction of WIPI2 puncta in patient iNSCs both in the untreated and treated condition. *$p<0.05$, **$p<0.01$, ****$p<0.0001$, t-test. (**i**) Analysis of autophagic flux by LC3 II signal detection in WT and patients iNSCs treated with 100 µm CQ for 4, 8, 16 and 24 hours. (**j**) Quantification of autophagic flux as change in LC3 II calculated with the equation (2). LC3 II normalized to GAPDH is displayed for the three cell lines with the best-fit non-linear regression.

Supplementary Fig. S4 Activation of the p38/HSPB1 axis during oxidative stress. (**a**) Phosphorylation of HSPB1 and p38 was observed 30 minutes after treatment with H_2O_2 (1mM and 2mM), while decreases after 60 minutes. The phosphorylation levels of the two proteins positively correlates with the concentration of the H_2O_2 stimulus. GAPDH was used as loading control. (**b**)

Quantification of western blot bands displayed in (**a**). (**c**) Validation of p38/HSPB1 axis. To effectively ascertain the correlation between p38 and HSPB1 activation during oxidative stress, iNSCs were treated with 1mM and 2mM H_2O_2 alone, as well as in combination with the p38 inhibitor SB203580 at a concentration of 10µM, for a duration of 30 minutes. Phosphorylation levels of both p38 and HSPB1 decreased after co-treatment with H_2O_2 (at both 1mM and 2mM concentrations) and SB203580. Histone H3 was used as loading control. (**d**) Quantification of western blot bands displayed in (**c**). (**e**) Phosphorylation of HSPB1 and p38 after 8, 16 and 24 hours of treatment with H_2O_2 2mM. γ-tubulin was used as loading control. (**f**) Quantification of western blot bands displayed in (**e**). For all western blot quantification, data are presented as mean ± SEM of three biological replicates. *p<0.05, **p<0.01, t-test.

Supplementary Fig. S5 (**a**) Expression of HSPB1 protein in WT and $SCN1A^{mild}$ iNSCs and (**b**) relative quantification. (**c**) Analysis of HSPB1 and other Heat Shock Protein family members genes (HSPA1A and HSPA1B (both HSP70 members), DNAJA1 (HSP40), HSP90AA1 (HSP90)) in healthy and mutated iNSCs. GAPDH was used as housekeeping gene. Data are presented as mean ± SEM of three biological replicates. *p<0.05, **p<0.01, ***p<0.001, ****p<0.0001, t-test. (**d**) Protein expression of HSPA8 (HSP70) in healthy and mutated iNSCs. GAPDH was used as loading control. (**e**) Quantification of western blot bands showed in (**d**) demonstrated the absence of significant differences in the expression of HSP8 protein among the three cell lines used in this study.

Supplementary Fig. S6 Effect of AA on HSPB1 and p62 expression in iNSCs of the patient. HSPB1 and p62 accumulates when cells were subjected to treatment with 100 and 200 µM AA for 12-hours duration; higher concentration (400 µM) or extended treatment period (24 hours) did not yield differences in the expression levels of these two proteins in respect to untreated condition.

Table S1. List of primers.

Target gene	Primer sequence
CAT	FW: CTCAGGTGCGGGCATTCTAT – RV: AGATCCGGACTGCACAAAGG
SOD1	FW: GGAGACTTGGGCAATGTGAC – RV: CACAAGCCAAACGACTTCCA
SOD3	FW: CTGGGTGCAGCTCTCTTTTC – RV: TTGGCGTACATGTCTCGGAT
GPX1	FW: TATCGAGAATGTGGCGTCCC – RV: CCGGACGTACTTGAGGGAAT
GPX3	FW: GGCTTTCCCTGCAACCAATT – RV: AAGAGGCGGTCAGATGTACC
NRF2	FW: CATCCAGTCAGAAACCAGTGG – RV: AATGAAGACTGGGCTCTCGA
NQO1	FW: ATCCTGCCGAGTCTGTTCTG – RV: TTTCAGAATGGCAGGGACTC
HMOX1	FW: TGCTGACCCATGACACCAAG – RV: AAGCCCTACAGCAACTGTCG
Bax	FW: GGACGAACTGGACAGTAACATGG – RV: GCAAAGTAGAAAAGGGCGACAAC
Bcl2	FW: ATCCCCCTGTGGATGACTCAG – RV: CAGCCAGGAGAAATCAAACAGAGG
HSPB1	FW: TGGATGTCAACCACTTCGCC – RV: AGGGAGGAGGAAACTTGGGT
DNAJA1	FW: GTAAAAGCTGCAACGGAAGG – RV: CTCCAGTCCTGGTTCTTGGT
HSPA1A	FW: CCGAGAAGGACGAGTTTGAG – RV: AATCTTGGAAAGGCCCCTAA
HSPA1B	FW: CCCTACCATTGAGGAGGTG – RV: AAACTCGTACAGAAGGTGGC
HSP90AA1	FW: AGGTGAACCTATGGGTCGTG – RV: TTCAGCCTCATCATCGCTTA
GAPDH	FW: TCCTCTGACTTCAACAGCGA – RV: GGGTCTTACTCCTTGGAGGC

Table S2. List of primary antibodies.

Antibody	*Host species*	*Dilution WB*	*Dilution IF*	*Cat.No*	*Company*
**Nestin**	Mouse	-	1:1000	60091	STEMCELL Tech
**PAX6**	Rabbit	-	1:100	42-6600	Invitrogen
**Nrf2**	Rabbit	1:1000	1:400	#12721	Cell Signaling Technology
**Bcl-xl**	Rabbit	1:1000	-	#2762	Cell Signaling Technology
**Caspase-3 (3G2)**	Mouse	1:1000	-	9668	Cell Signaling Technology
**p62/SQSTM1**	Mouse	1:5000	1:50	ab56416	Abcam
**LC3B**	Rabbit	1:1000	-	#2775	Cell Signaling Technology
**Beclin1**	Rabbit	1:1000	-	#3738	Cell Signaling Technology
**Phospho-AKT (T308)**	Rabbit	1:1000	-	#13038S	Cell Signaling Technology
**AKT**	Rabbit	1:1000	-	#9272S	Cell Signaling Technology
**WIPI2**	Rabbit	-	1:100	ab109459	Abcam
**Phospho-p38 MAPK (Thr180/Tyr182)**	Mouse	1:1000	-	#9216	Cell Signaling Technology
**p38 MAPK**	Rabbit	1:1000	-	#9212	Cell Signaling Technology
**Phospho-HSP27 (Ser82)**	Rabbit	1:1000	1:25	#2401	Cell Signaling Technology
**HSP27**	Mouse	1:1000	-	#2402	Cell Signaling Technology
**HSPA8**	Rabbit	1:1000	-	#D12F2	Cell Signaling Technology
**HMGB1**	Rabbit	-	1:50	#3935	Cell Signaling Technology
**Anti-Y-Tubulin HRP**	-	1:1000	-	sc-17787	Santa Cruz Technology
**Histone H3**	Rabbit	1:1000	-	ab1791	Abcam
**GAPDH**	Rabbit	1:1000	-	bs10900R	Bioss Antibodies

Uncropped western blot images

Bands showed in the main text and supplementary materials and used for the Band OD quantification.

Images showed in FIG. 2b

First line: WT untreated, second lane: WT 8h tr., third lane: WT 16h tr., fourth lane: WT 24h tr., fifth lane: SCN1A^{severe} untreated, sixth lane: SCN1A^{severe} 8h tr., seventh lane: SCN1A^{severe} 16h tr., eight lane: SCN1A^{severe} 24h tr.

Nrf2 – 97 kDa (1° replicate)

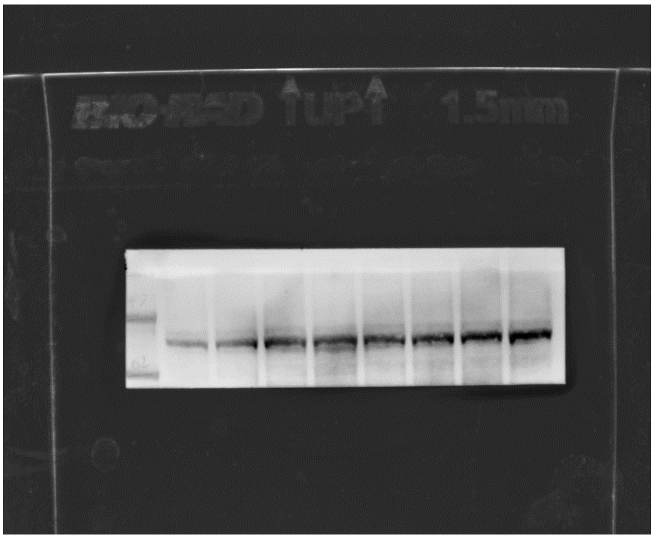

GAPDH – 36 kDa (1° replicate)

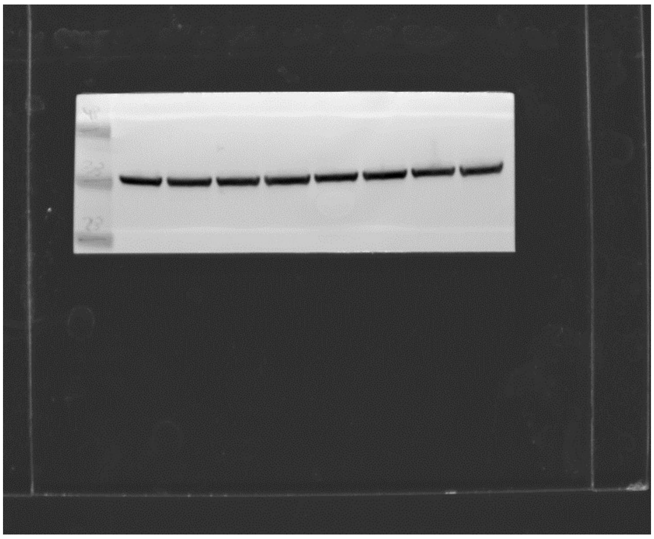

Nrf2 (2° replicate)

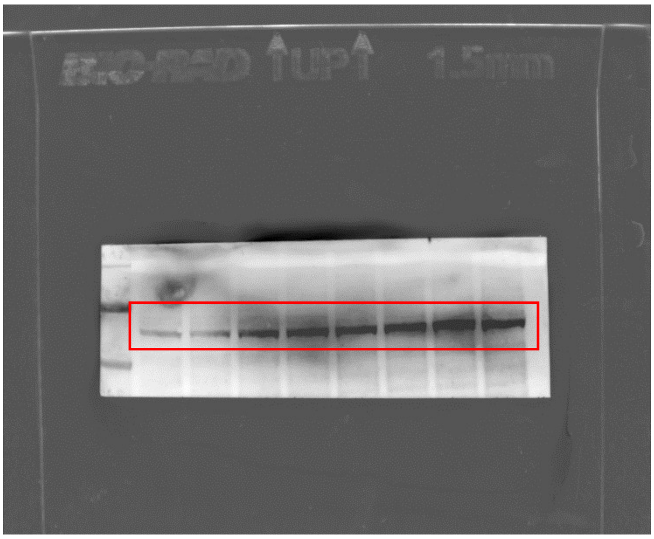

GAPDH (2° replicate)

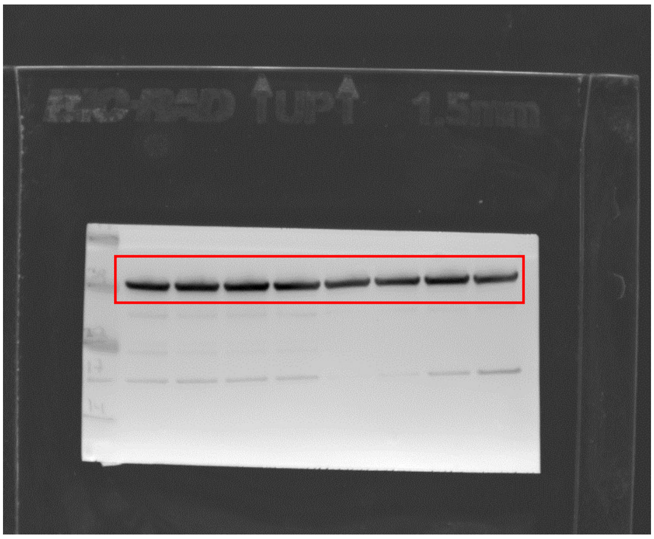

Nrf2 (3° replicate)

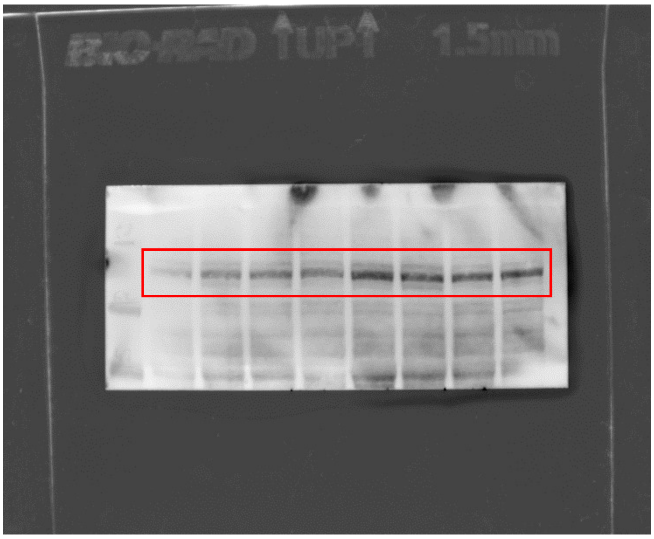

GAPDH (3° replicate)

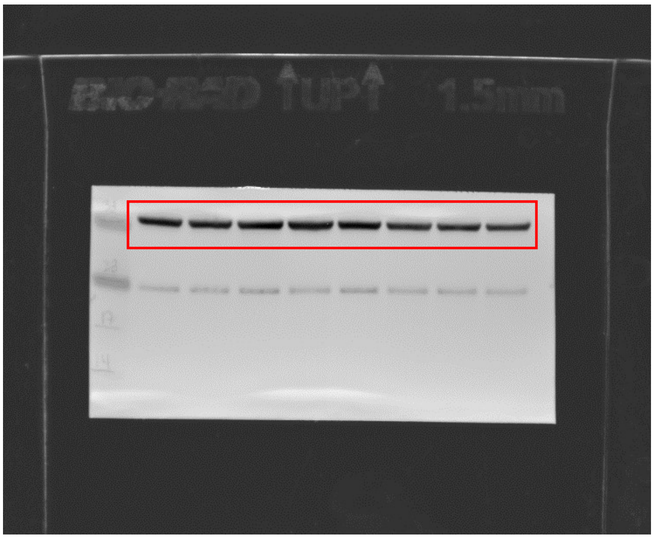

Images showed in FIG. 3e

First line: WT untreated, second lane: WT 8h tr., third lane: WT 16h tr., fourth lane: WT 24h tr., fifth lane: SCN1A^{severe} untreated, sixth lane: SCN1A^{severe} 8h tr., seventh lane: SCN1A^{severe} 16h tr., eighth lane: SCN1A^{severe} 24h tr.

Bcl-xl – 30 kDa (1° replicate)

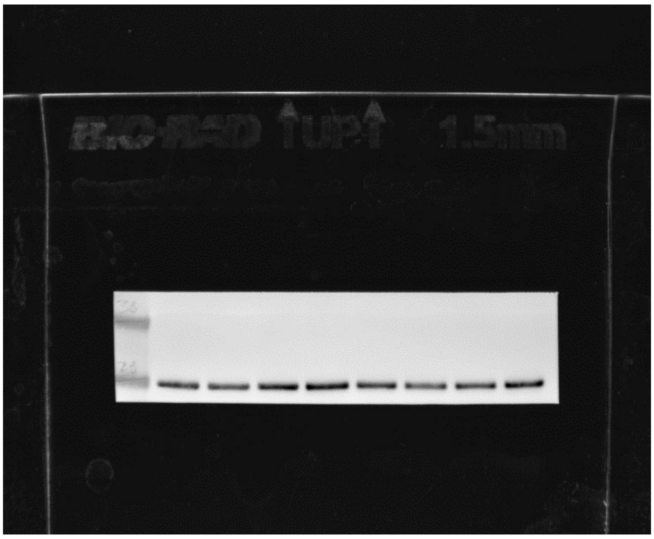

GAPDH – 36 kDa (1° replicate)

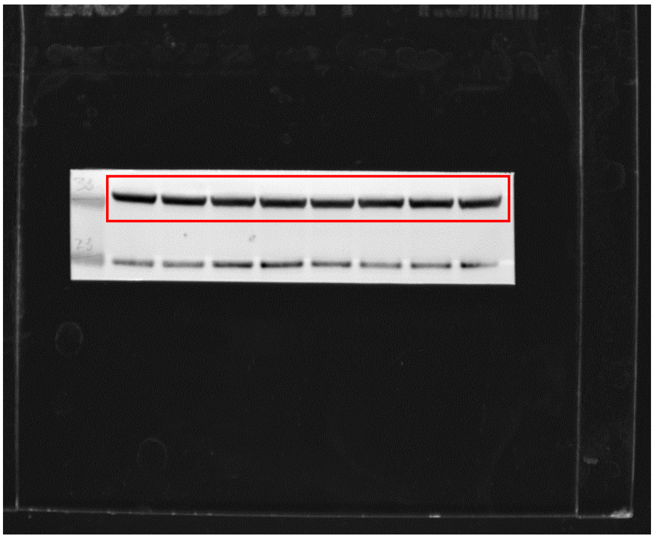

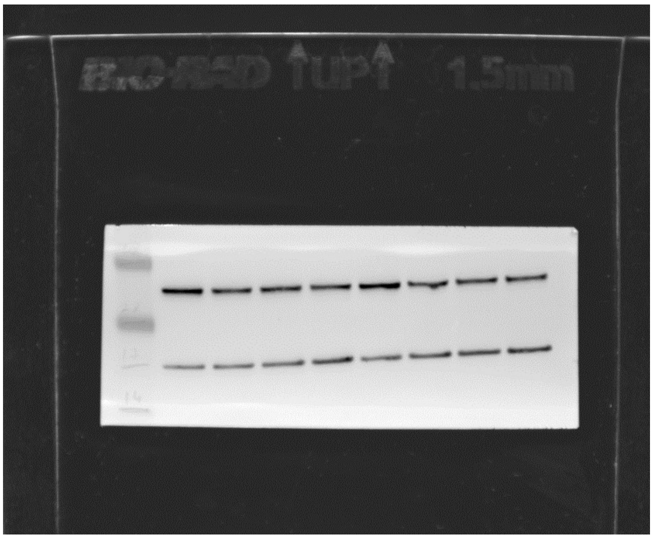

Casp-3 – 35 kDa full length, 17 kDa cleaved fragment (1° replicate)

Bcl-xl (2° replicate)

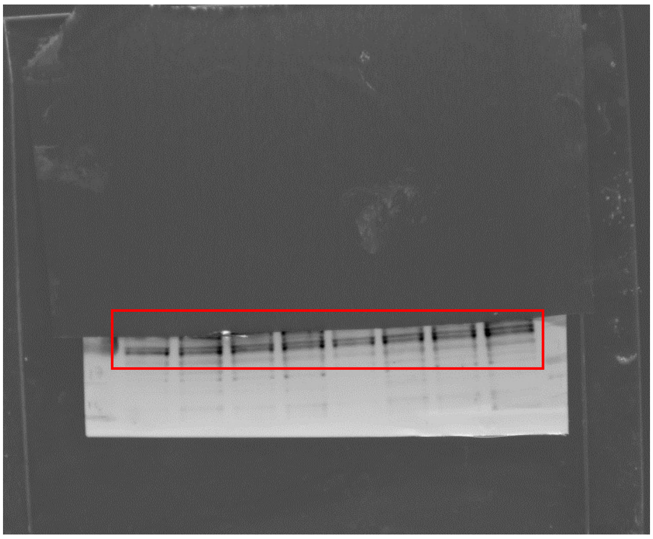

GAPDH (2° replicate)

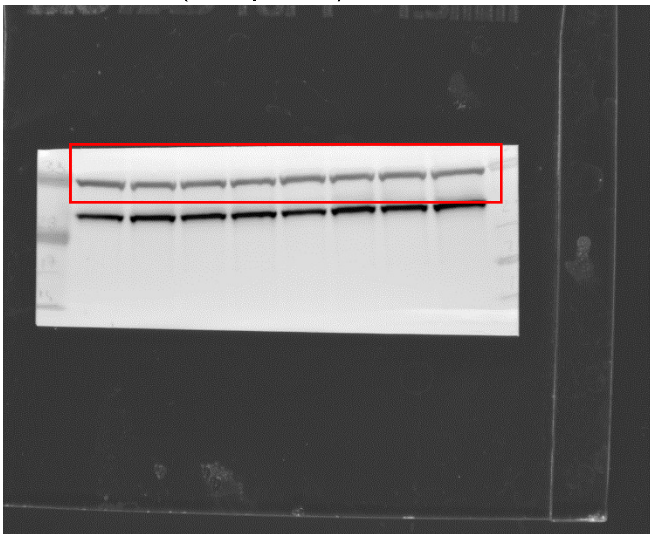

Casp-3 (2° replicate)

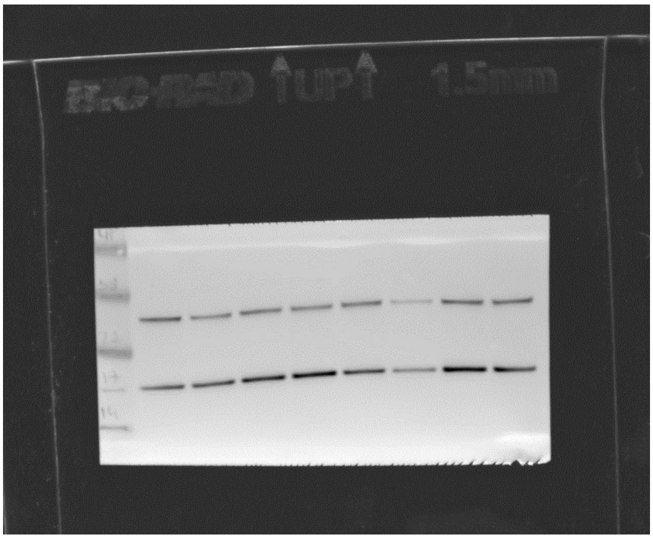

GAPDH (2° replicate)

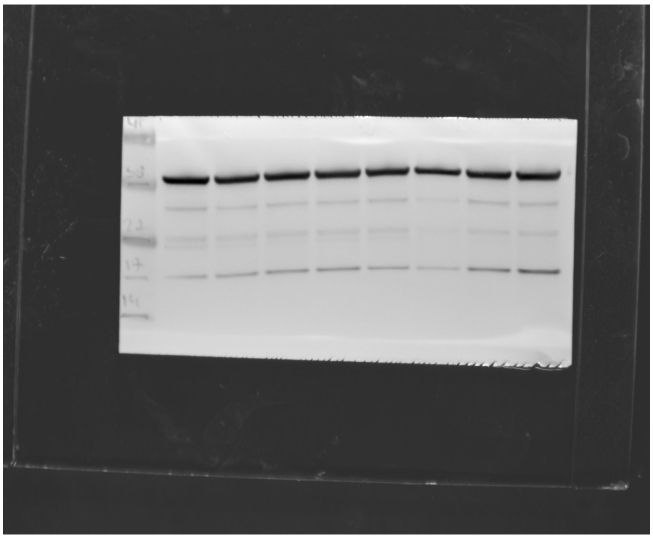

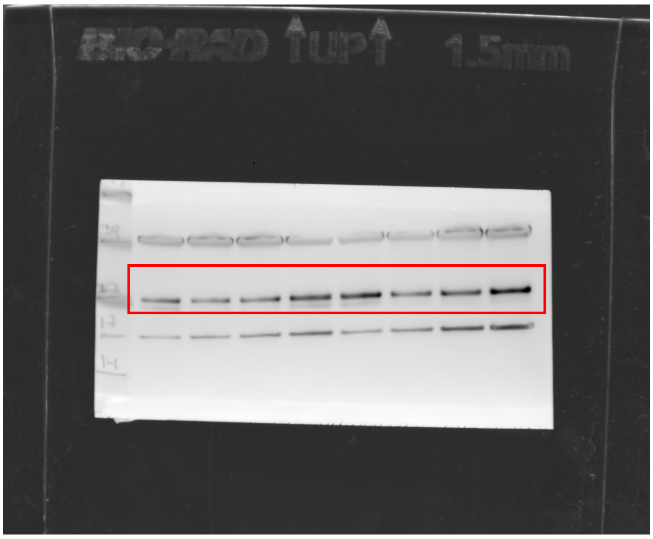

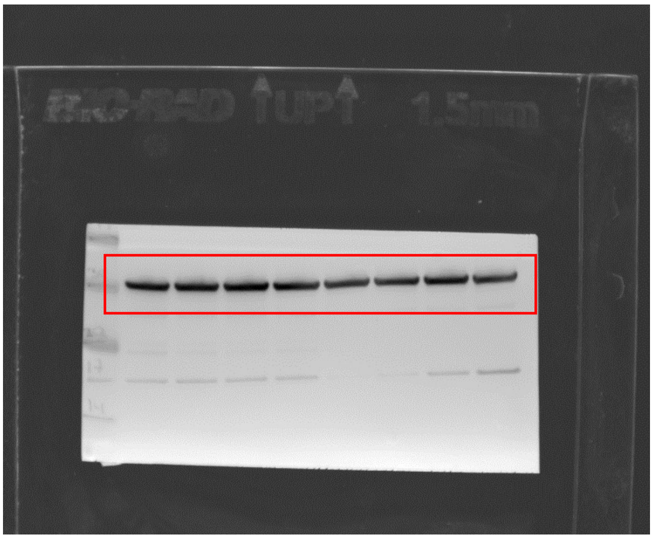

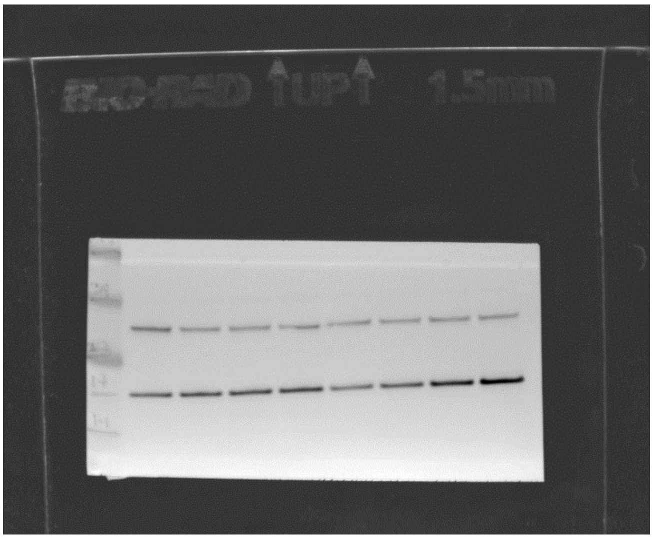

Casp-3 (3° replicate)

Images showed in FIG. 4b

First line: WT untreated, second lane: WT H_2O_2 16h tr., third lane: WT H_2O_2 24h tr., fourth lane: WT CQ 16h tr., fifth lane: WT CQ 16h+ H_2O_2 16h tr., sixth lane: WT CQ 16h+ H_2O_2 24h tr., seventh lane: SCN1A^{severe} untreated, eighth lane: SCN1A^{severe} H_2O_2 16h tr., nineth lane: SCN1A^{severe} H_2O_2 24h tr., tenth lane: SCN1A^{severe} CQ 16h tr., eleventh line: SCN1A^{severe} CQ 16h+ H_2O_2 16h tr., twelfth line: SCN1A^{severe} CQ 16h+ H_2O_2 24h tr.

p62 – 62 kDa (1° replicate)

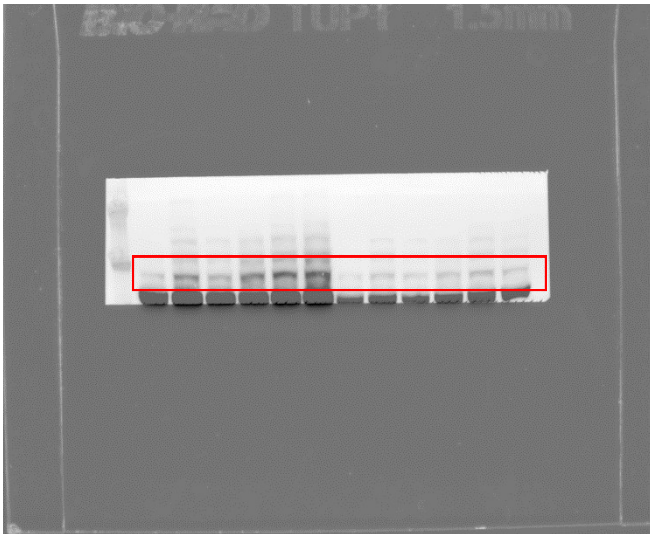

LC3I-II – 14 kDa (1° replicate)

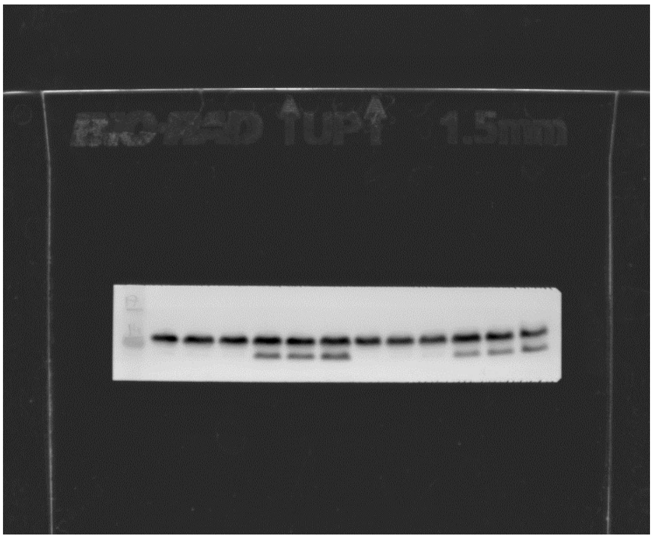

GAPDH – 36 kDa (1° replicate)

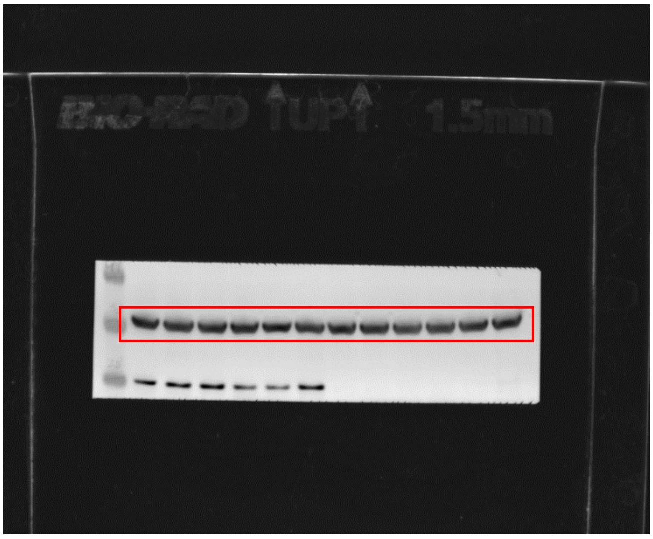

p62 (2° replicate)

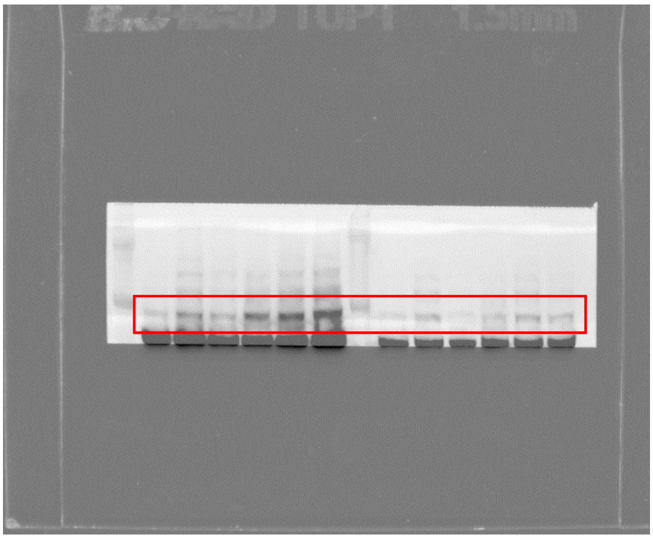

LC3I-II (2° replicate)

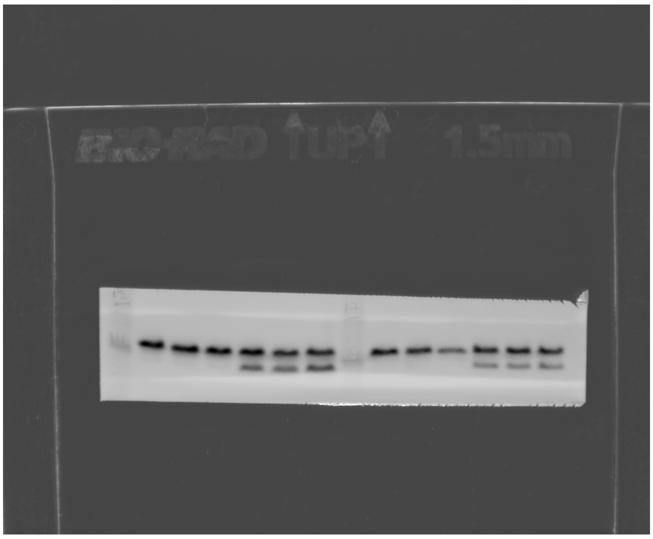

GAPDH (2° replicate)

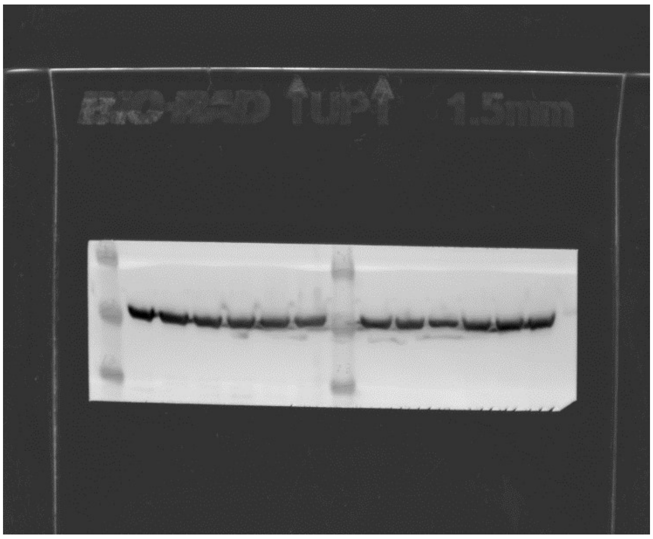

Images showed in FIG. 4h

First line: WT untreated, second lane: WT 8h tr., third lane: WT 16h tr., fourth lane: WT 24h tr., fifth lane: SCN1A^{severe} untreated, sixth lane: SCN1A^{severe} 8h tr., seventh lane: SCN1A^{severe} 16h tr., eighth lane: SCN1A^{severe} 24h tr.

Beclin1 – 52 kDa (1° replicate)

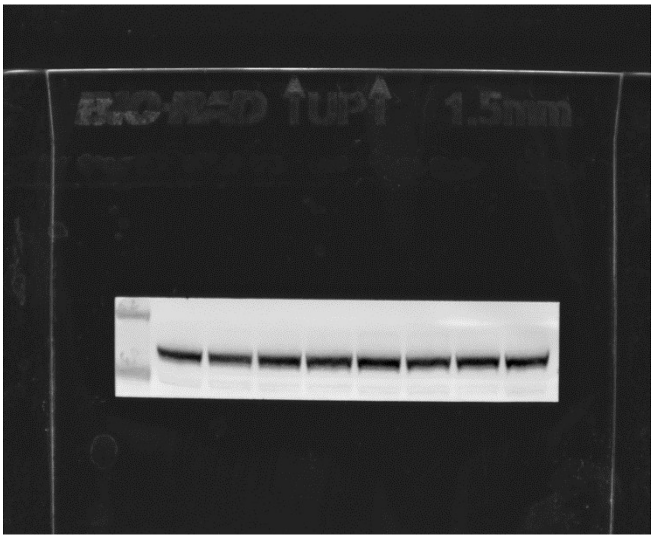

pAKT(T308) – 62 kDa (1° replicate)

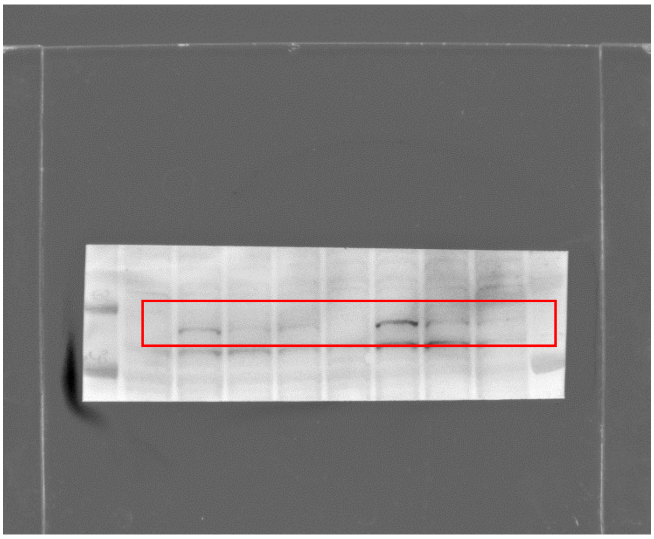

AKT – 62 kDa (1° replicate)

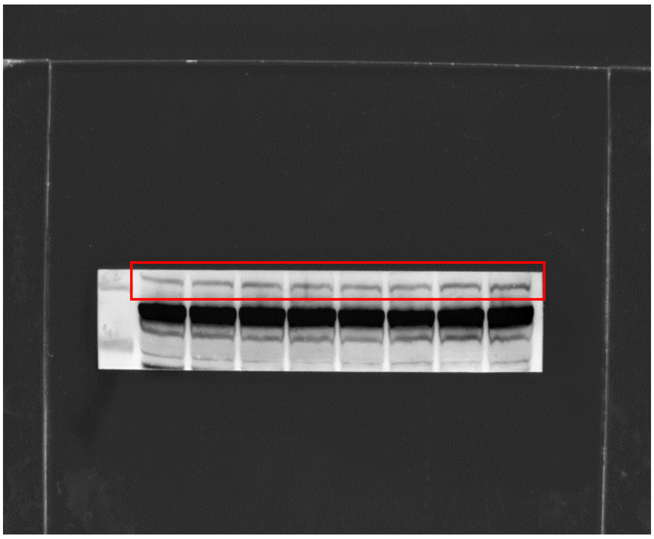

GAPDH – 36 kDa (1° replicate)

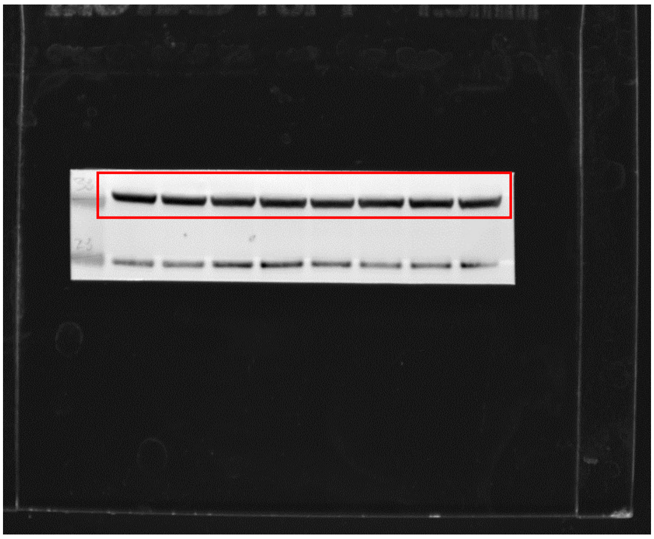

Beclin1 (2° replicate)

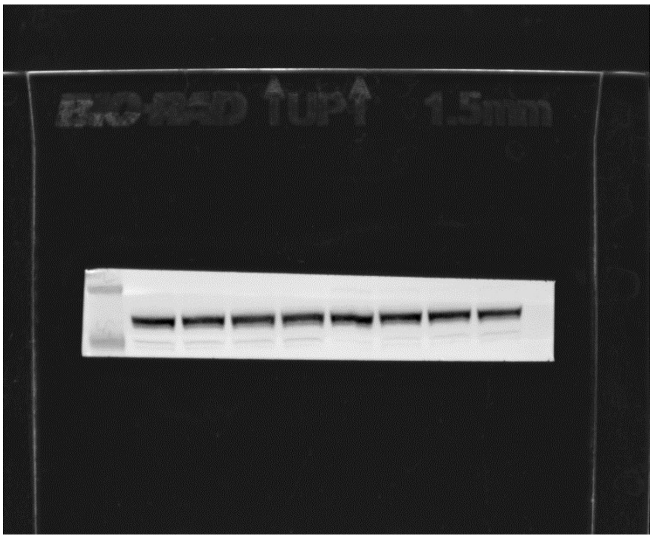

GAPDH (2° replicate)

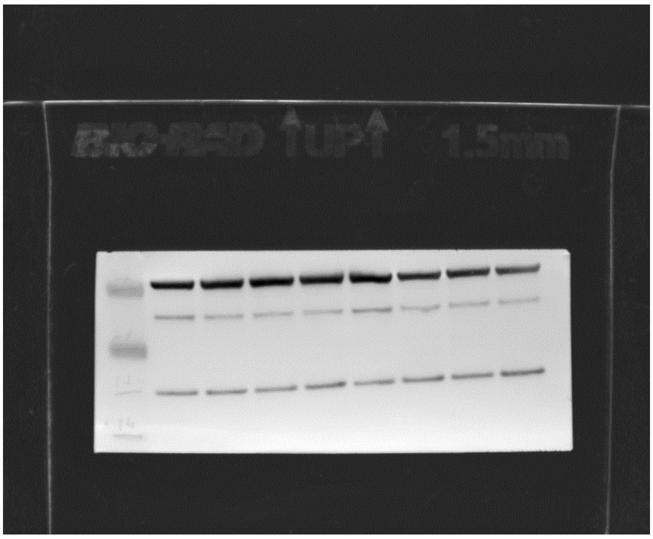

pAKT(T308) (2° replicate)

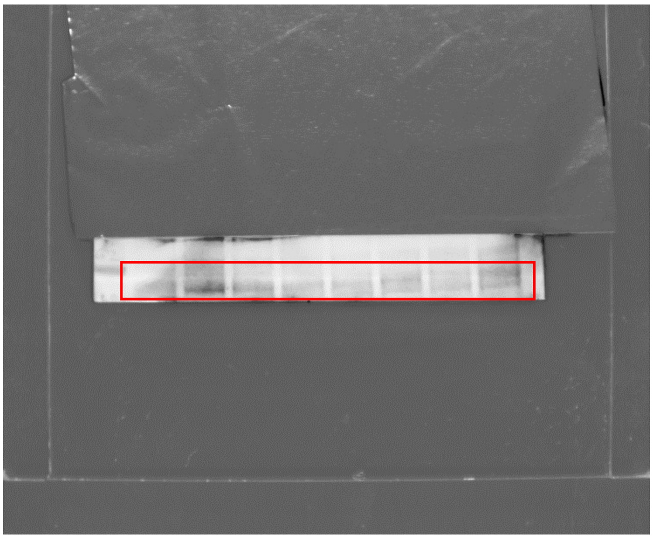

AKT (2° replicate)

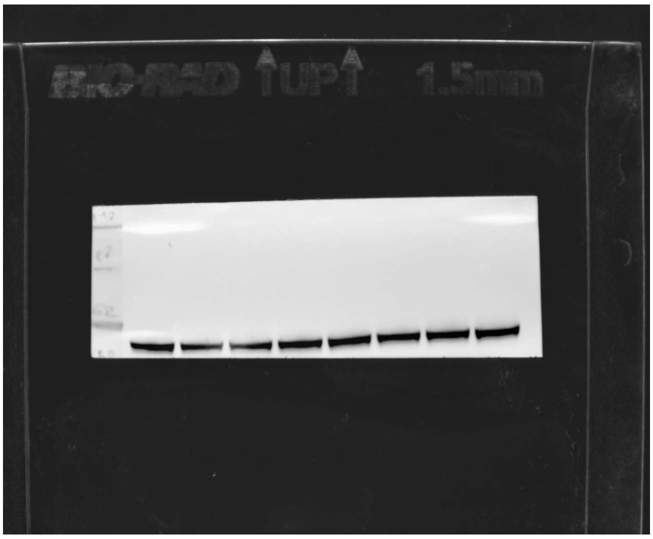

GAPDH (2° replicate)

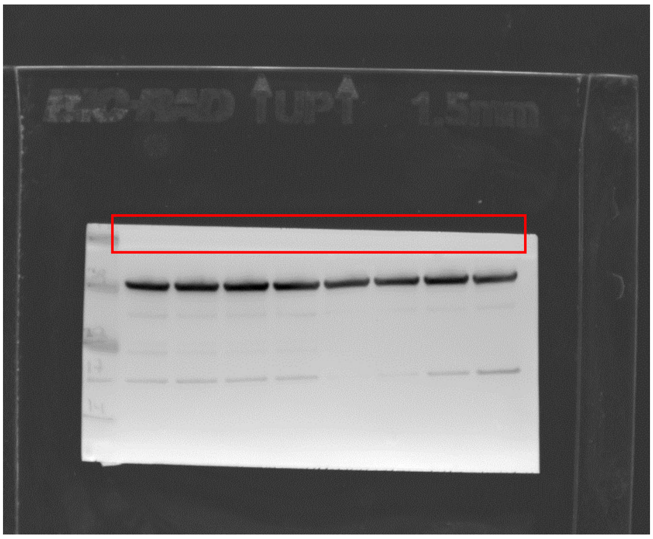

Beclin1 (3° replicate)

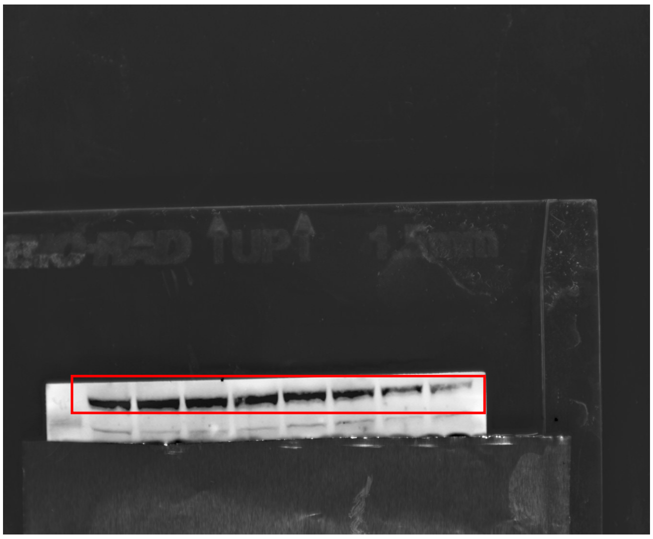

GAPDH (3° replicate)

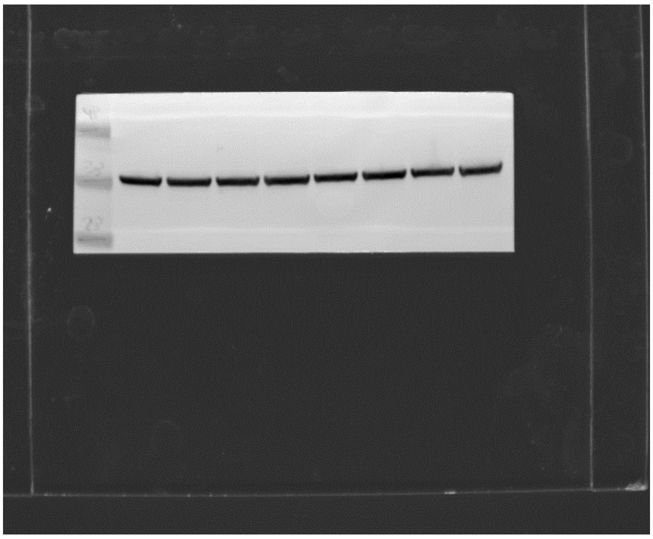

pAKT(T308) (3° replicate)

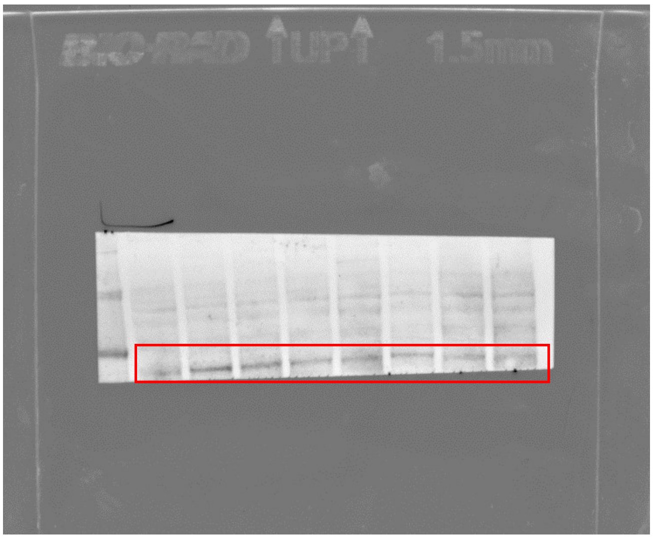

AKT (3° replicate)

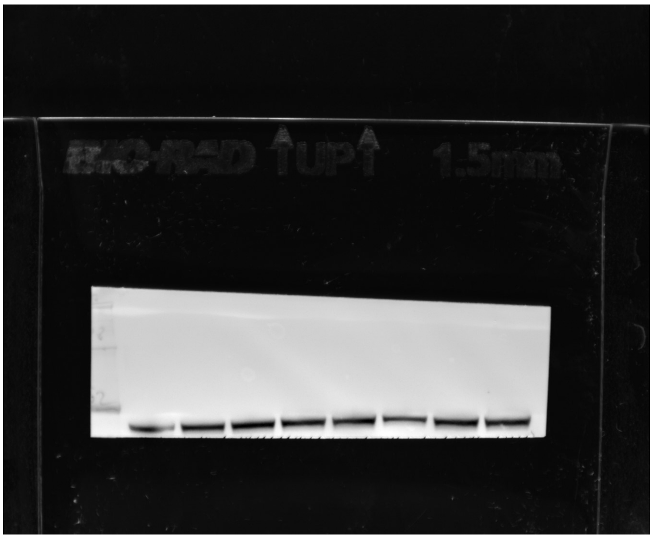

GAPDH (3° replicate)

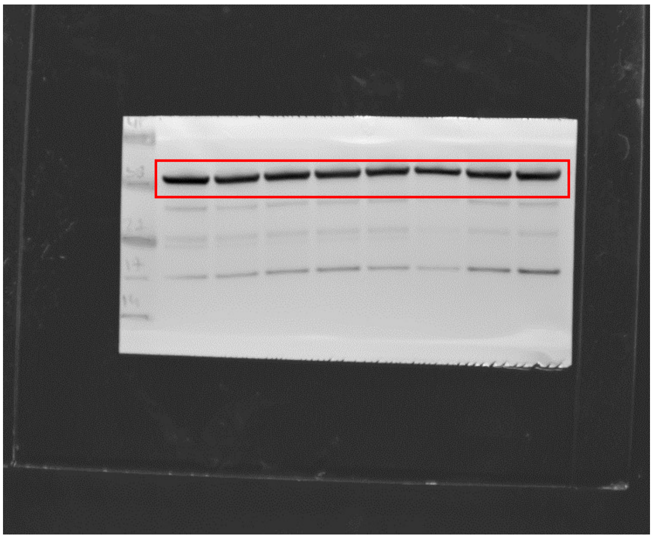

Images showed in FIG. 5a

First line: WT untreated, second lane: WT H_2O_2 1mM tr., third lane: WT H_2O_2 2mM tr., fourth lane: $SCN1A^{severe}$ untreated, fifth lane: $SCN1A^{severe}$ H_2O_2 1mM tr., sixth lane: $SCN1A^{severe}$ H_2O_2 2mM tr.

p-p38 – 38 kDa (1° replicate)

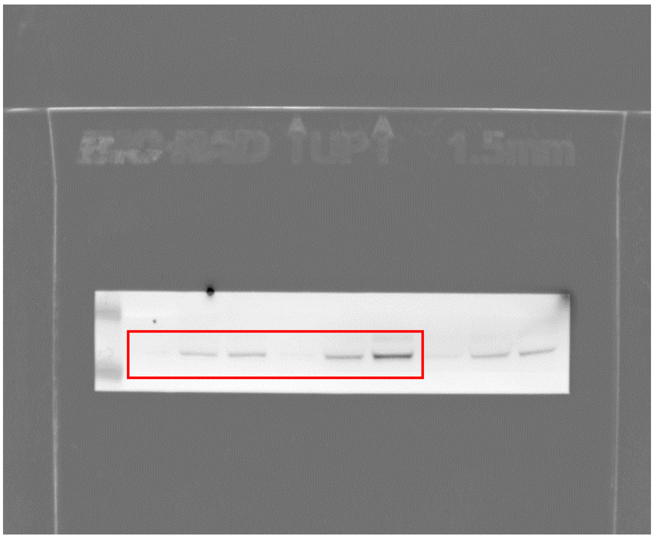

p38 – 38 kDa (1° replicate)

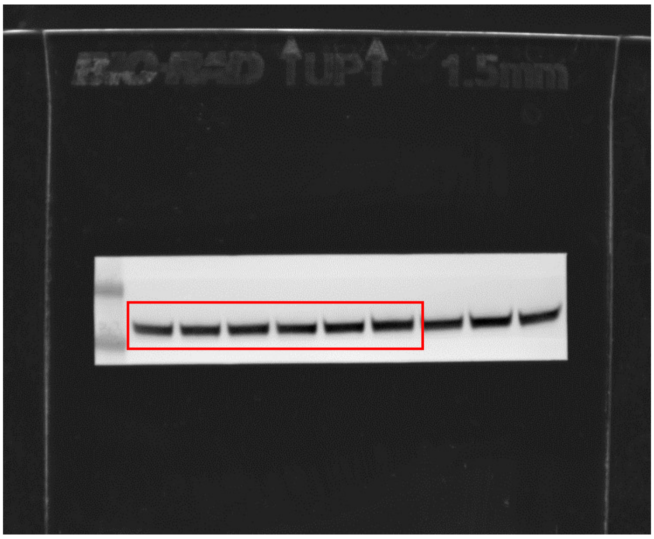

pHSPB1 – 27 kDa (1° replicate)

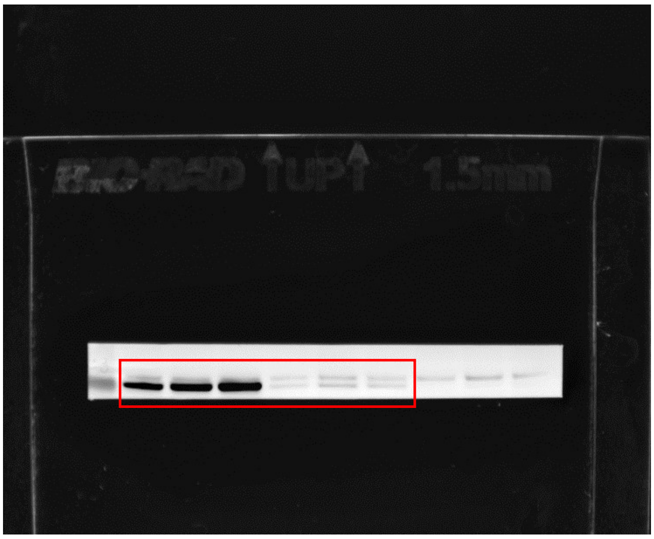

HSPB1 – 27 kDa (1° replicate)

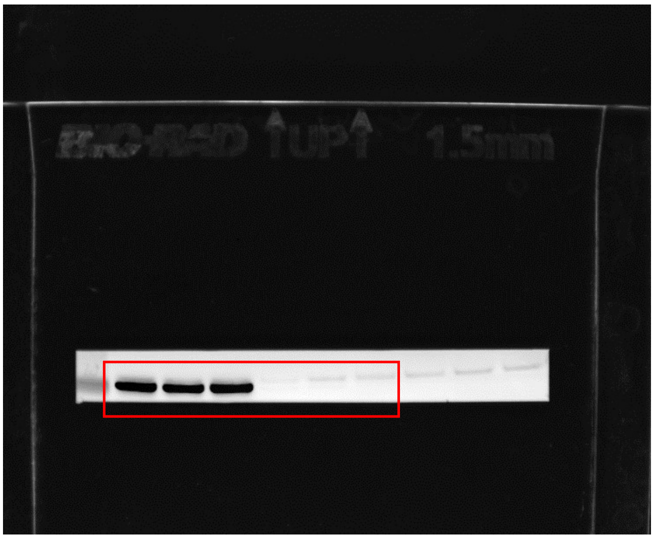

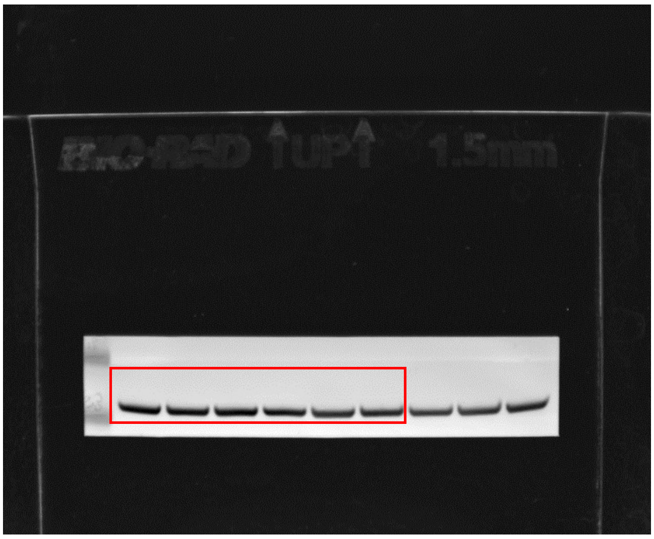

GAPDH – 36 kDa (1° replicate)

p-p38 – 38 kDa (2° replicate)

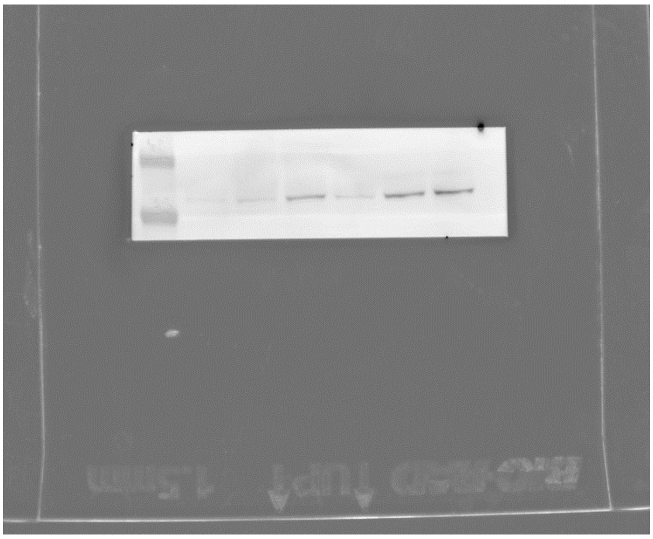

p38 – 38 kDa (2° replicate)

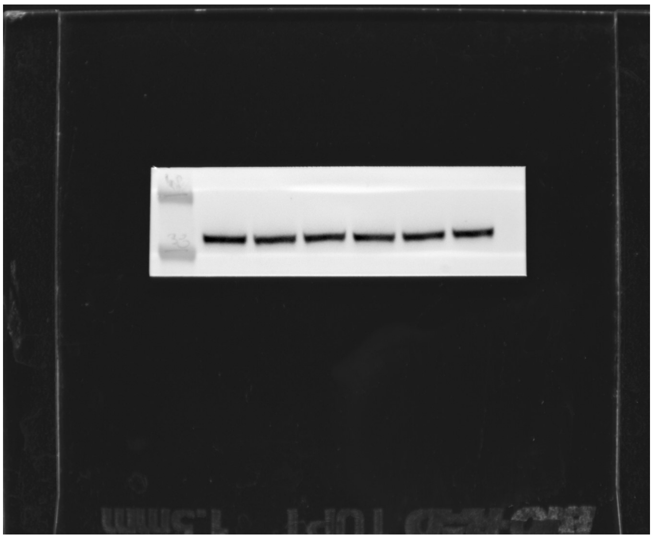

pHSPB1 – 27 kDa (2° replicate)

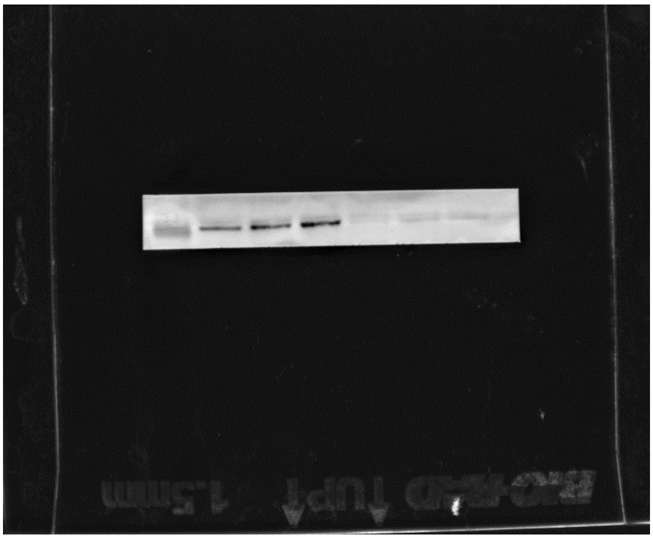

HSPB1 – 27 kDa (2° replicate)

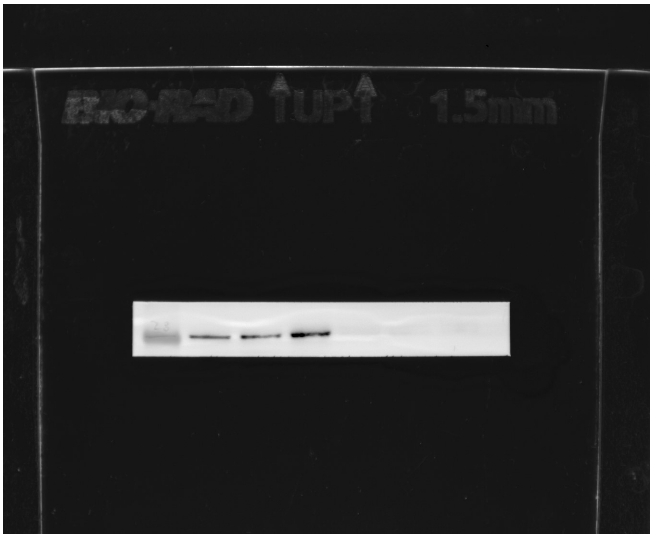

H3 – 17 kDa (2° replicate)

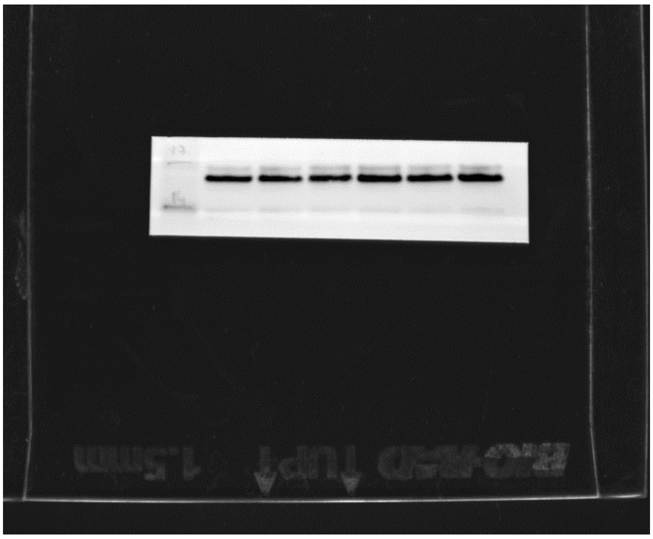

p-p38 – 38 kDa (3° replicate)

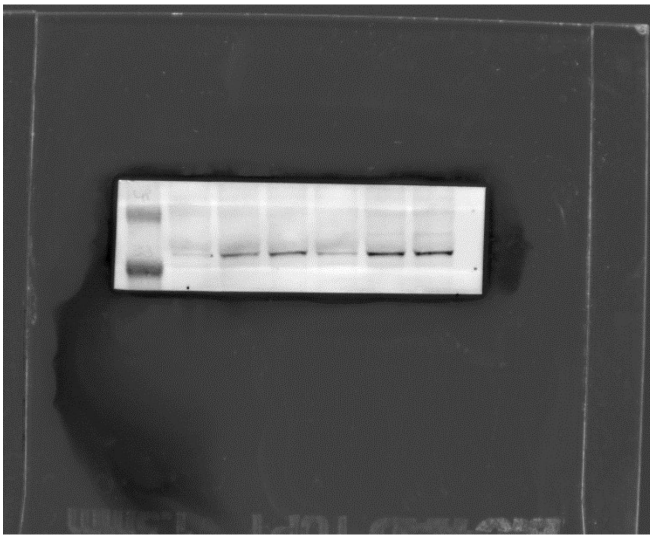

p38 – 38 kDa (3° replicate)

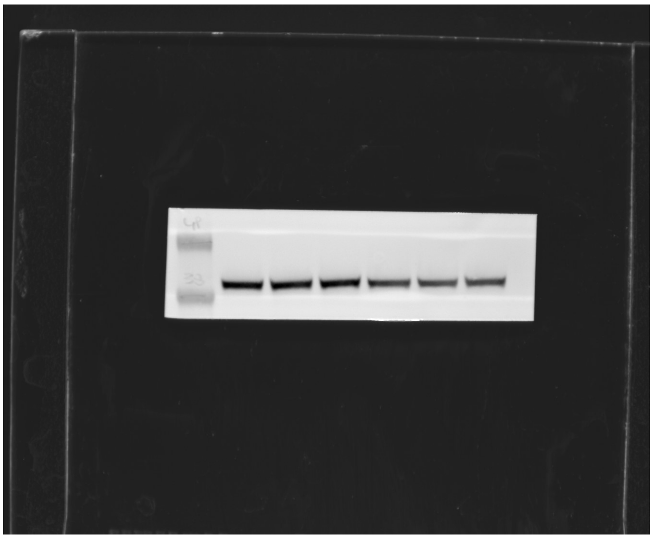

pHSPB1 (3° replicate)

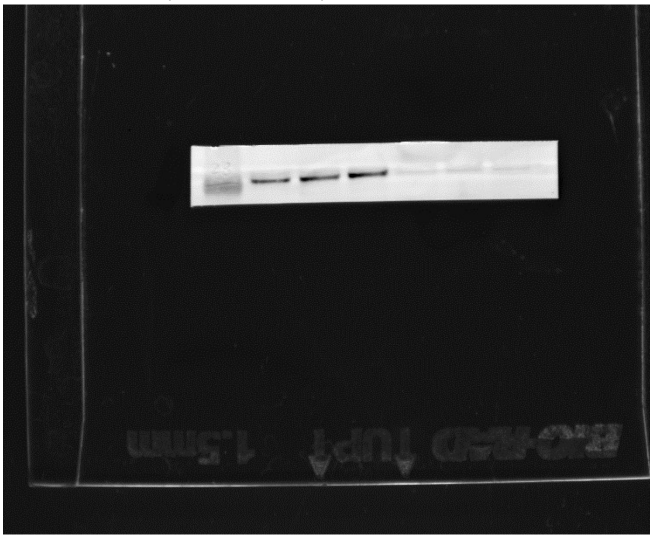

HSPB1 (3° replicate)

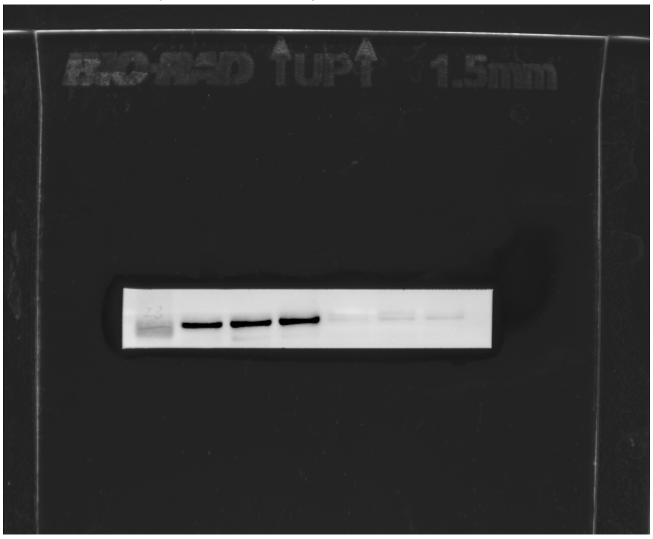

H3 (3° replicate)

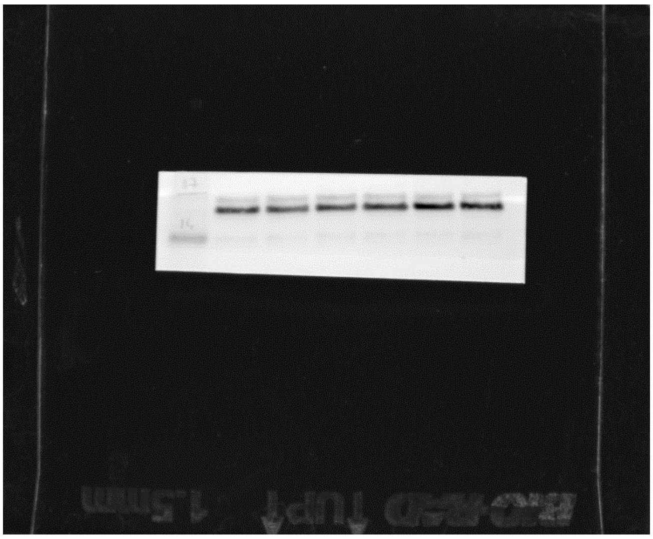

Images showed in FIG. 6a

First line: WT untreated, second line: SCN1A^{severe} untreated, third lane: SCN1A^{severe} 12h AA 100uM tr

p62 – 62 kDa (1° replicate)

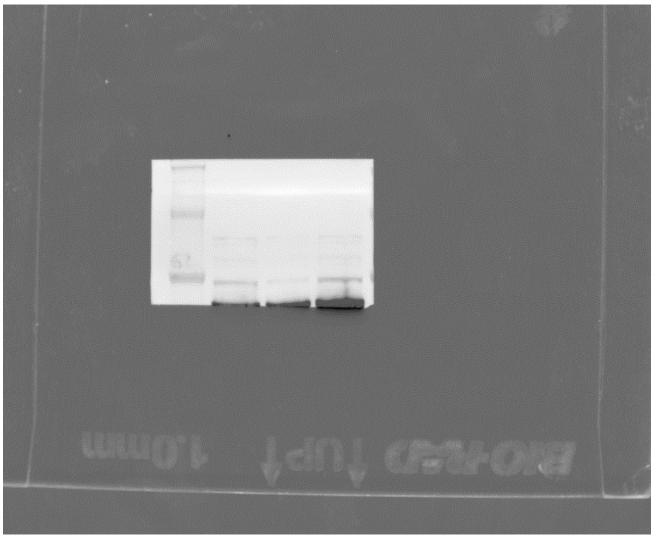

HSPB1 – 27 kDa (1° replicate)

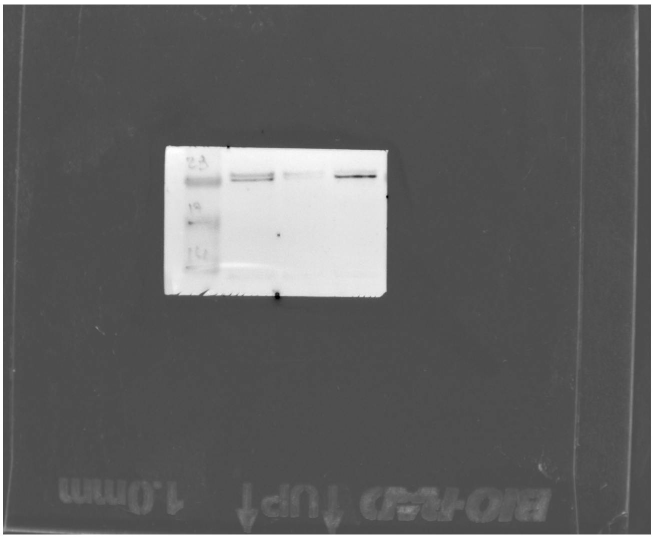

GAPDH – 36 kDa (1° replicate)

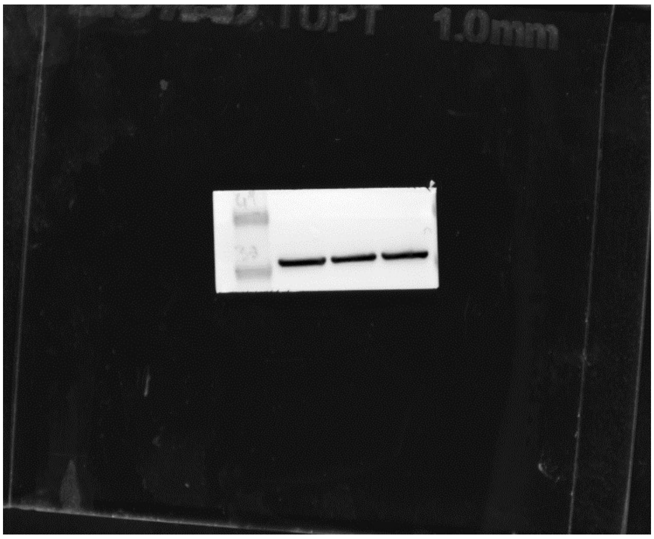

p62 (2° replicate)

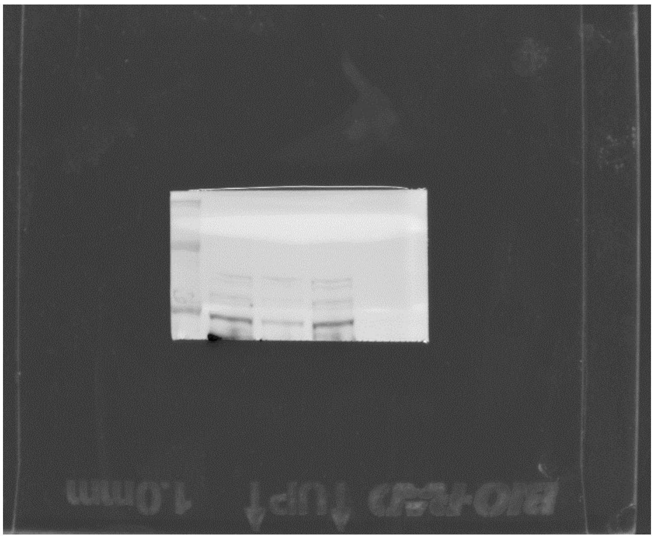

HSPB1 (2° replicate)

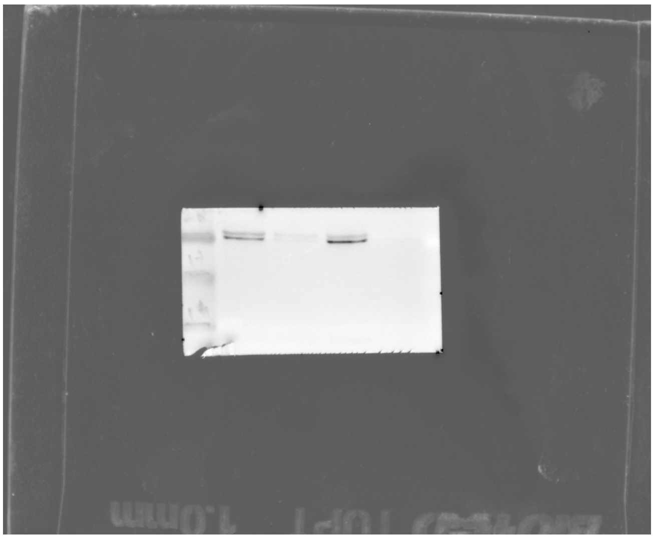

GAPDH (2° replicate)

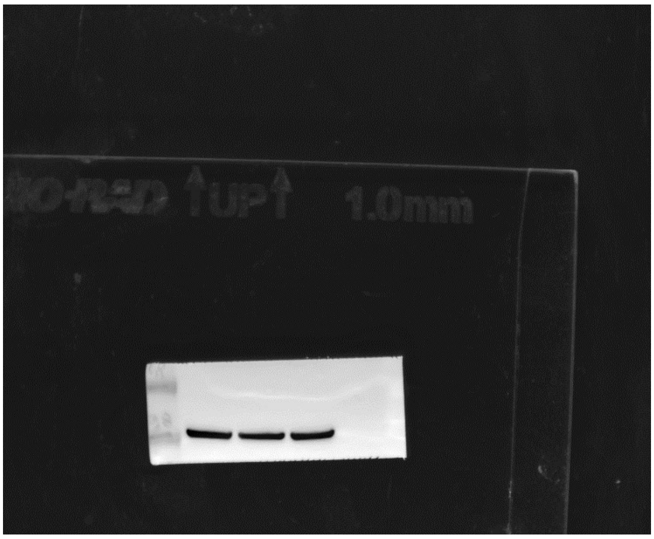

Images showed in Supplementary Fig. S3d

First line: WT untreated, second lane: WT 4h CQ 50uM tr., third lane: WT 8h CQ 50uM tr., fourth lane: WT 16h CQ 50uM tr., fifth lane: WT 4h CQ 100uM tr., sixth lane: WT 8h CQ 100uM tr., seventh lane: WT 16h CQ 100uM tr.

LC3I-II – 14 kDa

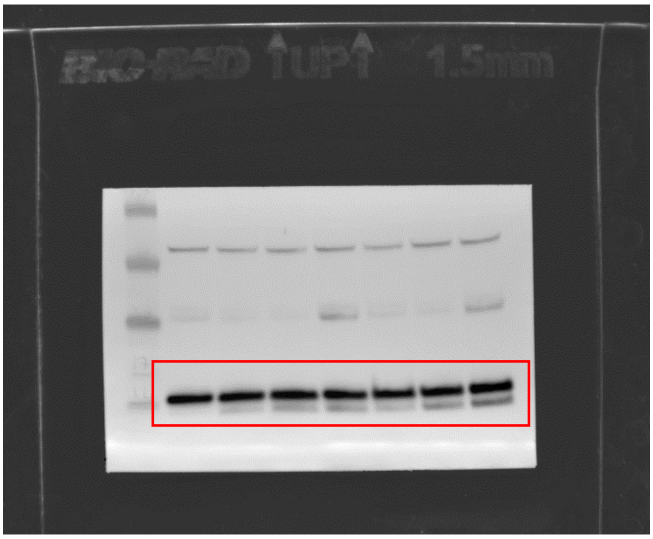

GAPDH – 36 kDa

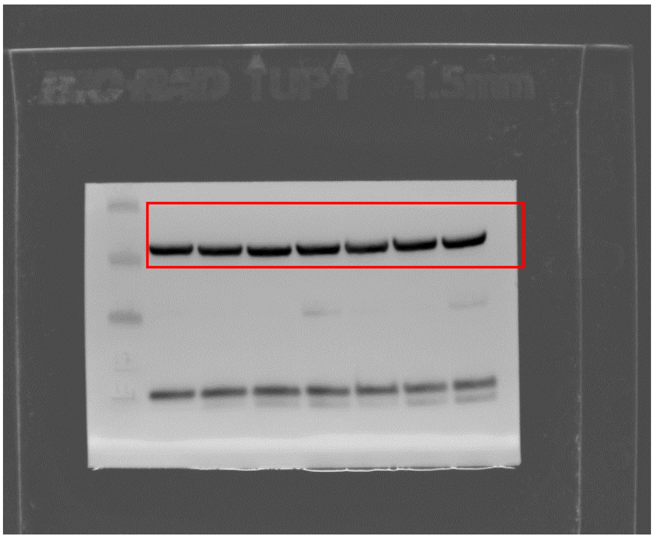

Images showed in Supplementary Fig. S3e

First line: WT untreated, second lane: WT H_2O_2 16h tr., third lane: WT H_2O_2 24h tr., fourth lane: WT CQ 16h tr., fifth lane: WT CQ 16h+ H_2O_2 16h tr., sixth lane: WT CQ 16h+ H_2O_2 24h tr., seventh lane: SCN1A^{mild} untreated, eighth lane: SCN1A^{mild} H_2O_2 16h tr., nineth lane: SCN1A^{mild} H_2O_2 24h tr., tenth lane: SCN1A^{mild} CQ 16h tr., eleventh line: SCN1A^{mild} CQ 16h+ H_2O_2 16h tr., twelfth line: SCN1A^{mild} CQ 16h+ H_2O_2 24h tr.

p62 – 62 kDa (1° replicate)

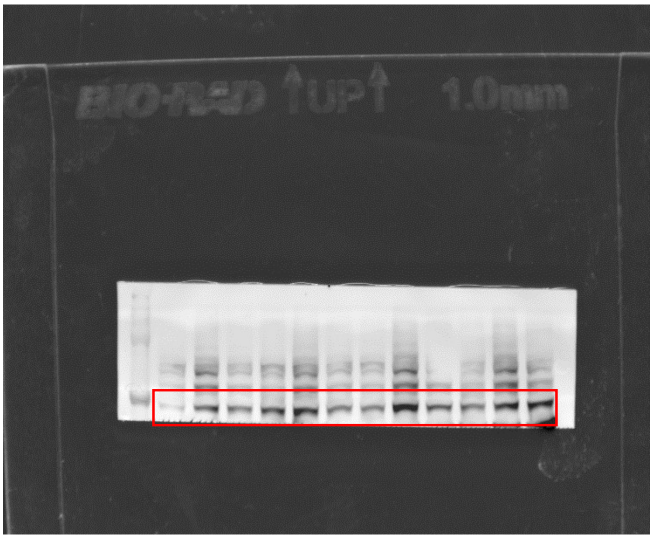

LC3I-II – 14 kDa (1° replicate)

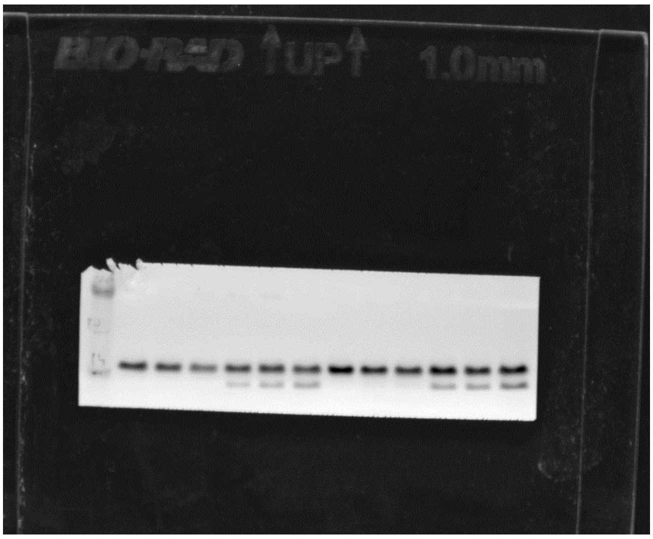

GAPDH – 36 kDa (1° replicate)

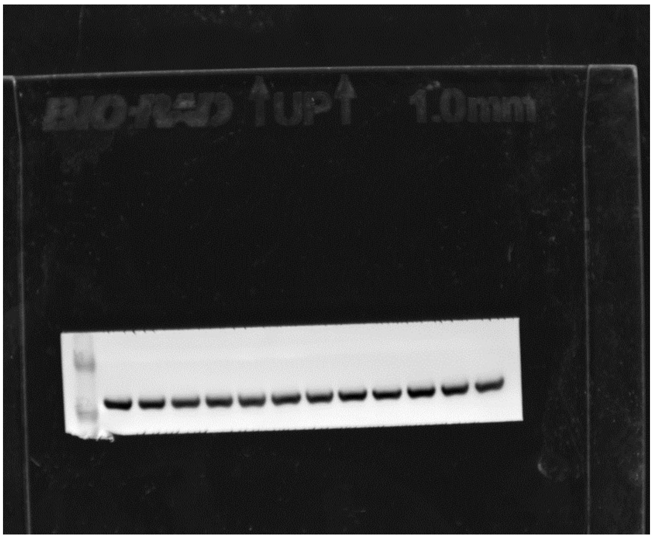

p62 (2° replicate)

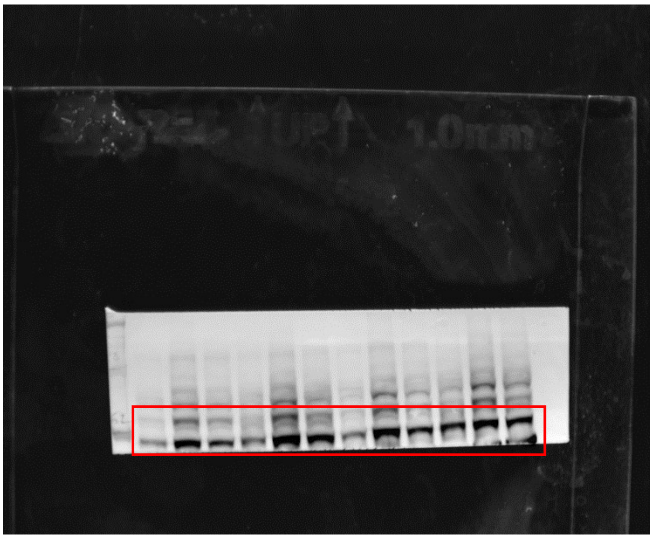

LC3I-II (2° replicate)

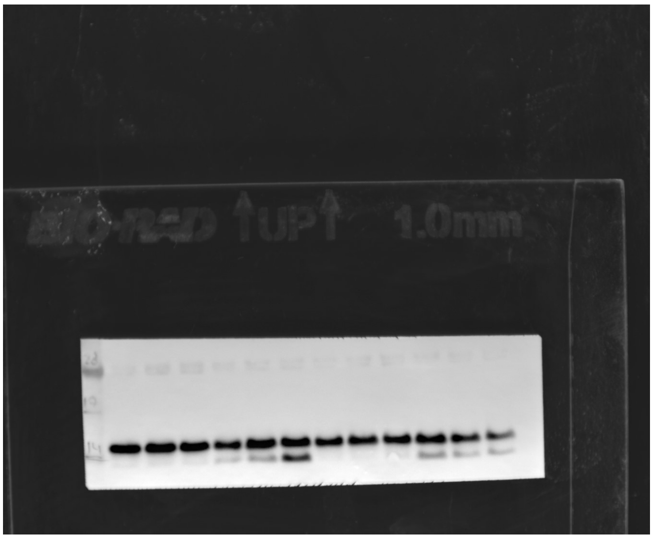

GAPDH (3° replicate)

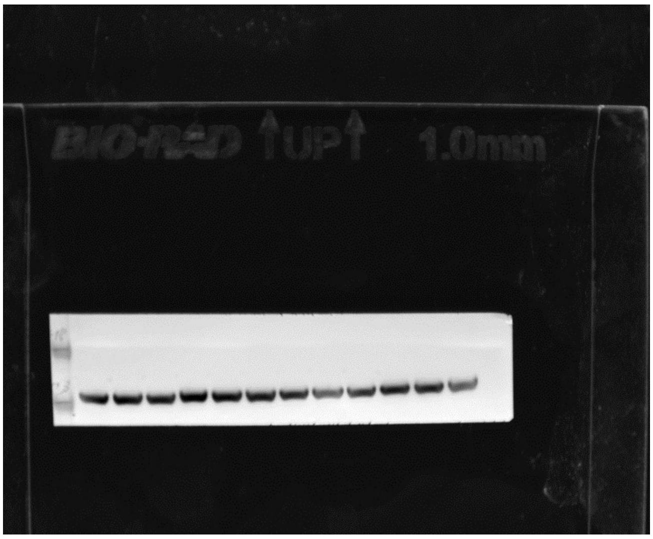

Images showed in Supplementary Fig. S3i

First line: WT untreated, second lane: WT 4h CQ 100uM tr., third lane: WT 8h CQ 100uM tr., fourth lane: WT 16h CQ 100uM tr., fifth lane: WT 24h CQ 100uM tr.

LC3I-II – 14 kDa (1° replicate)

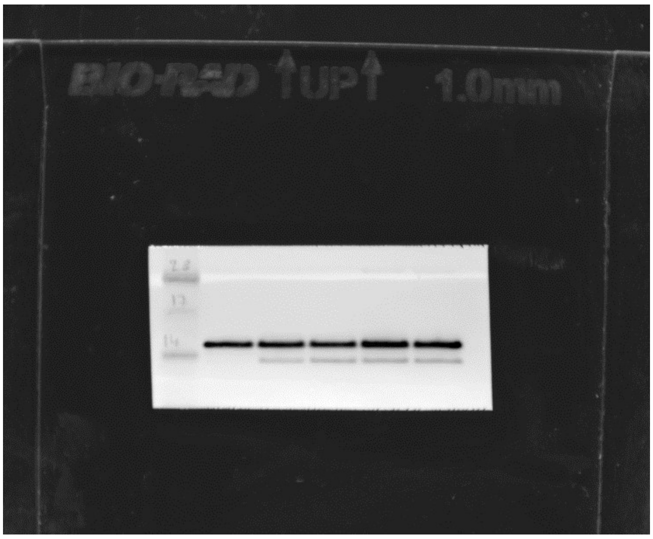

GAPDH – 36 kDa (1° replicate)

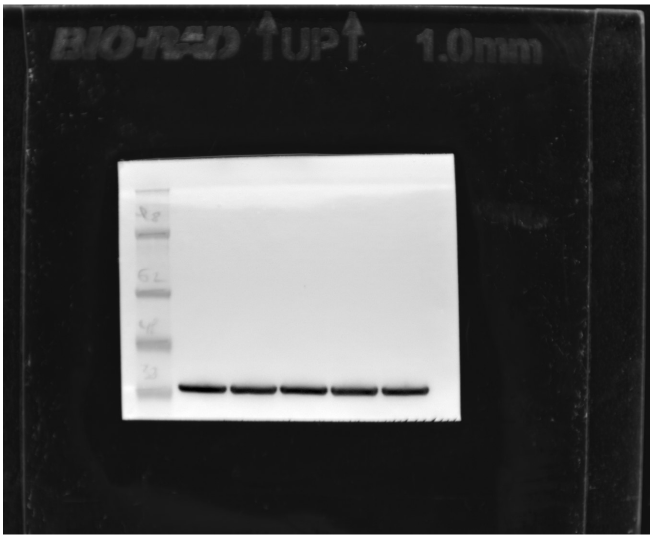

LC3I-II (2° replicate)

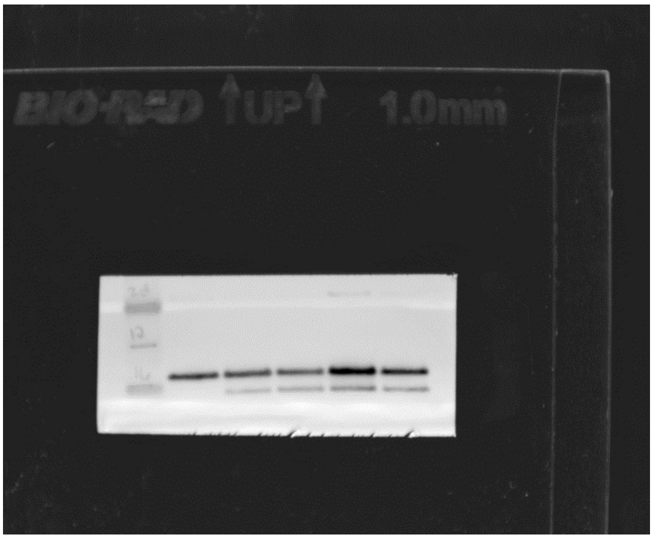

GAPDH (2° replicate)

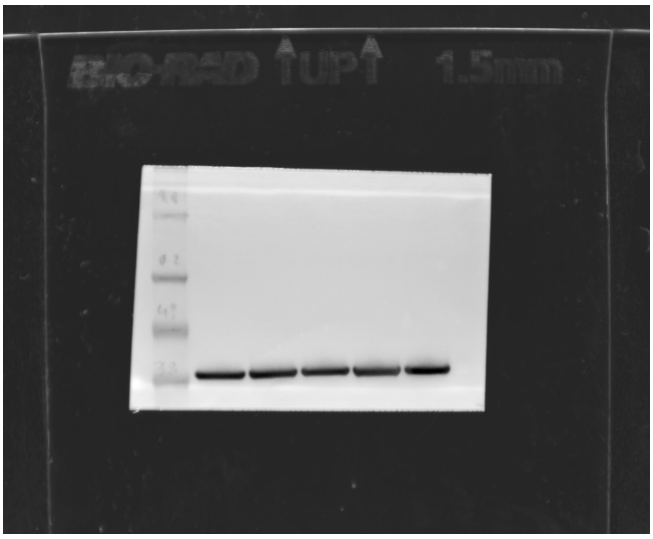

First line: SCN1A^{severe} untreated, second lane: SCN1A^{severe} 4h CQ 100uM tr., third lane: SCN1A^{severe} 8h CQ 100uM tr., fourth lane: SCN1A^{severe} 16h CQ 100uM tr., fifth lane: SCN1A^{severe} 24h CQ 100uM tr.

LC3I-II – 14 kDa (1° replicate)

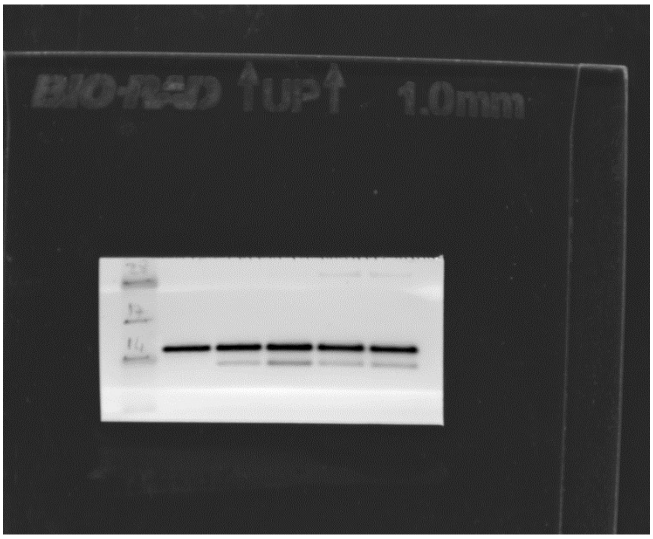

GAPDH – 36 kDa (1° replicate)

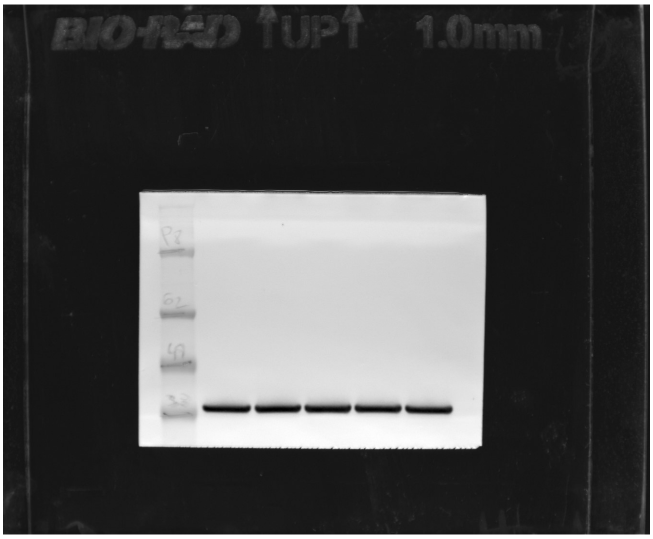

LC3I-II (2° replicate)

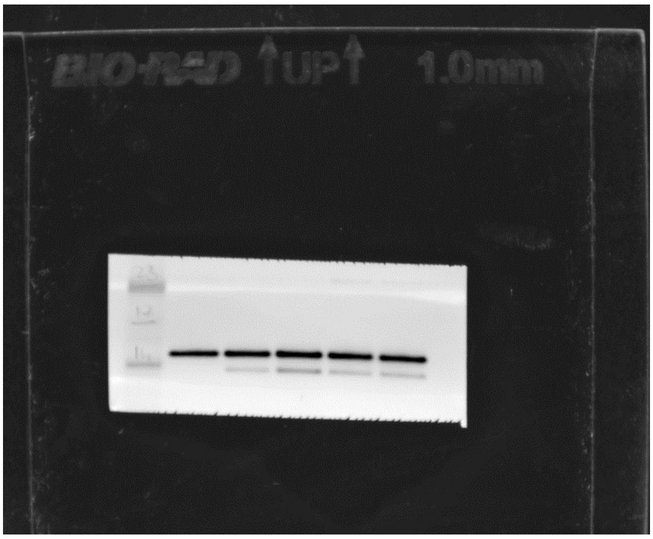

GAPDH (2° replicate)

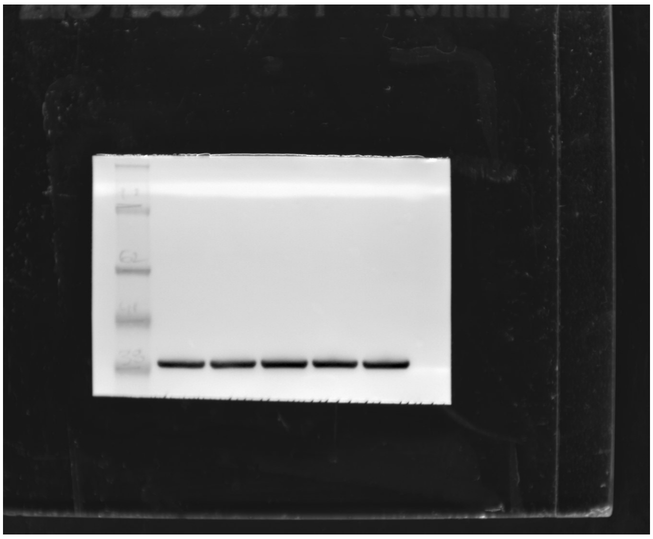

First line: SCN1A^{mild} untreated, second lane: SCN1A^{mild} 4h CQ 100uM tr., third lane: SCN1A^{mild} 8h CQ 100uM tr., fourth lane: SCN1A^{mild} 16h CQ 100uM tr., fifth lane: SCN1A^{mild} 24h CQ 100uM tr.

LC3I-II – 14 kDa (1° replicate)

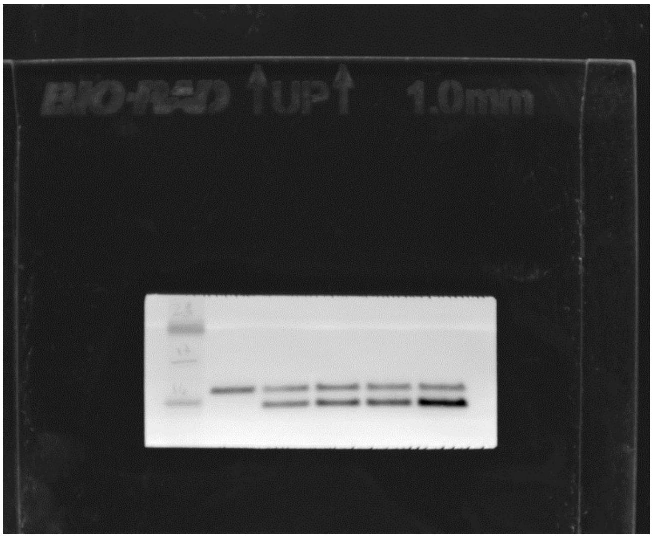

GAPDH – 36 kDa (1° replicate)

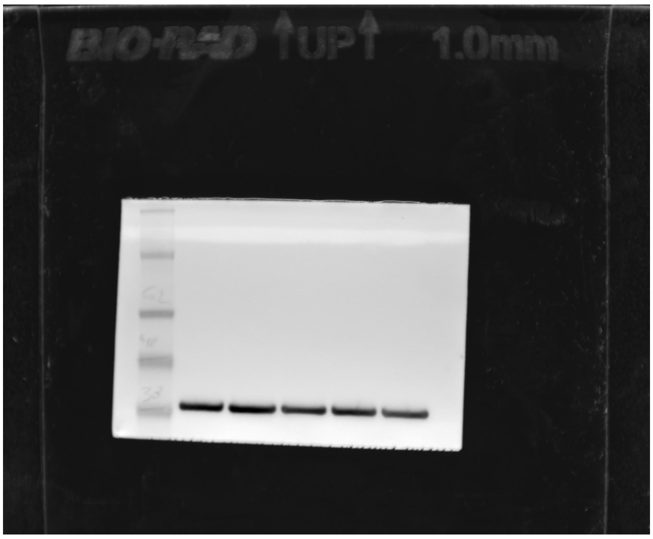

LC3I-II (2° replicate)

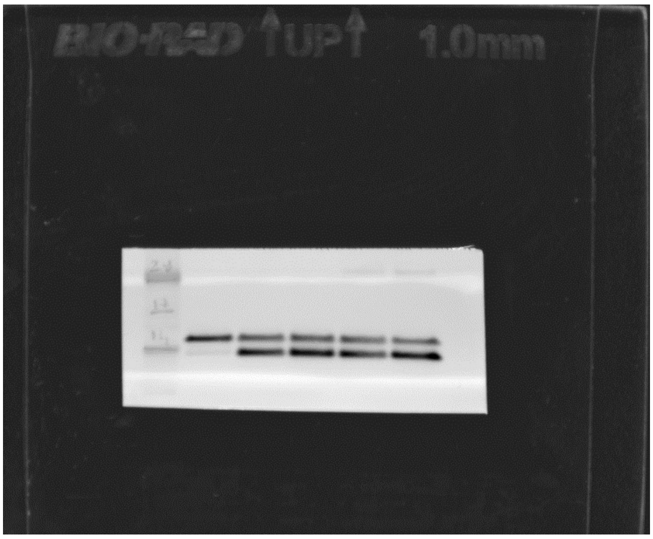

GAPDH (2° replicate)

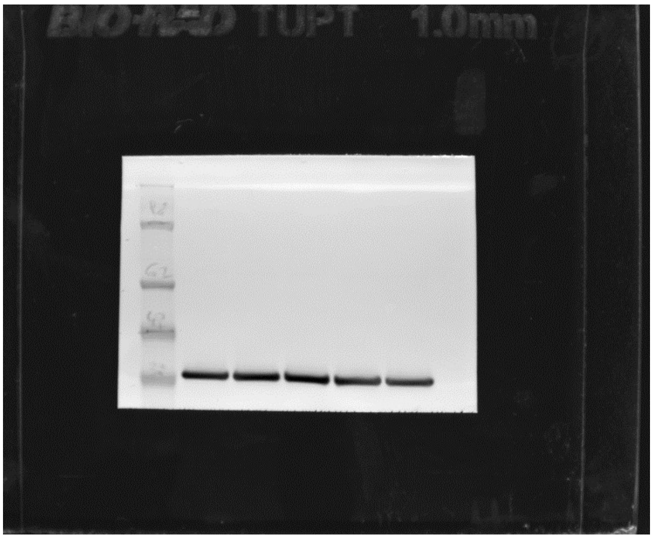

Images showed in Supplementary Fig. S4a

First line: WT untreated, second lane: WT 30’ H_2O_2 1mM tr., third lane: WT 60’ H_2O_2 1mM tr., fourth lane: WT 30’ H_2O_2 2mM tr., fifth lane: WT 60’ H_2O_2 2mM tr.

pHSPB1 – 27 kDa (1° replicate)

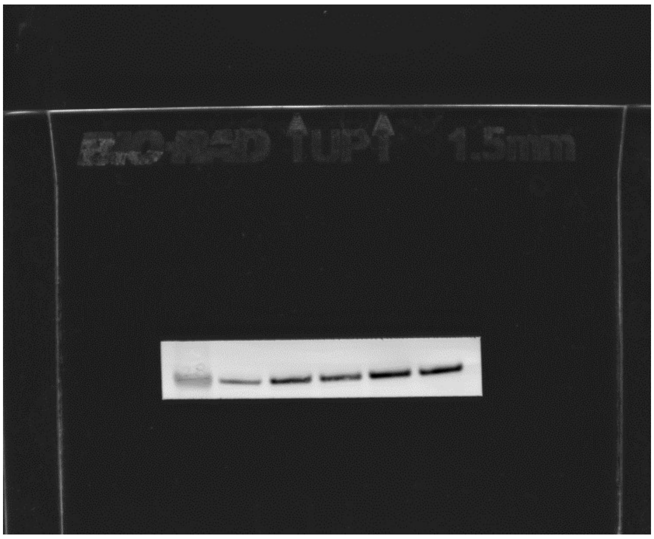

HSPB1 – 27 kDa (1° replicate)

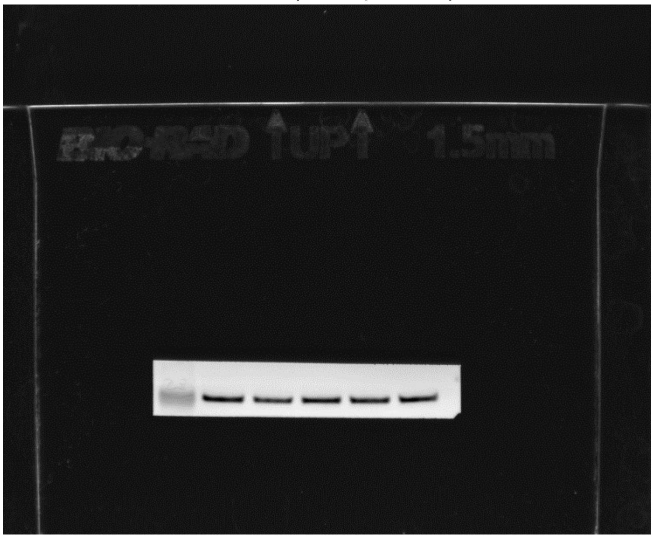

p-p38 – 38 kDa (1° replicate)

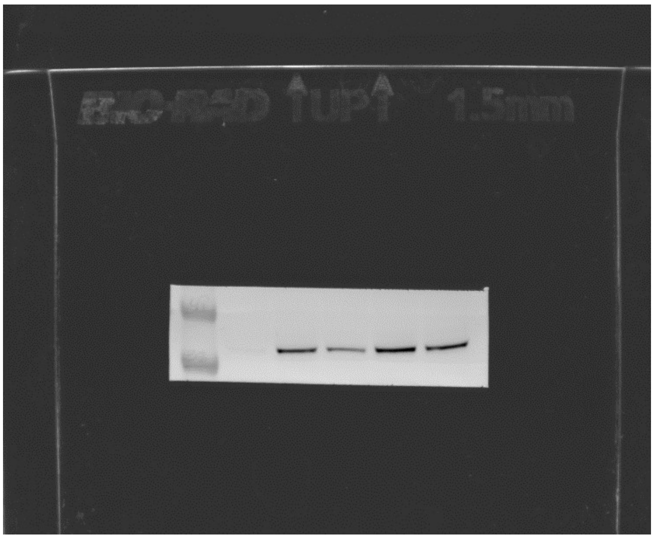

p38 – 38 kDa (1° replicate)

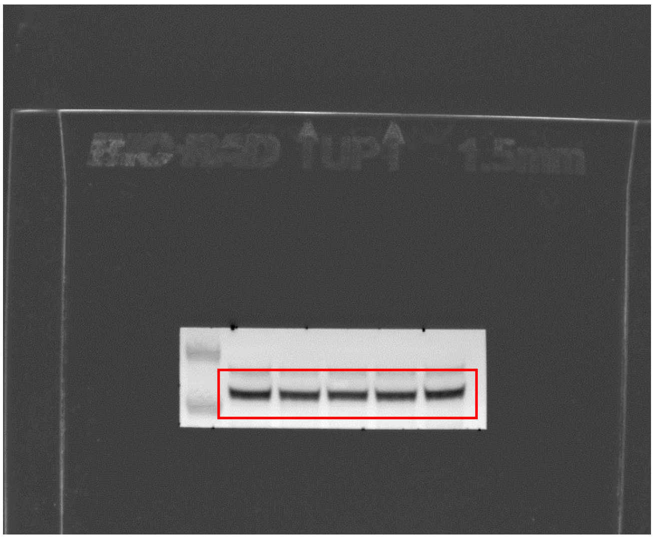

H3 – 17 kDa (1° replicate)

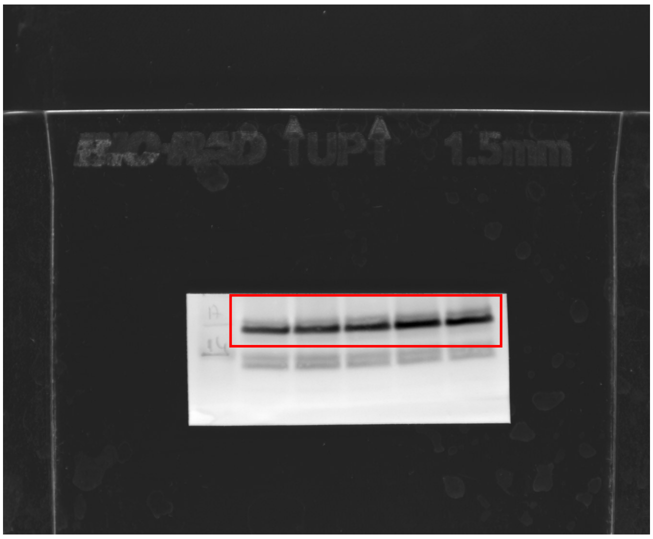

H3 – 17 kDa (1° replicate)

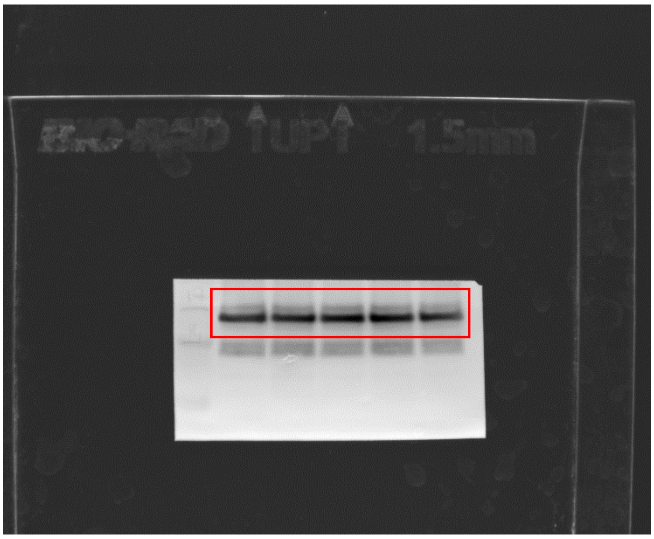

pHSPB1 (2° replicate)

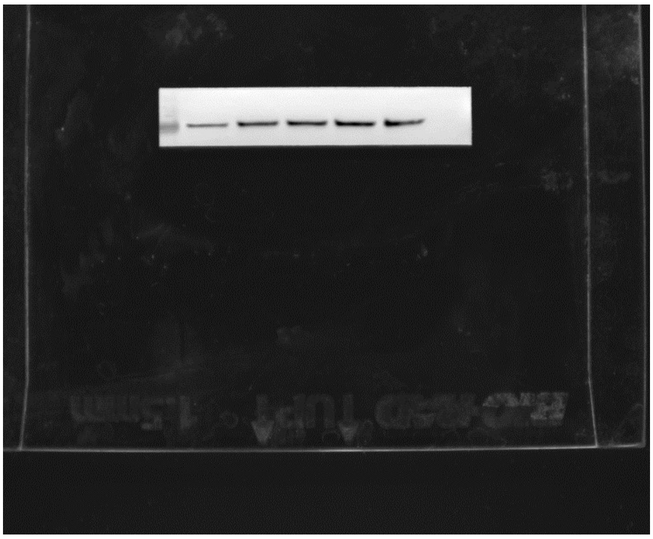

HSPB1 (2° replicate)

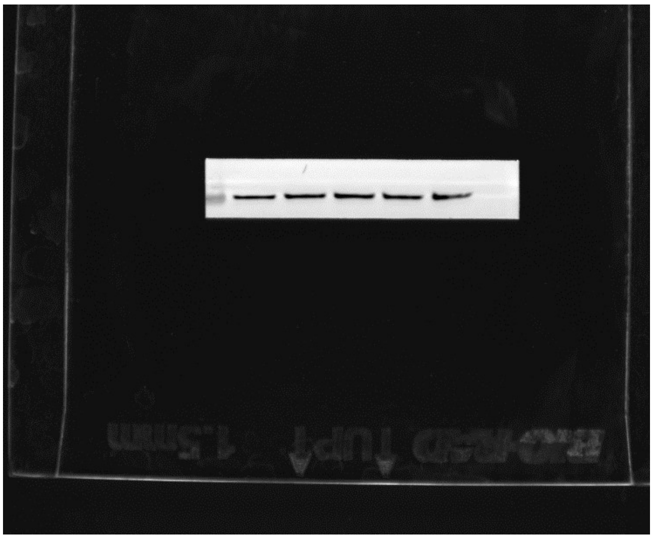

p-p38 (2° replicate)

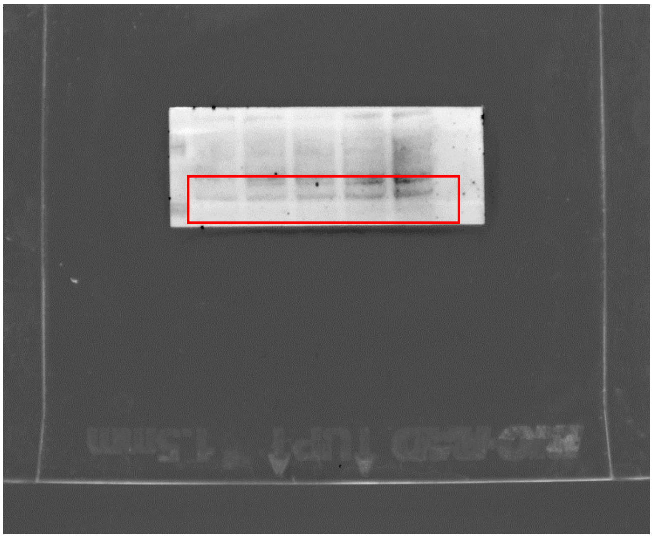

p38 (2° replicate)

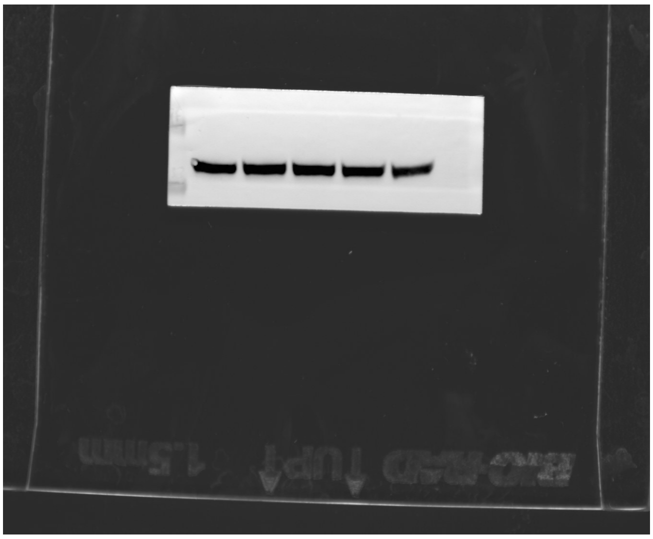

H3 (2° replicate)

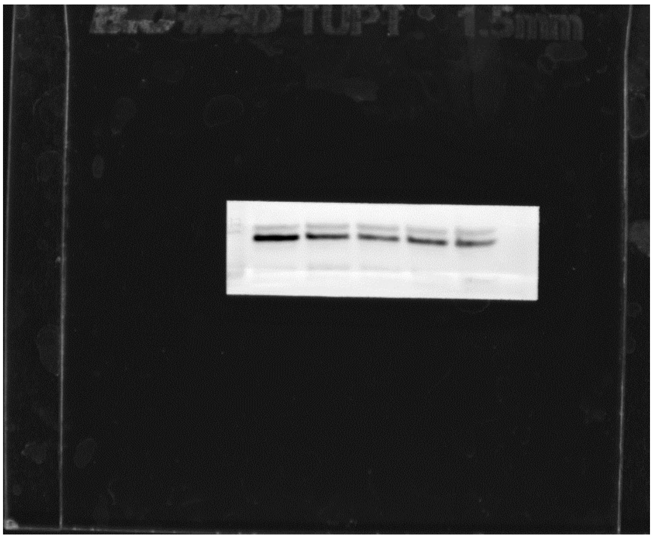

pHSPB1 (3° replicate)

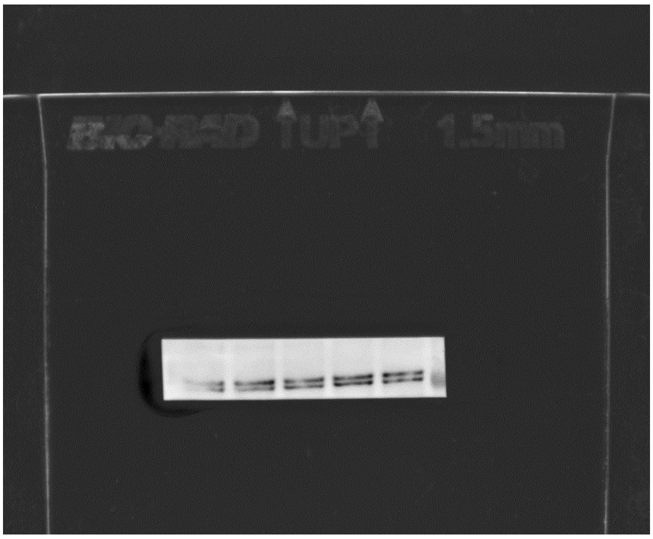

HSPB1 (3° replicate)

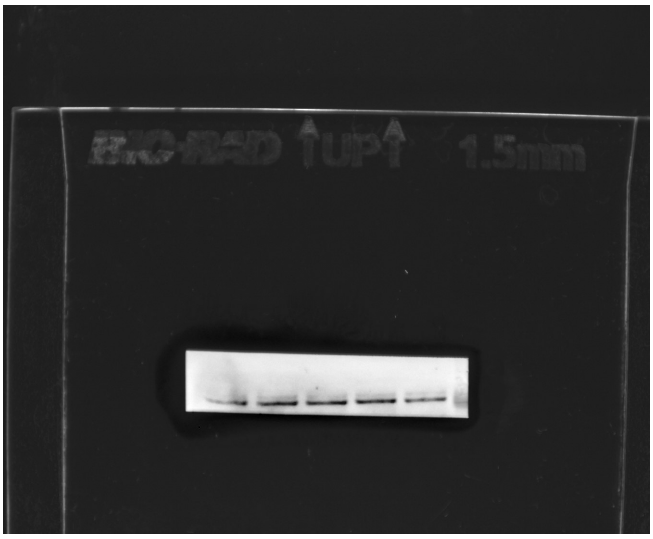

p-p38 (3° replicate)

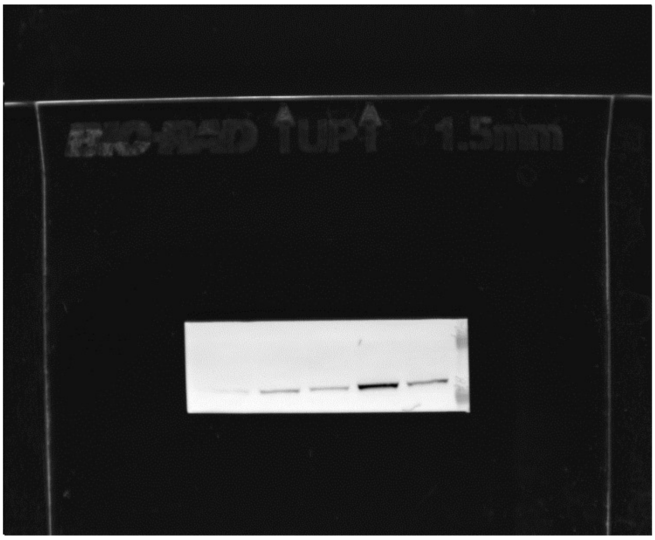

p38 (3° replicate)

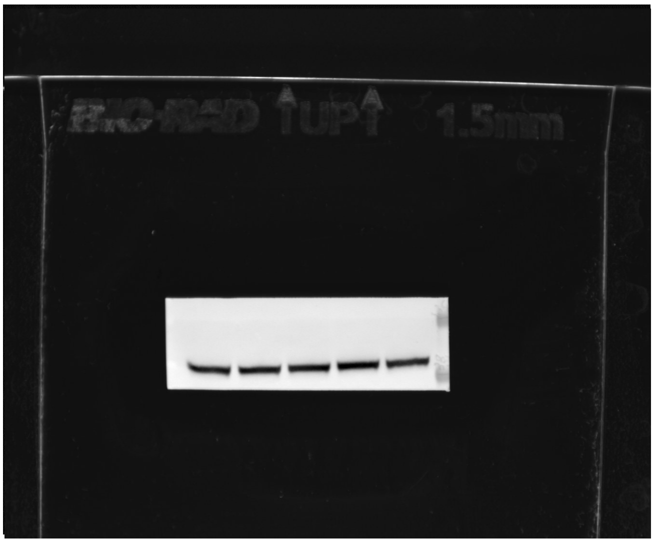

H3 (3° replicate)

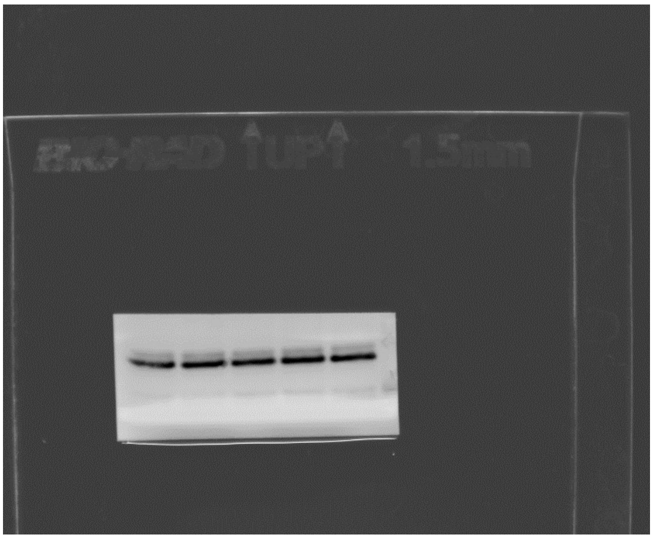

Images showed in FIG. Supplementary 4c

First line: WT untreated, second lane: WT 30’ H2O2 1mM tr., third lane: WT 30’ H2O2 2mM tr., fourth lane: WT 30’ SB 10uM tr., fifth lane: WT 30’ SB 10uM+H2O2 1mM tr., sixth lane: WT 30’ SB 10uM+H2O2 2mM tr.

pHSPB1 – 27 kDa (1° replicate)

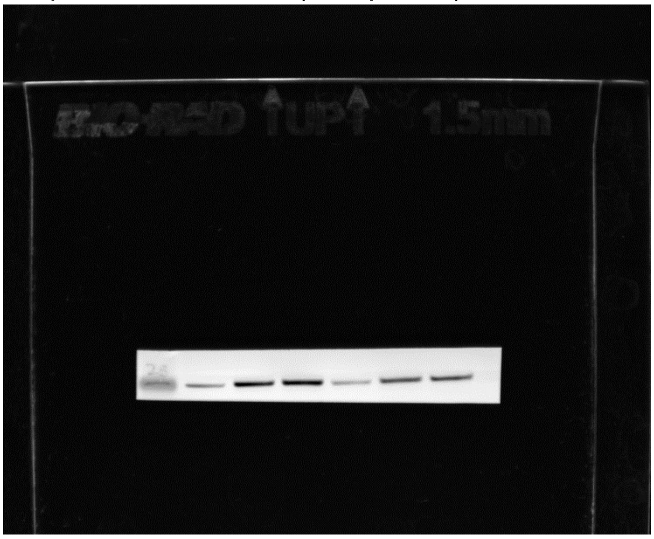

HSPB1 – 27 kDa (1° replicate)

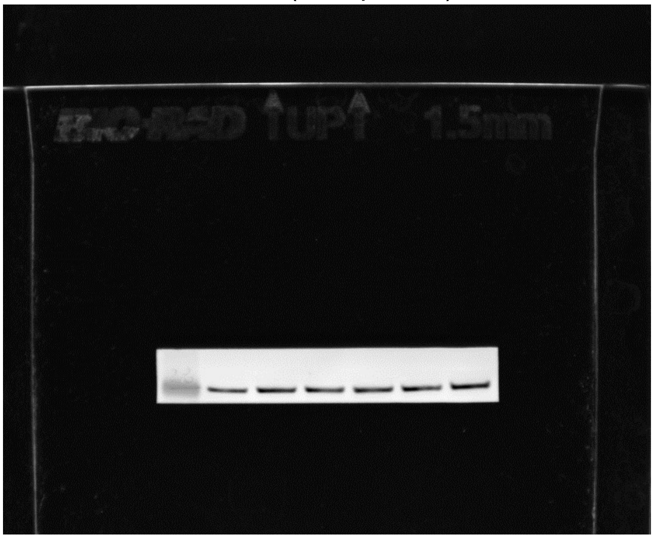

p-p38 – 38 kDa (1° replicate)

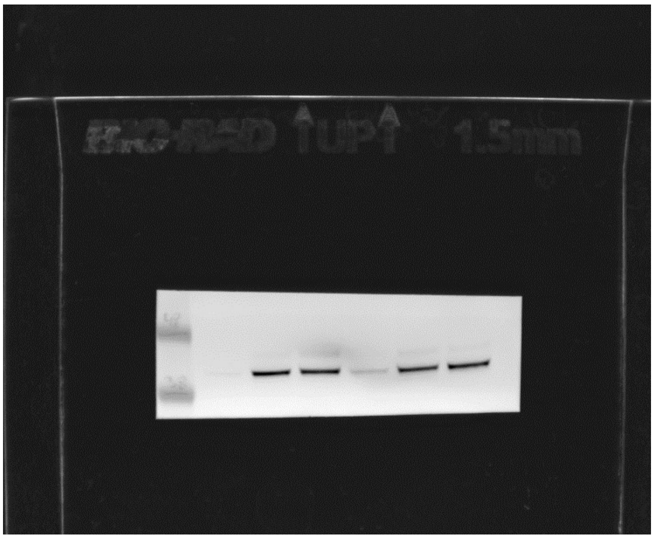

p38 – 38 kDa (1°replicate)

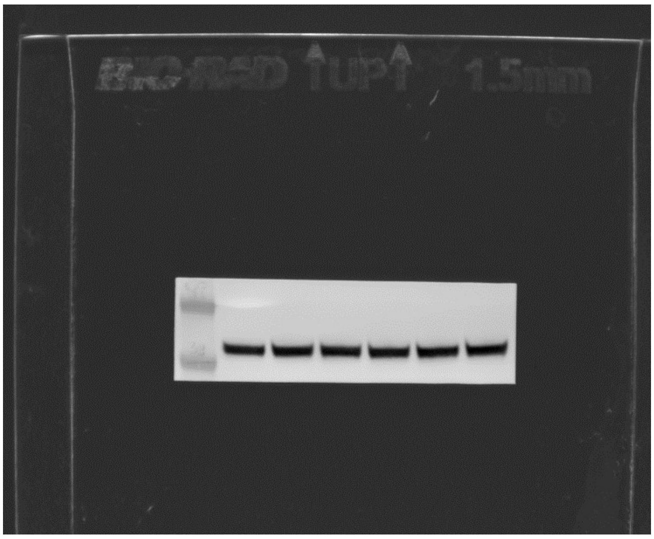

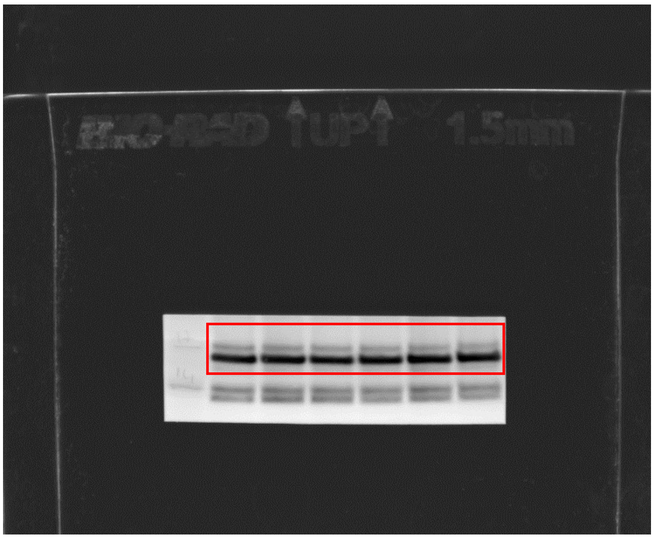

H3 – 17 kDa (1° replicate)

pHSPB1 (2° replicate)

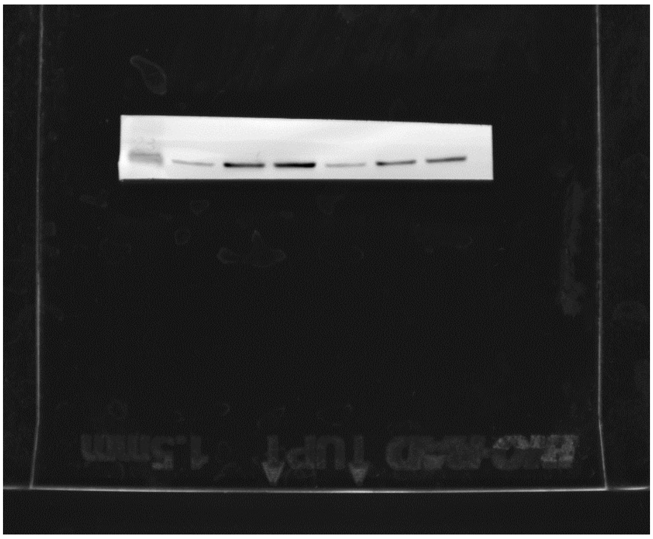

HSPB1 (2° replicate)

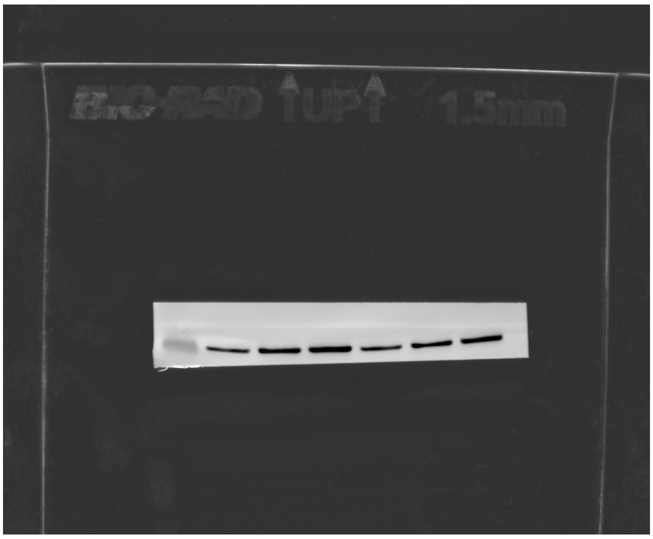

H3 (2° replicate)

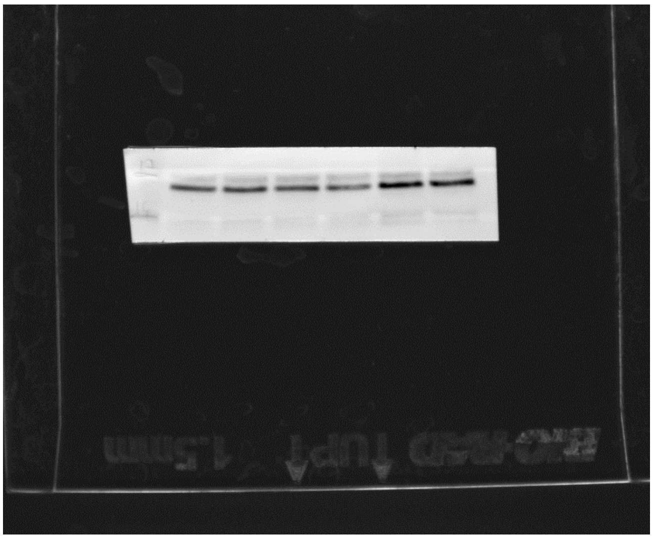

p-p38 (2° replicate)

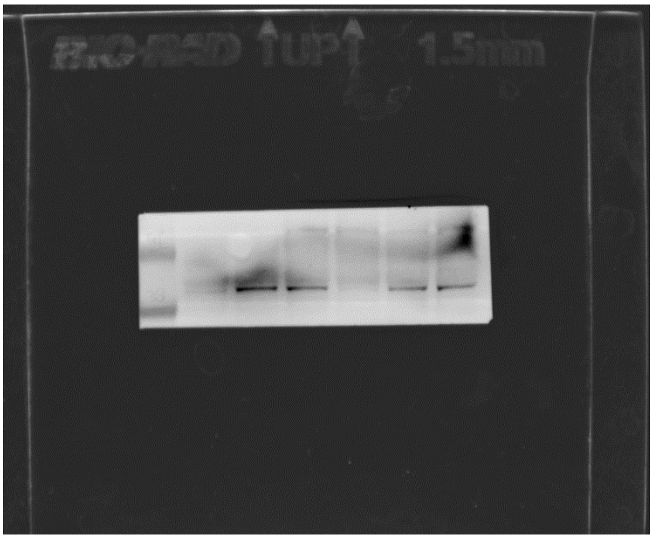

p38 (2° replicate)

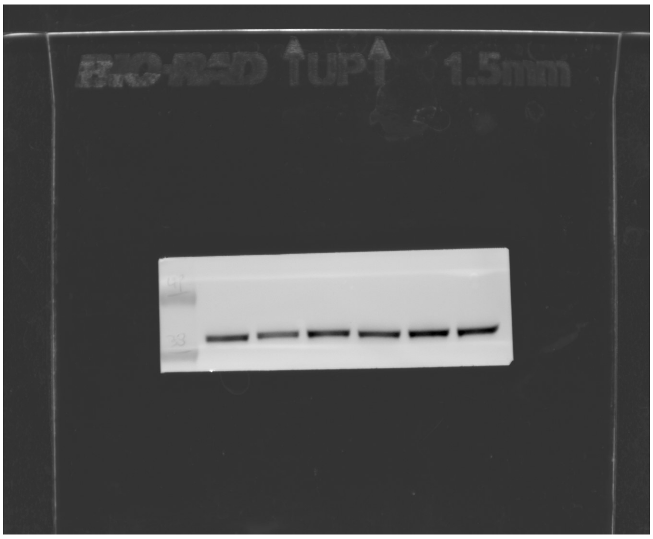

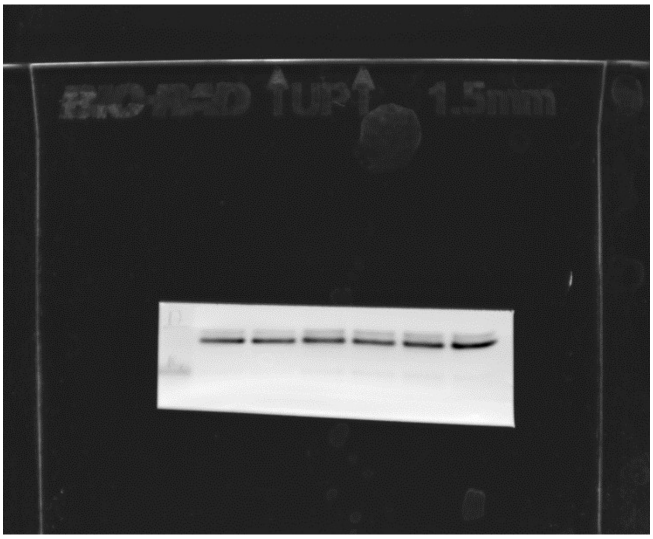

H3 (2° replicate)

pHSPB1 (3° replicate)

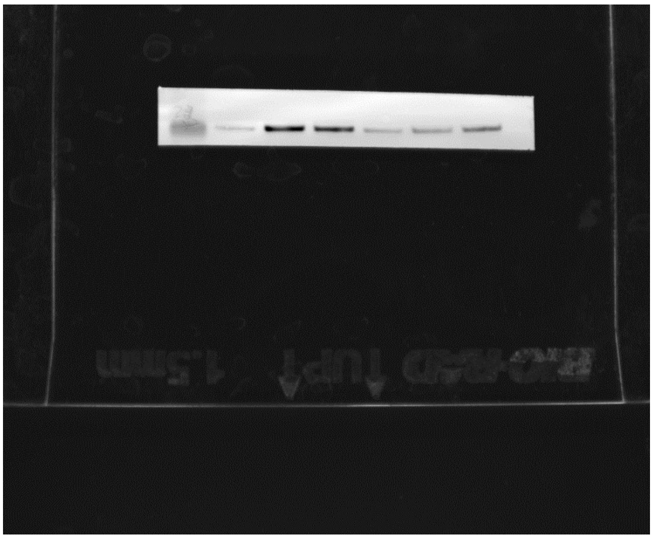

HSPB1 (3° replicate)

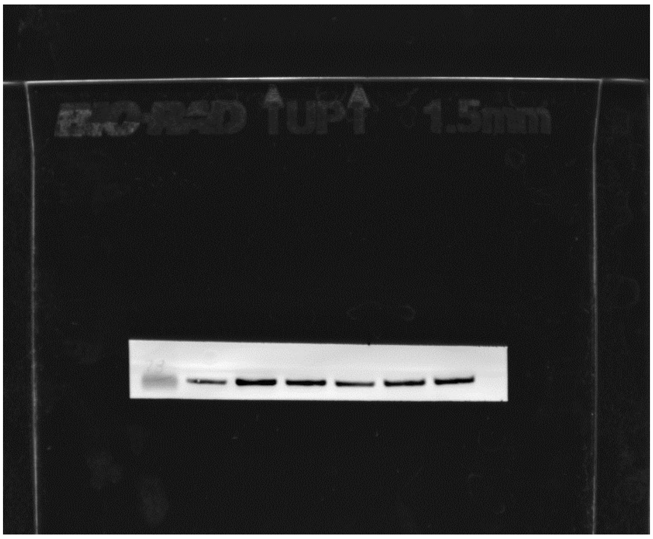

H3 (3° replicate)

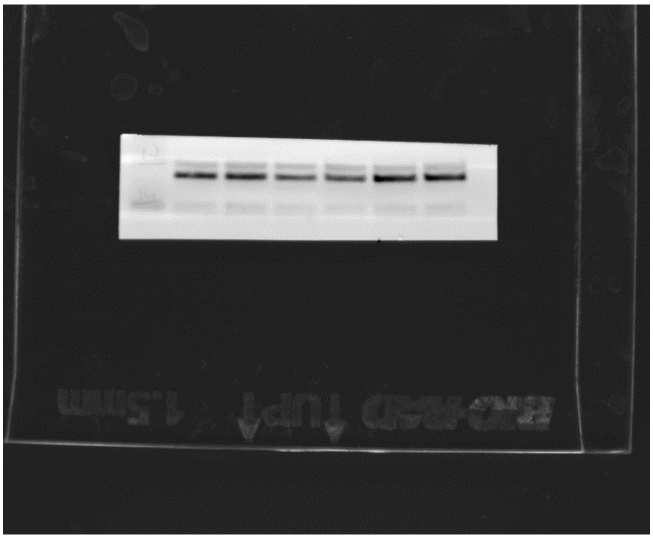

p-p38 (3° replicate)

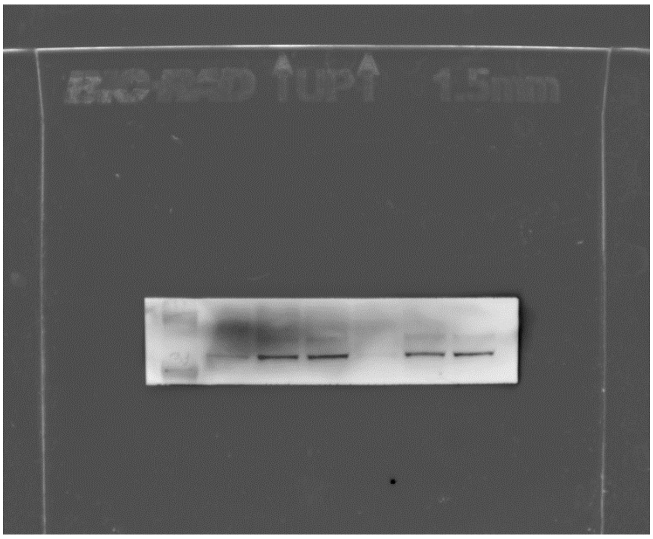

p38 (3° replicate)

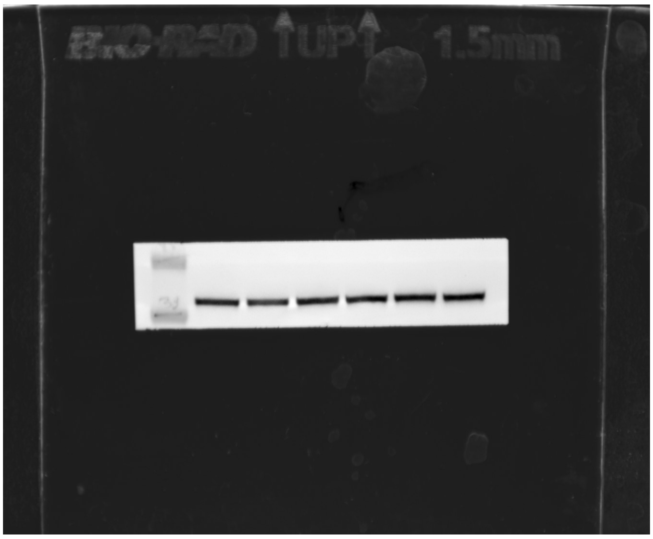

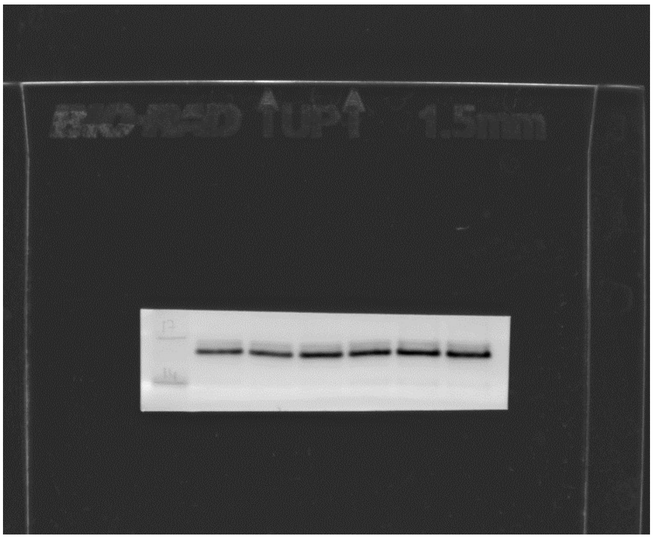

H3 (3° replicate)

Images showed in FIG. Supplementary 4e

First line: WT untreated, second lane: WT 8h tr., third lane: WT 16h tr., fourth lane: WT 24h tr.

p-p38 – 38 kDa (1° replicate)

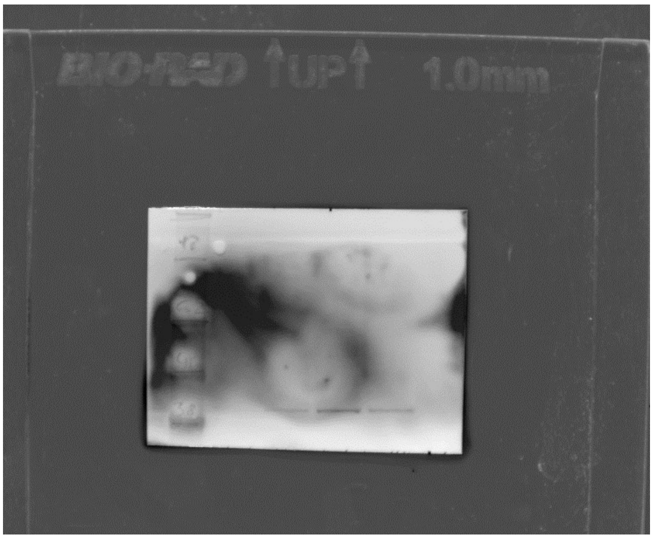

p38 – 38 kDa (1°replicate)

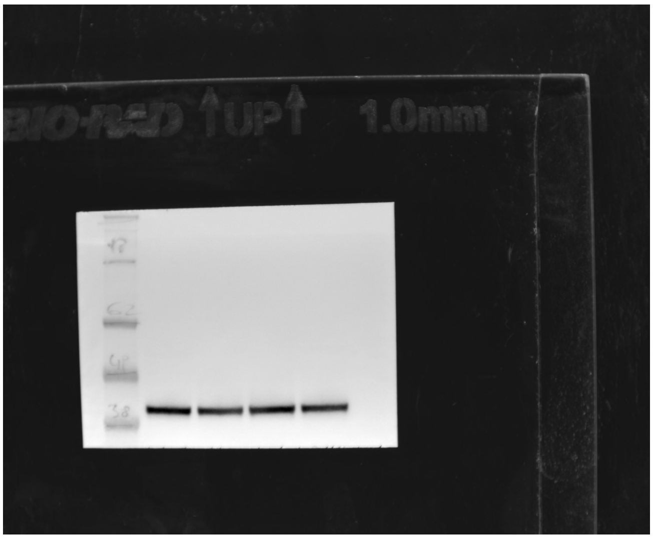

pHSPB1 – 27 kDa (1° replicate)

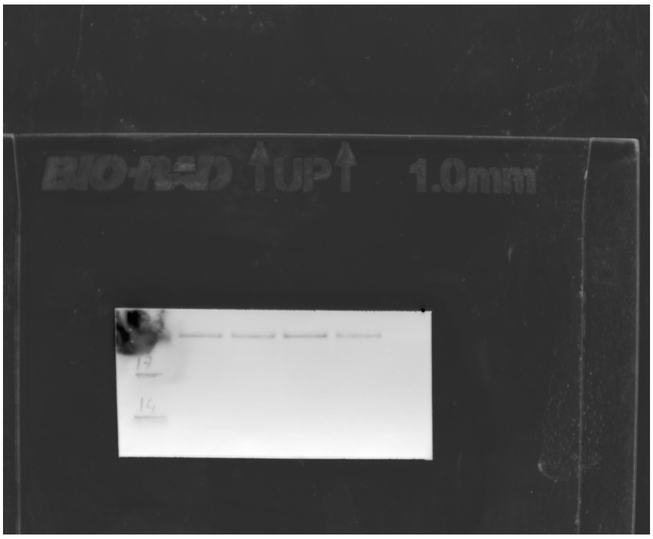

HSPB1 – 27 kDa (1° replicate)

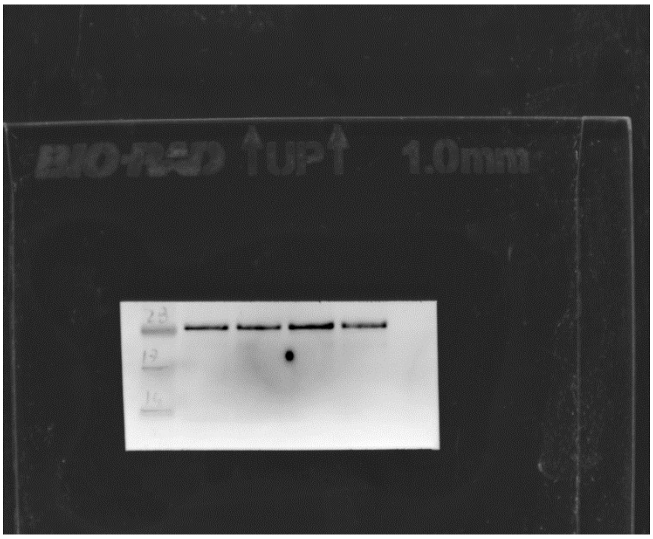

γ-tubulin – 50 kDa (1° replicate)

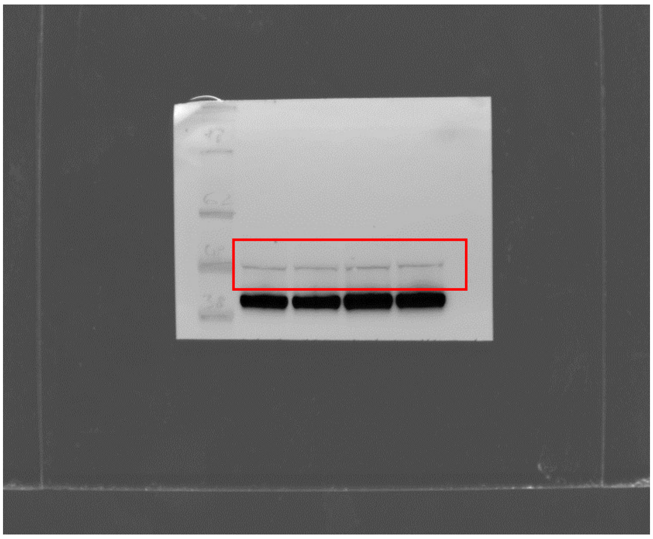

p-p38 (2° replicate)

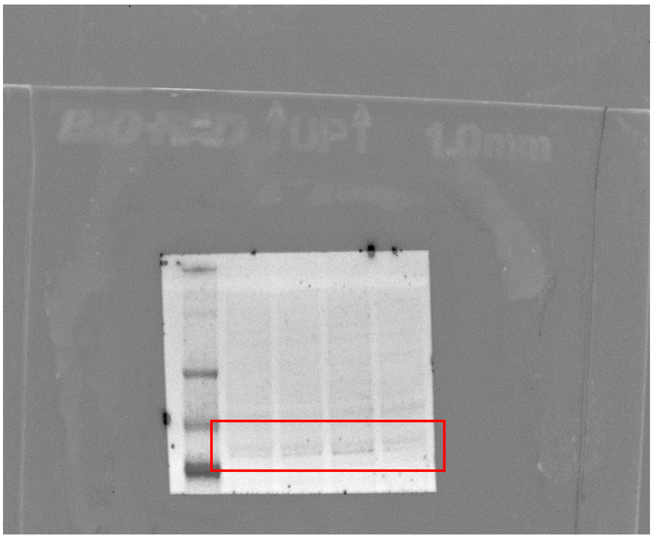

p38 (2° replicate)

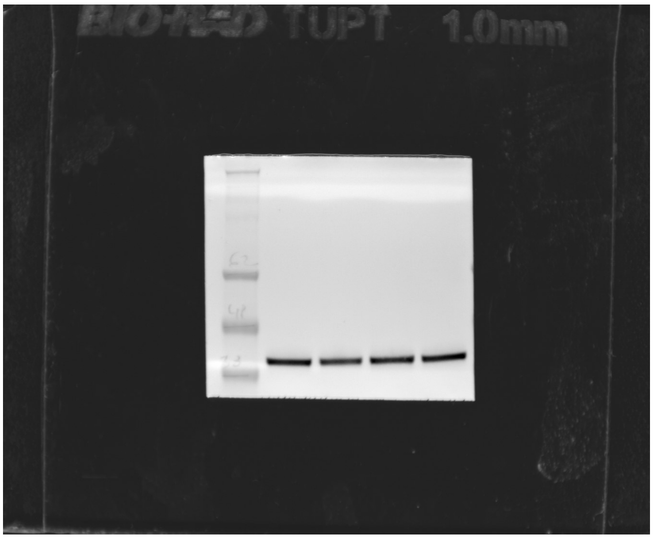

pHSPB1 (2° replicate)

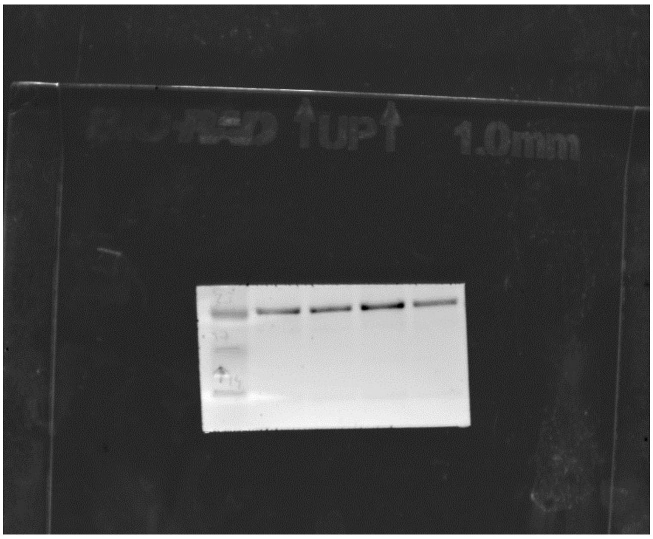

HSPB1 (2° replicate)

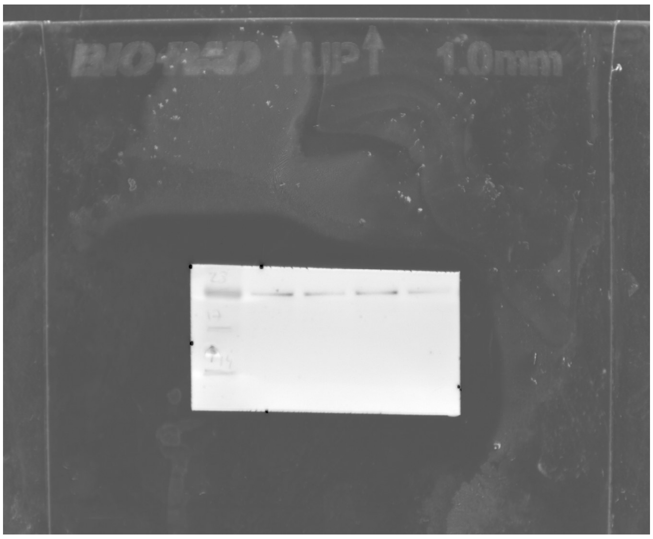

γ-tubulin (2° replicate)

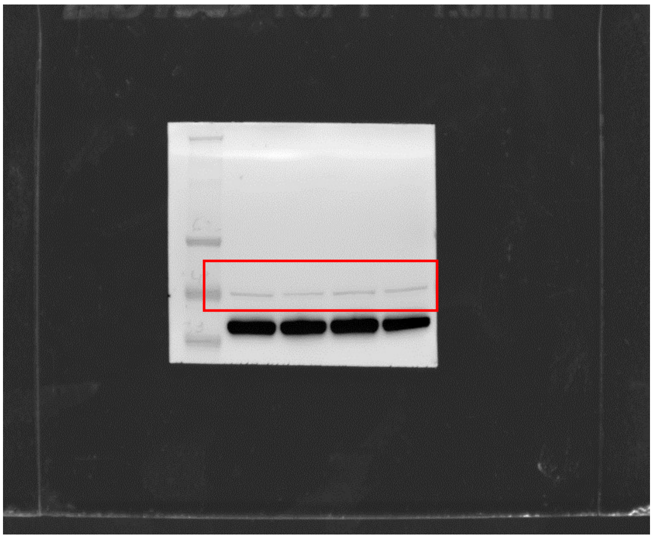

p-p38 (3° replicate)

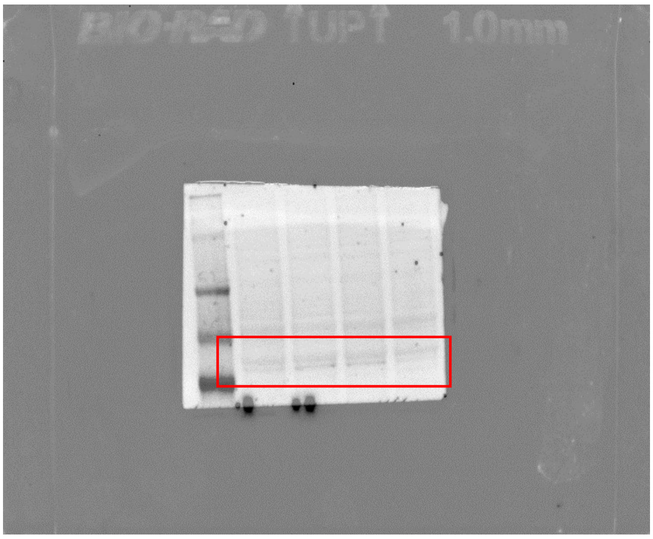

p38 (3° replicate)

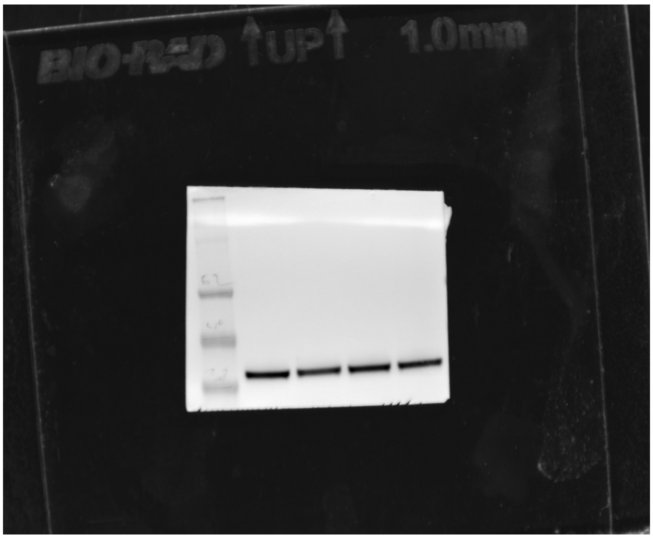

pHSPB1 (3° replicate)

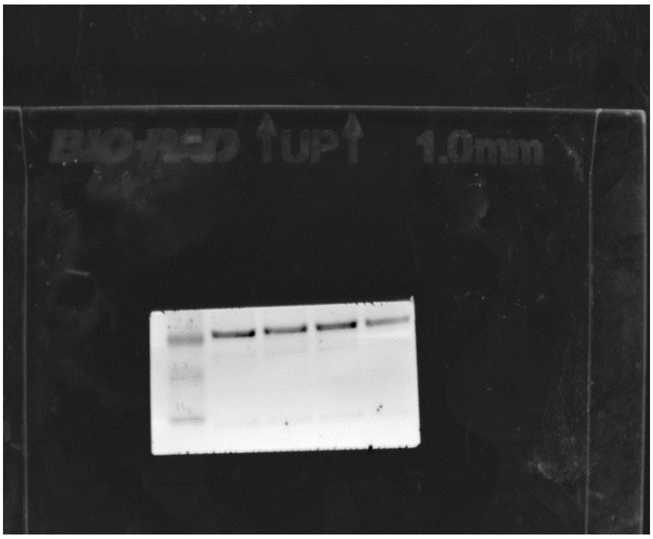

HSPB1 (3° replicate)

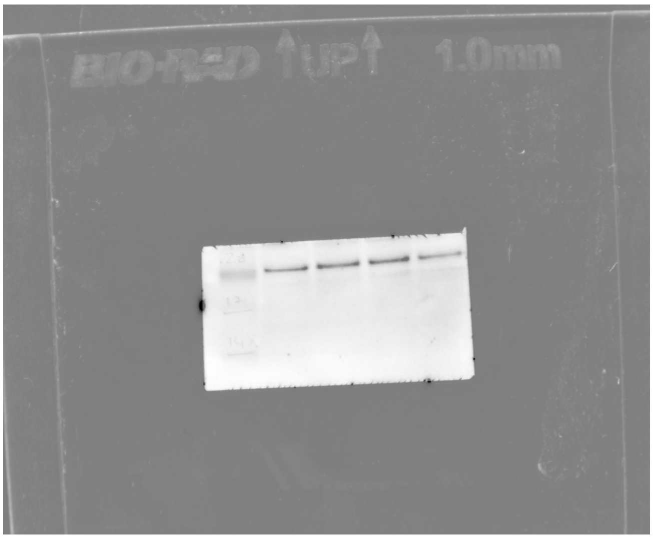

γ-tubulin (3° replicate)

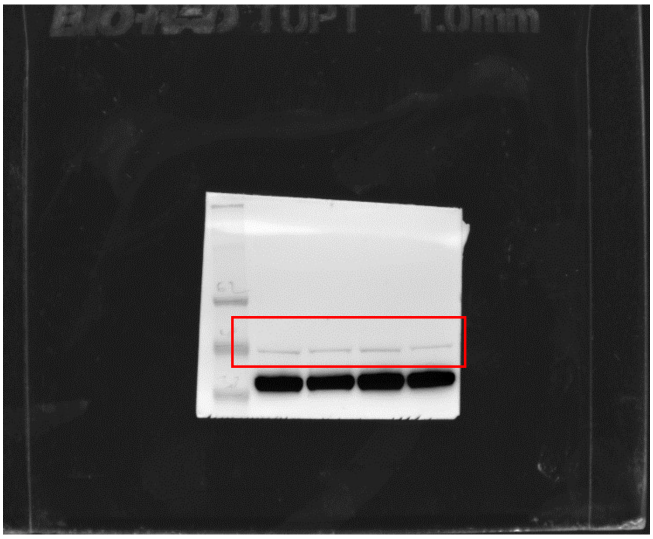

Images showed in FIG. Supplementary 5a

First line: WT, second lane: SCN1A^{mild}

HSPB1 – 27 kDa (1° replicate)

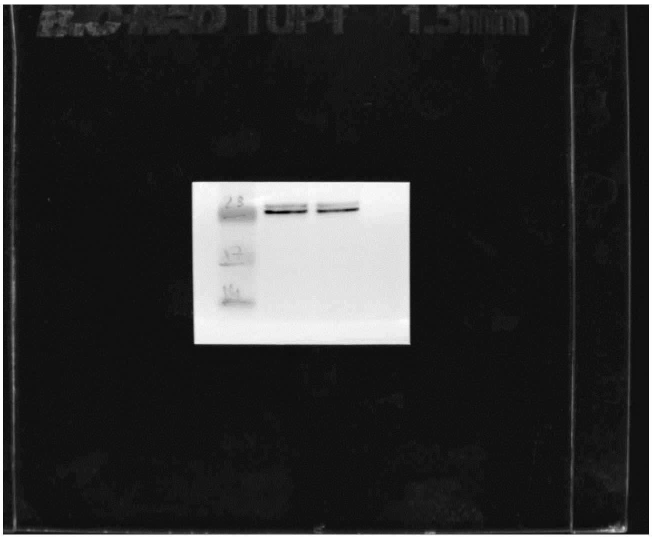

GAPDH – 36 kDa (1° replicate)

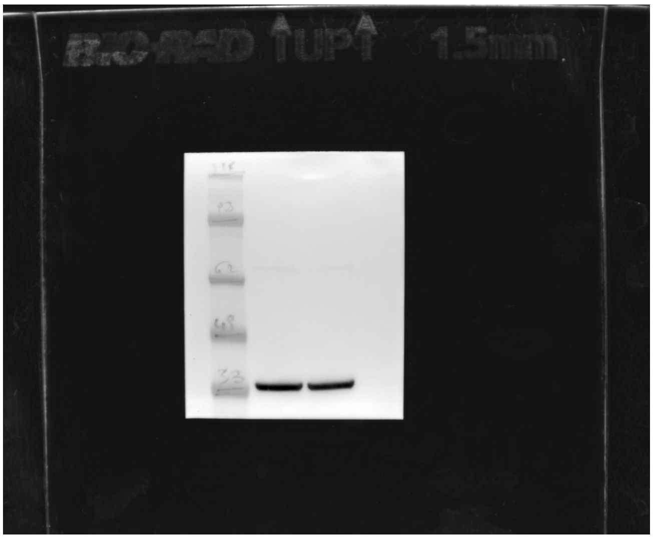

HSPB1 (2° replicate)

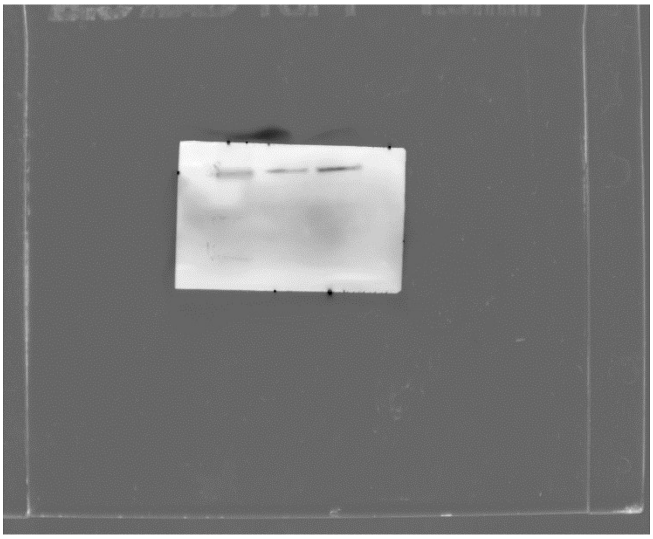

GAPDH (2° replicate)

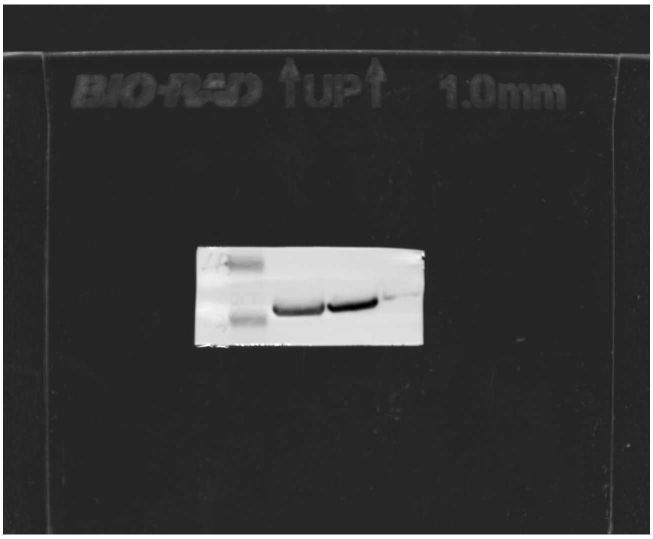

HSPB1 (3° replicate)

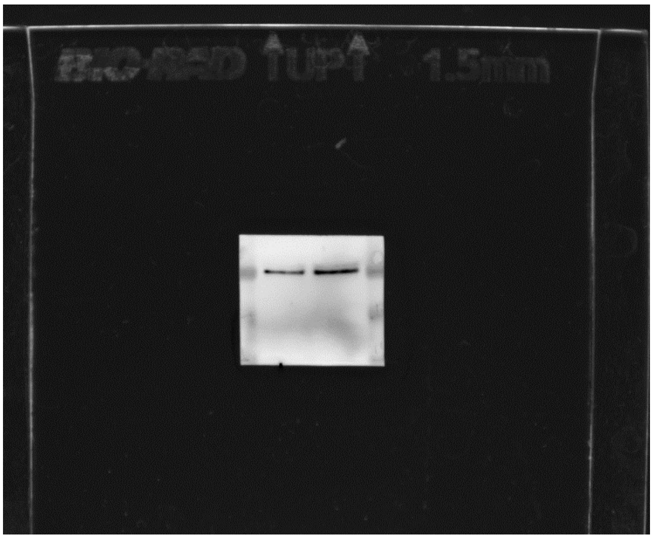

GAPDH (3° replicate)

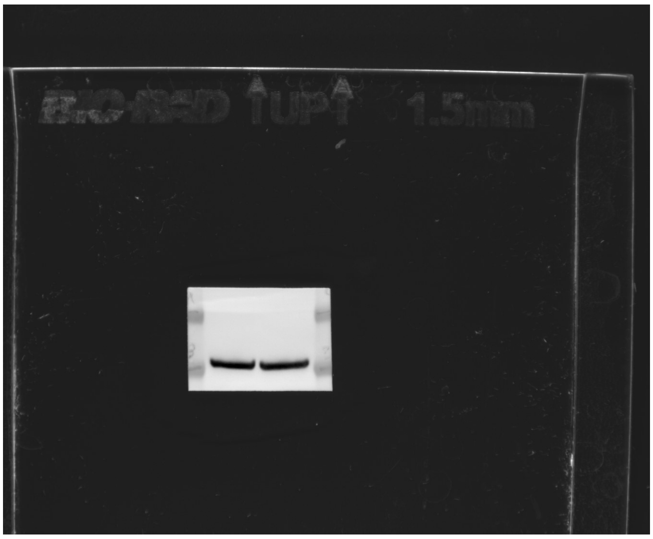

Images showed in FIG. Supplementary 5

First line: WT, second lane: SCN1A^{severe}

HSPA8 – 71 kDa (1° replicate)

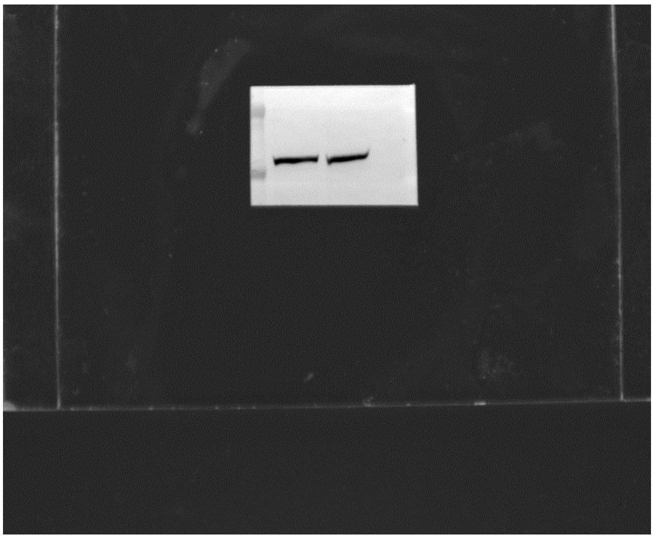

GAPDH – 36 kDa (1° replicate)

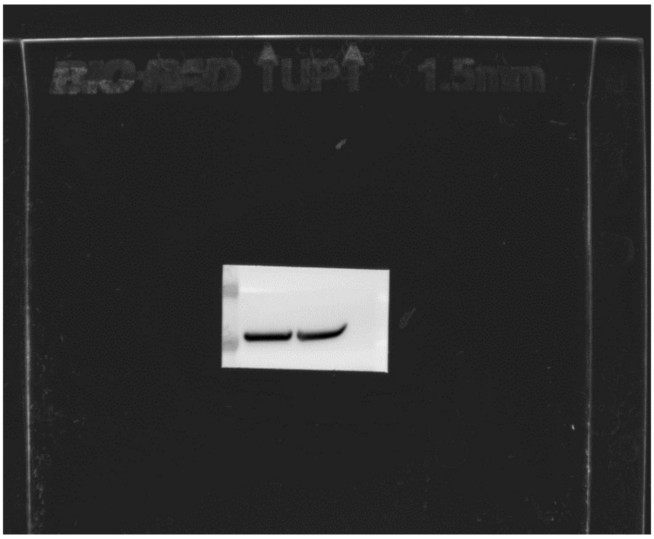

HSPA8 (2° replicate)

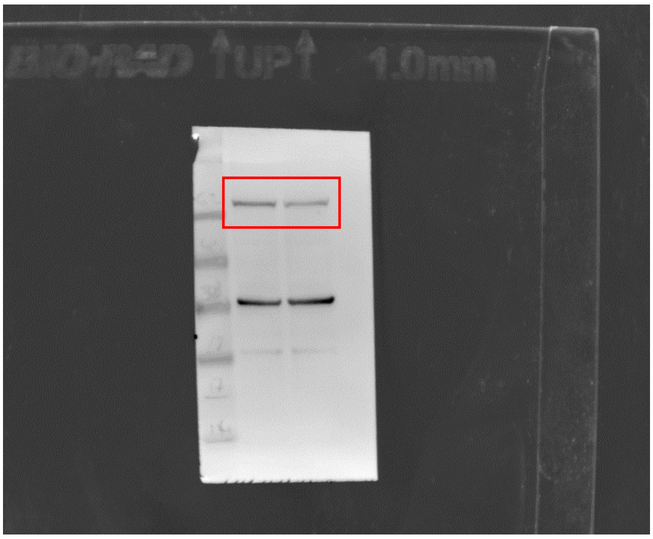

GAPDH (2° replicate)

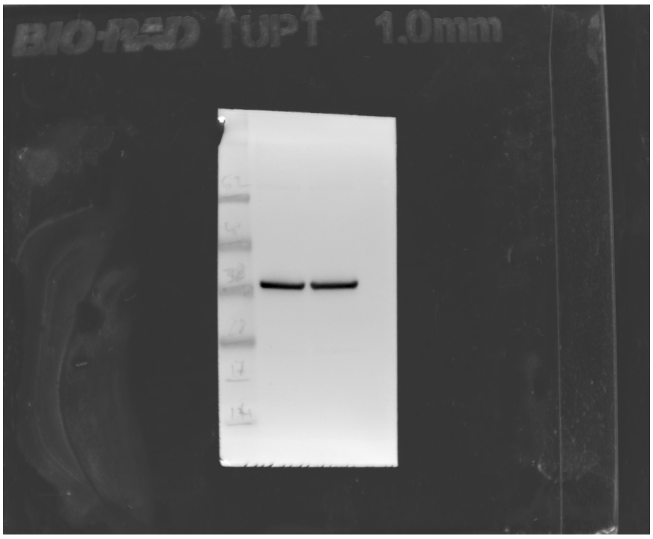

HSPA8 (3° replicate)

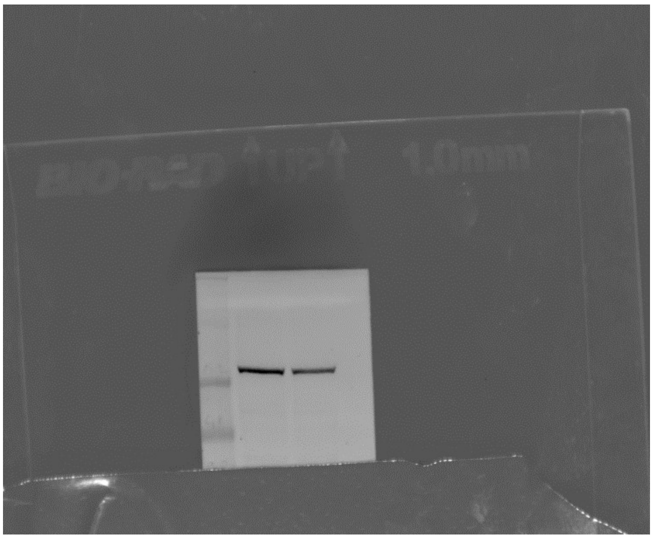

GAPDH (3° replicate)

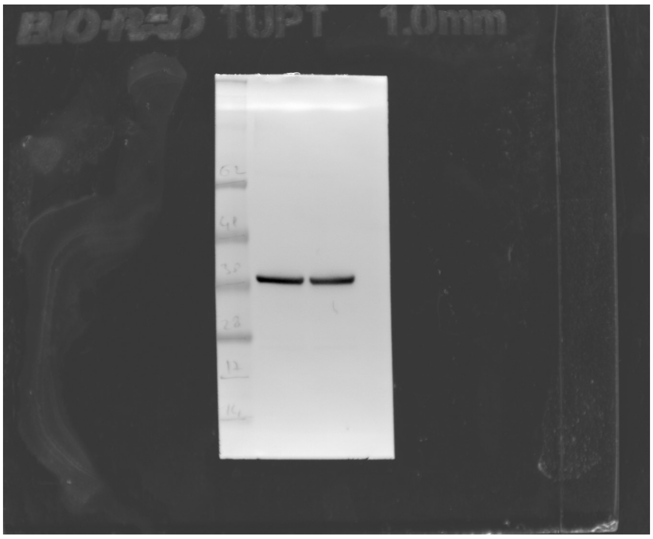

First line: WT, second lane: SCN1A^{mild}

HSPA8 – 71 kDa (1° replicate)

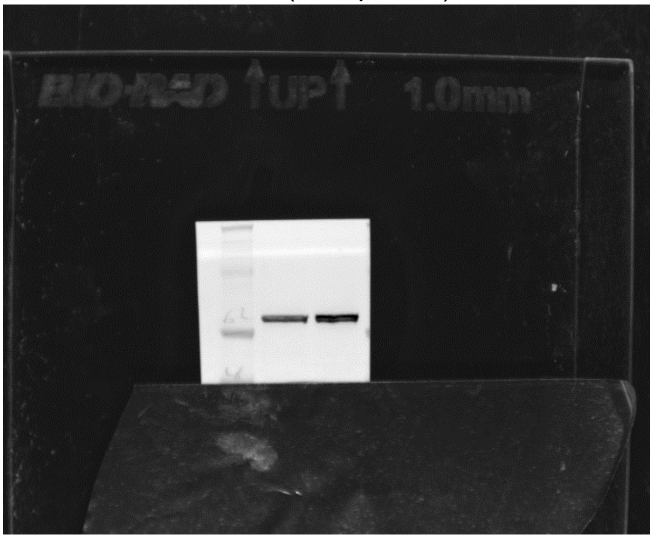

GAPDH – 36 kDa (1° replicate

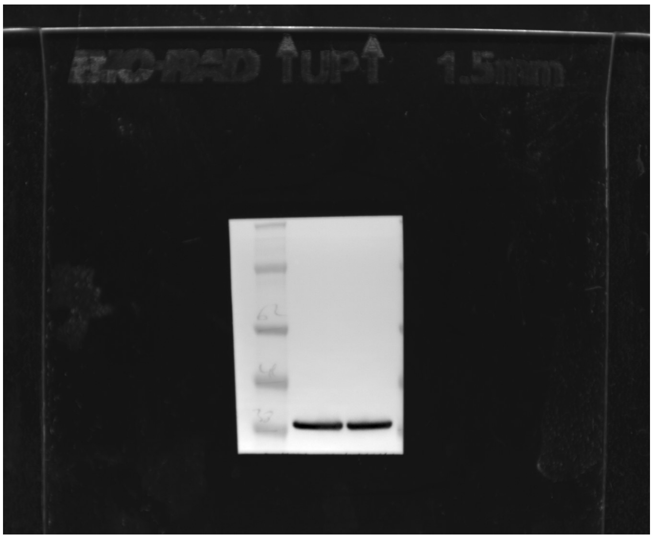

HSPA8 (2° replicate)

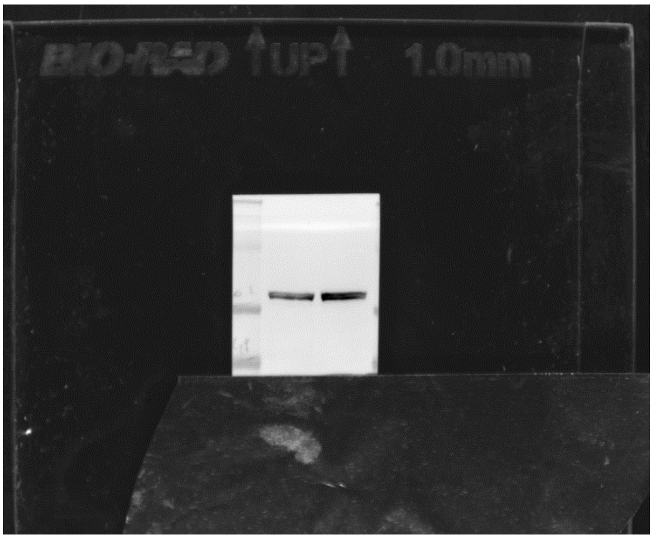

GAPDH (2° replicate)

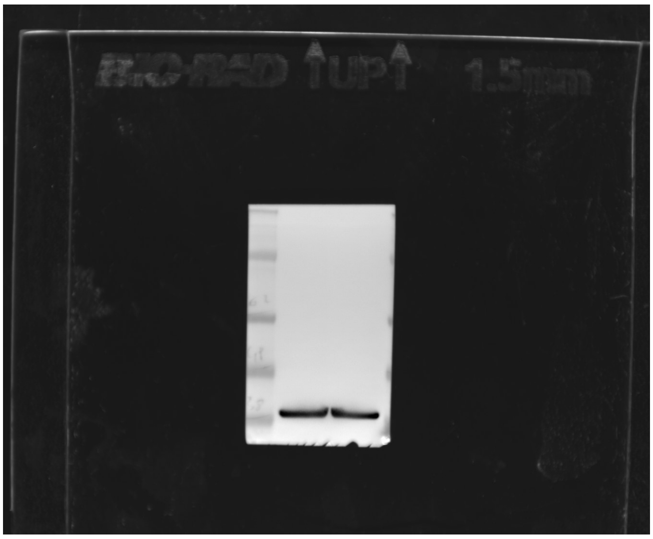

HSPA8 (3° replicate)

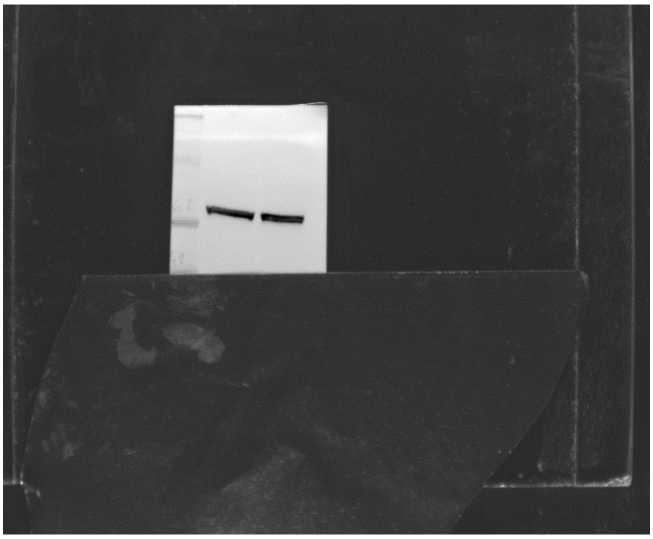

GAPDH (3° replicate)

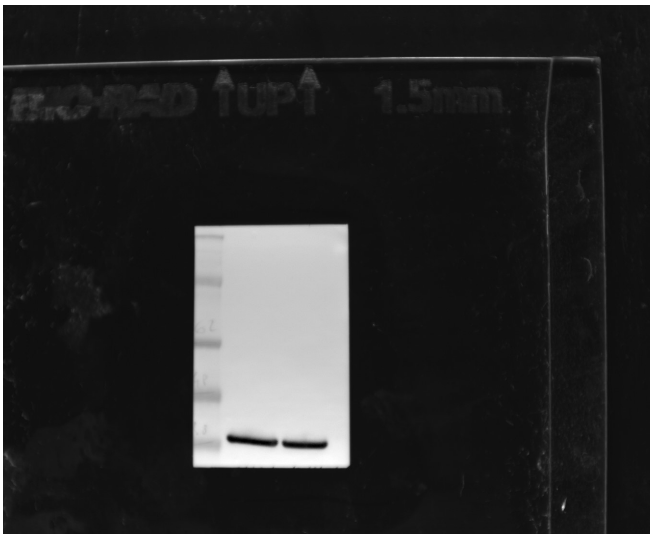

Images showed in FIG. Supplementary 6a

First line: SCN1A^{severe} untreated, second lane: SCN1A^{severe} 12h AA 100uM tr., third lane: SCN1A^{severe} 12h AA 200uM tr., fourth lane: SCN1A^{severe} 12h AA 400uM tr., fifth lane: SCN1A^{severe} AA 24h 100uM tr., sixth lane: SCN1A^{severe} 24h AA 200uM tr., seventh lane: SCN1A^{severe} 24h AA 400uM tr.

HSPB1 – 27 kDa

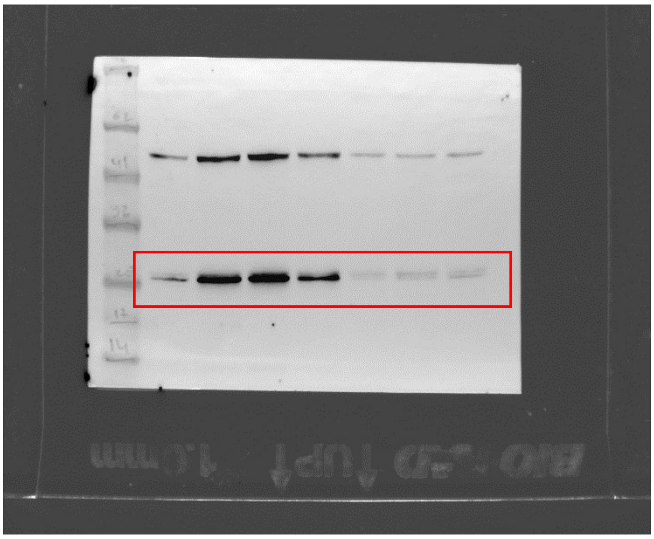

pHSPB1 – 27 kDa

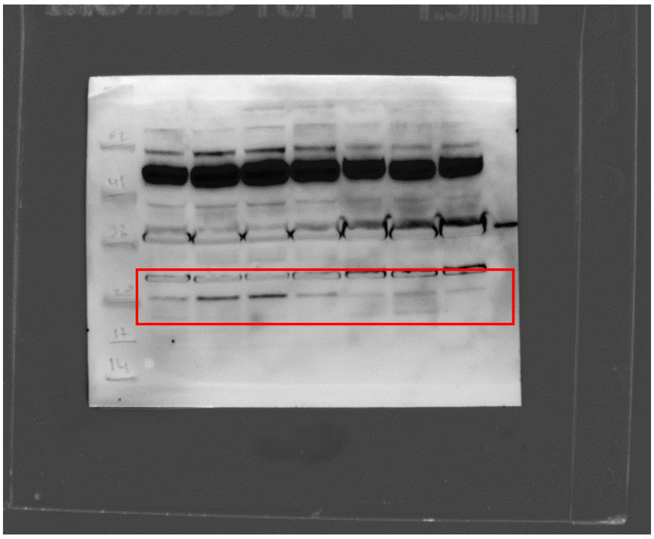

p62 – 62 kDa

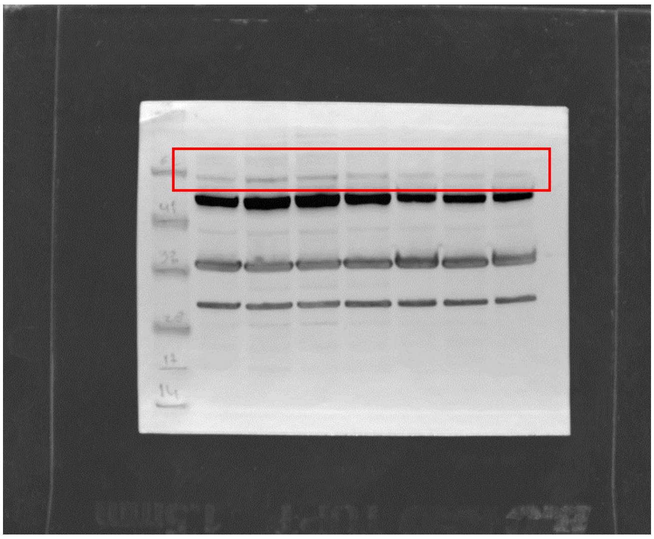

GAPDH – 36 kDa

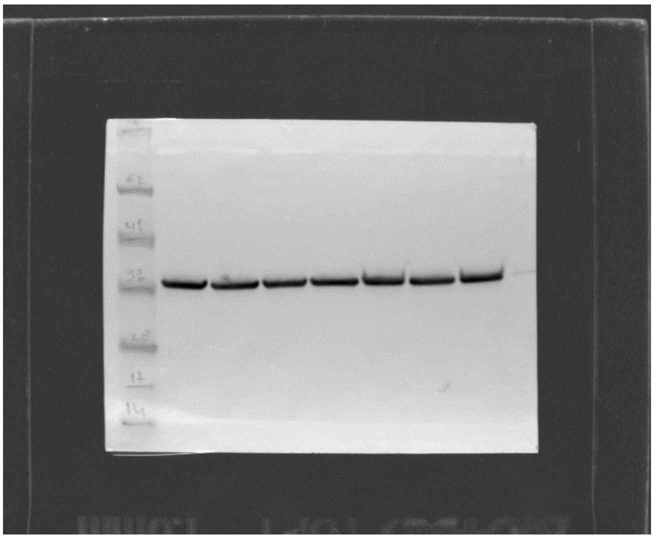

Supplement: Supplementary file 1 — Supplementary Information. [file 41598_2024_56680_MOESM1_ESM.pdf]
